# National and International Clinical Guidelines for the Management of Obesity in Adults: A Systematic Review

**DOI:** 10.1111/obr.70163

**Published:** 2026-05-22

**Authors:** Paul Doody, Ruth Martin, Barrie Tyner, Marie Carrigan, Celine Larkin, Michelle O'Neill, Susan M. Smith, Máirín Ryan

**Affiliations:** ^1^ Discipline of Public Health and Primary Care, Institute of Population Health, School of Medicine, Trinity College Dublin The University of Dublin Dublin Ireland; ^2^ Centre in Ireland for Clinical Guideline Support and Evidence Reviews (CICER), Health Technology Assessment Directorate Health Information and Quality Authority (HIQA) Ireland

**Keywords:** clinical guideline, obesity, overweight, patient involvement, weight management

## Abstract

Obesity is a leading risk factor for premature morbidity and mortality, and an increasing public health challenge. This review aimed to systematically identify and appraise national and international clinical guidelines for the management of obesity in adults. We systematically searched MEDLINE, CINAHL, Embase, and PsycINFO from 01/01/2019 to 06/01/2025. We conducted supplementary searches on websites of clinical guideline‐producing organizations and other relevant institutional and evidence‐based clinical resources. We included national and international guidelines on the management of obesity in adults aged ≥ 18 years that included recommendations on prespecified primary topics of interest, clearly stated the systematic approach and evidence base underpinning recommendations, and included a rating of the quality of evidence. The Appraisal of Guidelines for Research and Evaluation (AGREE) II tool and appraisal using the mEaningful VOice in the design and deLiVery of care (EVOLVE) framework were employed by two reviewers independently. Data were extracted by one reviewer, checked by another, and narratively synthesized. We identified 10 eligible clinical guidelines. The mean overall AGREE II score was 4.9/7 (SD: 1.22). Patients were involved in highlighting preferences, panel membership, reviewing drafts, and consensus meetings in most guidelines, and formulating/prioritizing questions, priority setting, and dissemination in a minority. The 2025 National Institute of Health and Care Excellence guideline on “Overweight and Obesity Management” displayed the most extensive scope and highest overall AGREE II score (7/7). This review will inform adaptation, as part of an ADAPTE process, for a prioritized National Clinical Guideline on the management of obesity in adults in Ireland.

## 1 Introduction

Obesity is a leading risk factor for premature morbidity and mortality, and a major public health challenge [1, 2, 3]. Globally, it is estimated that over 16% of adults now live with obesity; a twofold relative and fourfold absolute increase in the last 30 years [4, 5]. By 2050, if trends including policy changes and interventions continue at their historical pace, it is estimated that globally, over 30% of adults aged ≥ 25 years will be living with obesity [6].

Clinical practice guidelines are “systematically developed statements about specific health problems, intended to assist practitioners and patients in making decisions” [7]. In 2019, a systematic review on clinical guidelines for the management of overweight and obesity in adults identified and appraised 19 clinical guidelines published since 2011, with nine focusing on the general adult population [8]. Promising advances have been made in obesity treatment and management in the succeeding years [9, 10, 11], and correspondingly, there have been several recent systematic and scoping reviews on various aspects of clinical guidelines for the management of obesity [12, 13, 14, 15, 16].

A 2020 systematic review examining definitions of success in clinical practice guidelines for the management of overweight and obesity in adults found 16 guidelines published up to 2020, including three for the general adult population published between 2019 and 2020 [14]. A 2024 scoping review examining clinical practice guidelines for the management of overweight and obesity found 38 guidelines published between 2010 and 2022, including 18 guidelines published between 2019 and 2022, of which eight were for the general adult population [12]. There have also been recent systematic reviews on clinical practice guidelines regarding specific aspects of obesity management and treatment [15, 16]. A review of clinical guidelines for bariatric and metabolic surgery identified 26 guidelines published between 2010 and 2022, including 12 published between 2019 and 2022 [15]. Another review examining the quality of metabolic and bariatric surgery clinical practice guidelines identified eight guidelines for the general adult population, of which four were published between 2019 and 2020 [16].

Despite contemporary advances in obesity management and several recent reviews, no review since 2019 has systematically identified and critically appraised clinical guidelines for the management of obesity in the general adult population. The present review was conducted to inform adaptation, as part of an “ADAPTE” process [7], for a prioritized National Clinical Guideline on the management of obesity in adults in Ireland.

## 2 Materials and Methods

Our aim was to systematically identify and appraise national and international clinical guidelines for the management of obesity in adults published since 2019. Our findings are reported in accordance with the Preferred Reporting Items for Systematic reviews and Meta‐Analyses (PRISMA) statement checklist (Table S1) [17]. We prospectively registered our protocol on the International PROSPEctive Register Of systematic reviews (PROSPERO) (registration number: CRD42025641371), and published the protocol on the Health Information and Quality Authority of Ireland (HIQA) website [18]. Our research question was “What relevant clinical guidelines on the management of obesity in adults (aged ≥ 18 years) are currently in use nationally and internationally?”

### 2.1 Data Sources and Searches

We systematically searched MEDLINE, CINAHL, EMBASE, and PsycINFO using synonyms for “obesity” and “guidelines” from 01/01/2019 to 06/01/2025 (the MEDLINE search strategy is available in Table S2, and the full search strategy is available on Zenodo: https://zenodo.org/records/14637594). The search strategy was developed by a medical librarian and peer‐reviewed by an external librarian using the Peer Review of Electronic Search Strategies (PRESS) tool [19].

We conducted supplementary searches on the websites of 52 pre‐identified clinical guideline‐producing organizations or relevant institutional and evidence‐based clinical resources using a site‐specific Google search string ending: “(guideline* OR guidance OR recommendation) (obesity OR overweight OR over‐weight OR adiposity) adult” between 16/01/2025 and 31/01/2025 (Table S1).

### 2.2 Study Eligibility and Selection

We uploaded search results to Endnote 21 and removed duplicates. We uploaded the remaining records to Covidence systematic review software (Veritas Health Innovation, Melbourne, Australia; available at www.covidence.org), which removed further duplicates. We screened search results against eligibility criteria in two stages: (1) title and abstract and (2) full text. Title and abstracts and full texts were independently screened by two reviewers. Reviewers met to discuss any disagreements, consulting a third reviewer where necessary. Records in Covidence that were not themselves guidelines but mentioned a potentially relevant guideline in their title or abstract were tagged, and the cited guidelines reviewed for potential eligibility (Table S4). During full‐text screening, the reasons for exclusion were recorded.

Guidelines were eligible for inclusion if they were national or international clinical guidelines on the management of obesity for adults aged ≥ 18 years: included recommendations related to at least one of the pre‐identified topics of interest to the guideline development group (GDG) (specifically, reducing weight bias, i.e., negative attitudes towards, and beliefs about, others due to their weight [20, 21]; medical nutrition therapy; physical activity; psychological and behavioral interventions; pharmacotherapy; bariatric surgery; primary care and primary healthcare; or emerging technologies and virtual medicine in obesity management), clearly stated the systematic approach and evidence base underpinning recommendations (operationally defined as at a minimum stating: the databases, platforms, or other sources on which comprehensive searches were employed; the date of the searches; and sufficient information on the contents of the search strategy to facilitate replication/update [22, 23]), and included a rating of the quality of evidence.

We excluded records that were not published in English; were published prior to 2019; did not include recommendations for adults (minimum age ≥ 18 years); were at a regional, local, or non‐national healthcare organization level; described the management of obesity as part of a guideline on another health condition, or solely in a specific subpopulation; did not clearly state the systematic approach and evidence base that underpinned the guideline's recommendations; did not provide recommendations regarding at least one of the eight prespecified topics of interest listed above; did not include a rating of the quality of evidence that underpinned the recommendation(s); had been superseded by a more recent guideline; or were adopted directly from, or duplicated, another guideline.

We employed a pragmatic prioritization and sequential exclusion hierarchy, following the order in the exclusion criteria listed above, i.e., if a record was ineligible for inclusion for multiple reasons, we documented the first relevant reason from the list above in the PRISMA flow diagram (Figure 1) [24].

### 2.3 Quality Appraisal

Two reviewers independently assessed guideline quality using the Appraisal of Guidelines for Research and Evaluation (AGREE) II tool [25] and patient involvement using the mEaningful VOice in the design and deLiVery of care (EVOLVE) framework [26].

AGREE II scores were calculated and reported in accordance with the AGREE II manual, including the average percentage score and the overall assessment score [27]. Item score differences of two or more, and overall score differences of one or more, were discussed to resolve the difference, and where necessary a third reviewer was consulted.

Appraisal of the extent to which people living with obesity were involved in the guidelines' development was conducted through assessing the 14 domains of the EVOLVE framework: guideline panel membership; prioritizing questions; research priority setting; developing the scope of the guideline; formulating questions; choosing outcomes; consensus meetings; assessing the certainty of evidence; writing the guideline; highlighting patient preferences; reviewing guideline drafts; actively disseminating guidelines; patient information tools; and instigating guideline updates.

### 2.4 Data Extraction

Data were extracted by one reviewer and checked by another. Extracted data included the guideline development organization and country of origin, title, date of first publication, date of last update, relevant key clinical questions, search dates, relevant populations, overall settings, topics covered, recommendations, strength of recommendations, quality of evidence, rating systems used for recommendations, and evidence to decision processes.

### 2.5 Data Synthesis

Extracted data were reviewed and narratively synthesized by two reviewers.

### 2.6 Deviations From the Protocol

We made two clarifying adjustments to our protocol: (1) Following discussion with the GDG regarding the intended scope of the guideline, this review will inform, we excluded guidelines solely on specific subpopulations, e.g., middle‐aged women; (2) we excluded studies not *published* in the English language, whereas in the protocol, we imprecisely stated we would exclude guidelines not *available* in the English language.

## 3 Results

### 3.1 Search Results

Our systematic database and platform searches yielded 5780 records, of which 2098 were removed as duplicates. We screened the titles and abstracts of the remaining 3682 records, resulting in 95 reports for full‐text screening. Eighty‐six of these reports were excluded due to not clearly stating the systematic approach and evidence base underpinning the recommendations (*n* = 54); not being published in the English language (*n* = 19); being published prior to 2019 (*n* = 5); being conducted solely in a subpopulation (*n* = 4); not including an assessment of the quality of evidence (*n* = 2); not including a topic of interest (*n* = 1); and being superseded by a more recent guideline (*n* = 1) (Table S5).

We identified 68 additional potentially relevant reports through our gray literature searching. Sixty‐seven of these reports were excluded due to not clearly stating the systematic approach and evidence base underpinning the recommendations (*n* = 33); not being published in the English language (*n* = 29); being published prior to 2019 (*n* = 1); not including recommendations for adults (minimum age ≥ 18 years) (*n* = 1); being solely at a local, regional, or non‐national healthcare organization level (*n* = 1); being conducted solely in a subpopulation (*n* = 1); and not including a topic of interest (*n* = 1) (Table S6).

Therefore, full‐text screening of the 163 collated reports resulted in a total of 10 included guidelines (Figure 1) [28, 29, 30, 31, 32, 33, 34, 35, 36, 37].

**FIGURE 1:**
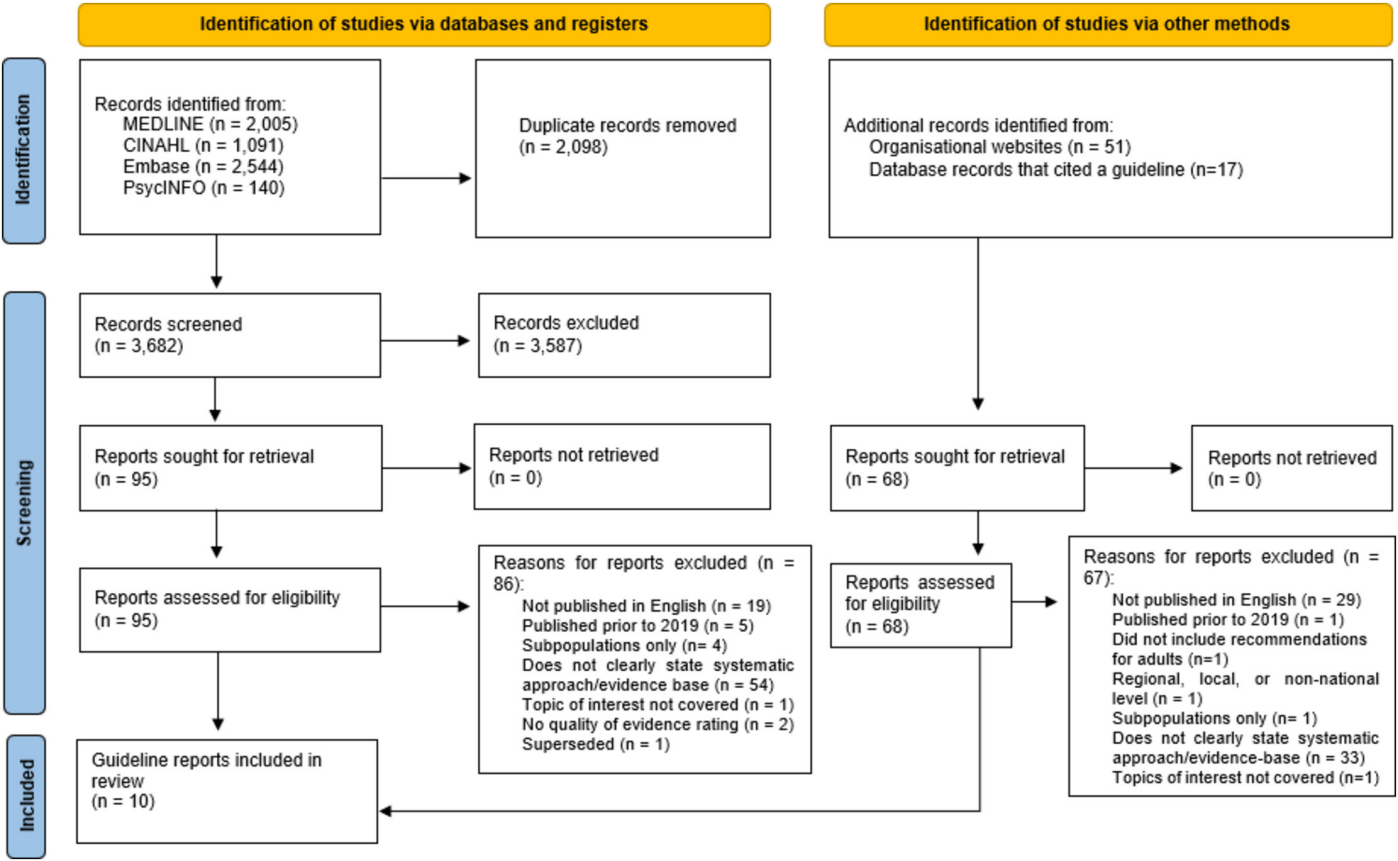
PRISMA flow diagram.

### 3.2 Guideline Characteristics

Of the 10 included guidelines, eight were at a national level [28, 29, 30, 32, 34, 35, 36, 37] and two at an international level [31, 33]. Three guidelines were on obesity management generally [28, 29, 37], four specifically regarding bariatric/metabolic surgery [31, 33, 35, 36], two specifically regarding medical nutrition therapy [30, 34], and one specifically regarding pharmacotherapy [32]. See Table S7 for the full characteristics of included guidelines.

#### 3.2.1 Origin and Developers

Of the 10 included guidelines, four were from the United States (US) [28, 32, 34, 35], two from the United Kingdom (UK) [36, 37], one from Mexico [29], one from South Korea [30], one from Europe [31], and one from Europe and the United States [33]. Two guidelines aligned with national healthcare systems (National Institute of Health and Care Excellence [NICE; England and Wales] and Veterans Affairs [VA]/Department of Defense [DoD; USA]) [28, 37], and eight were produced by national or international societies [29, 30, 31, 32, 33, 34, 35, 36].

#### 3.2.2 Currency

Of the 10 included guidelines, one was published in 2025 (with searches conducted up to 2023 [37]); two in 2024 (with searches conducted up to 2024 [29] and 2022 [33]); one in 2023 (with searches conducted up to 2021 [34]); two in 2022 (with searches conducted up to 2022 [32] and 2021 [30]); one in 2021 (with searches conducted up to 2020 [35]); and three in 2020 (with searches conducted up to 2019 [28] and 2018 [31, 36]). See Table S8 for the details on search dates and clinical questions.

### 3.3 Quality Appraisal

#### 3.3.1 AGREE II Appraisal

The mean overall AGREE II assessment score for the 10 included guidelines was 4.9/7 (SD: 1.22) [28, 29, 30, 31, 32, 33, 34, 35, 36, 37]. The highest overall rating was assigned to the 2025 NICE guideline on “Overweight and Obesity Management [NG246]” [37] with a score of 7/7, followed by the 2022 American Gastroenterological Association (AGA) Clinical Practice Guideline on Pharmacological Interventions for Adults With Obesity [32], and the Clinical Practice Guidelines of the European Association for Endoscopic Surgery (EAES) on Bariatric Surgery: Update 2020 [31] with a score of 6/7. The 2022 Korean Society for the Study of Obesity Consensus Statement on the Effect of Carbohydrate‐Restricted Diets and Intermittent Fasting [30] and the British Obesity and Metabolic Surgery Society Guidelines on Perioperative and Postoperative Biochemical Monitoring and Micronutrient Replacement for Patients Undergoing Bariatric Surgery‐2020 Update [36] were the lowest scoring at 3/7.

The mean score for the rigor of development domain was 67% (standard deviation [SD]: 10%) with the 2022 AGA Clinical Practice Guideline on Pharmacological Interventions for Adults With Obesity [32] scoring the highest at 82%. It should be noted, however, that the AGA guideline is based on a systematic review [38] that has since been retracted [39] and republished [40] as a result of potential overlap in the populations studied. The next highest scoring guideline in the rigor of development domain was the NICE guideline on “Overweight and Obesity Management [NG246]” [37] at 76%.

The mean score for the scope and purpose domain was 93% (SD: 6%), with the 2020 VA/DoD clinical practice guideline for the management of adult overweight and obesity [28], the 2024 Mexican Clinical Practice Guidelines for Adult Overweight and Obesity Management [29], and the 2025 NICE guideline on “Overweight and Obesity Management [NG246]” [37] scoring the highest at 100%. The mean score for the stakeholder involvement domain was 76% (SD: 26%), with the 2025 NICE guideline on “Overweight and Obesity Management [NG246]” [37] scoring the highest at 100%. The mean score for the clarity of presentation domain was 94% (SD: 9%) with the 2022 AGA Clinical Practice Guideline on Pharmacological Interventions for Adults With Obesity [32], the 2023 Academy of Nutrition and Dietetics Guideline on Medical Nutrition Therapy Interventions for Adult Overweight and Obesity [34], and the British Obesity and Metabolic Surgery Society Guidelines on Perioperative and Postoperative Biochemical Monitoring and Micronutrient Replacement for Patients Undergoing Bariatric Surgery‐2020 Update [36] scoring the highest at 100%. The mean score for the applicability domain was 30% (SD: 27%) with the 2025 NICE guideline on “Overweight and Obesity Management [NG246]” [37] scoring the highest at 98%. The mean score for the editorial independence domain was 57% (SD: 16%) with the 2024 Mexican Clinical Practice Guidelines for Adult Overweight and Obesity Management [29] scoring the highest at 79% (Tables 1 and S9).

**TABLE 1:** AGREE II quality appraisal of included guidelines.

Guideline (country)	Domain 1 Scope and purpose	Domain 2 Stakeholder involvement	Domain 3 Rigor of development	Domain 4 Clarity of presentation	Domain 5 Applicability	Domain 6 Editorial independence	Overall quality (out of 7)
Overweight and Obesity Management NICE Guideline [NG246] 2025 [37] (England and Wales)	**100%**	**100%**	76%	97%	**98%**	63%	**7**
Mexican Clinical Practice Guidelines for Adult Overweight and Obesity Management 2024 [29] (Mexico)	**100%**	94%	62%	94%	15%	**79%**	5
American Society for Gastrointestinal Endoscopy‐European Society of Gastrointestinal Endoscopy Guideline on Primary Endoscopic Bariatric and Metabolic Therapies for Adults with Obesity 2024 [33] (United States and Europe)	92%	53%	75%	94%	23%	50%	5
Medical Nutrition Therapy Interventions Provided by Dietitians for Adult Overweight and Obesity Management: An Academy of Nutrition and Dietetics Evidence‐Based Practice Guideline 2023 [34] (United States)	94%	94%	60%	**100%**	31%	71%	5
AGA Clinical Practice Guideline on Pharmacological Interventions for Adults With Obesity 2022 [32] (United States)	92%	81%	**82%**	**100%**	25%	33%	6
Effect of Carbohydrate‐Restricted Diets and Intermittent Fasting on Obesity, Type 2 Diabetes Mellitus, and Hypertension Management: Consensus Statement of the Korean Society for the Study of Obesity, Korean Diabetes Association, and Korean Society of Hypertension 2022 [30] (South Korea)	92%	11%	59%	69%	2%	71%	3
AGA Clinical Practice Guidelines on Intragastric Balloons in the Management of Obesity 2021 [35] (United States)	92%	78%	76%	89%	6%	42%	4
British Obesity and Metabolic Surgery Society Guidelines on Perioperative and Postoperative Biochemical Monitoring and Micronutrient Replacement for Patients Undergoing Bariatric Surgery‐2020 Update [36] (United Kingdom)	78%	67%	47%	**100%**	6%	58%	3
VA/DoD Clinical Practice Guideline for the Management of Adult Overweight and Obesity 2020 [28] (United States)	**100%**	94%	69%	97%	33%	33%	5
Clinical Practice Guidelines of the EAES on Bariatric Surgery: Update 2020 Endorsed by IFSO‐EC, EASO and ESPCOP [31] (Europe)	92%	89%	65%	97%	56%	71%	6

*Note:* Bold text = highest/joint highest scoring in domain; dark green = highest score in domain; medium green = domain score between 75% and 99% (or 5–6 overall) and not highest score in domain; light green = domain score between 50% and 74% (or 4 overall); light orange = domain score between 25% and 49% (or 3 overall); dark orange = domain score between 0% and 24%.

Abbreviations: AGA, American Gastroenterological Association; ESPCOP, European Society for the Perioperative Care of the Obese Patient; IFSO‐EC, International Federation for Surgery of Obesity and Metabolic Disorders—European Chapter; NG, NICE (National Institute of Health and Care Excellence) guideline; NICE, National Institute of Health and Care Excellence; VA/DoD, Veterans Affairs/Department of Defense.

#### 3.3.2 EVOLVE Framework Appraisal

Of the 10 included guidelines, the highest number of EVOLVE framework domains addressed was nine out of 14 [29]; followed by six [33]; four [31, 37]; three [32, 36]; two [35]; one [28, 34], and none [30]. The most common domain addressed was highlighting patient preference, which was included in eight guidelines [28, 29, 31, 32, 33, 35, 36, 37]; six included patients in reviewing the draft guideline [29, 33, 34, 35, 36, 37]; six included patients on guideline panels [29, 31, 32, 33, 36, 37]; five in consensus meetings [29, 31, 32, 33, 37]; three in formulating questions [29, 31, 33]; one in prioritizing questions [29]; one in research priority setting [29]; and one in actively disseminating the guideline [29]. None of the included guidelines reported involving patients in guideline scope development, assessing the certainty of evidence, writing the initial guideline draft, patient information tools, or instigating guideline update (Table 2).

**TABLE 2:** Giving patients a mEaningful VOice in the design and deLiVery of carE (EVOLVE) framework assessment of included guidelines.

Guideline (country)	Guideline panel membership	Prioritize questions	Research priority setting	Guideline scope development	Formulate questions	Choose outcomes	Consensus meeting	Assess certainty of evidence	Write guideline	Highlight patient preference	Review draft guideline	Actively disseminate guideline	Patient information tools	Instigate update
Overweight and Obesity Management NICE Guideline [NG246] 2025 [37] (England and Wales)	Yes	NoNo	No	No	No	No	Yes	No	No	Yes	Yes	No	No	No
Mexican Clinical Practice Guidelines for Adult Overweight and Obesity Management 2024 [29] (Mexico)	Yes	Yes	Yes	No	Yes	Yes	Yes	No	No	Yes	Yes	Yes	No	No
American Society for Gastrointestinal Endoscopy‐European Society of Gastrointestinal Endoscopy Guideline on Primary Endoscopic Bariatric and Metabolic Therapies for Adults with Obesity 2024 [33] (United States and Europe)	Yes	No	No	No	Yes	Yes	Yes	No	No	Yes	Yes	No	No	No
Medical Nutrition Therapy Interventions Provided by Dietitians for Adult Overweight and Obesity Management: An Academy of Nutrition and Dietetics Evidence‐Based Practice Guideline 2023 [34] (United States)	No	No	No	No	No	No	No	No	No	No	Yes	No	No	No
AGA Clinical Practice Guideline on Pharmacological Interventions for Adults With Obesity 2022 [32] (United States)	Yes	No	No	No	No	No	Yes	No	No	Yes	No	No	No	No
Effect of Carbohydrate‐Restricted Diets and Intermittent Fasting on Obesity, Type 2 Diabetes Mellitus, and Hypertension Management: Consensus Statement of the Korean Society for the Study of Obesity, Korean Diabetes Association, and Korean Society of Hypertension 2022 [30] (South Korea)	No	No	No	No	No	No	No	No	No	No	No	No	No	No
AGA Clinical Practice Guidelines on Intragastric Balloons in the Management of Obesity 2021 [35] (United States)	No	No	No	No	No	No	No	No	No	Yes	Yes	No	No	No
British Obesity and Metabolic Surgery Society Guidelines on Perioperative and Postoperative Biochemical Monitoring and Micronutrient Replacement for Patients Undergoing Bariatric Surgery‐2020 update [36] (United Kingdom)	Yes	No	No	No	No	No	No	No	No	Yes	Yes	No	No	No
VA/DoD Clinical Practice Guideline for the Management of Adult Overweight and Obesity 2020 [28] (United States)	No	No	No	No	No	No	No	No	No	Yes	No	No	No	No
Clinical Practice Guidelines of the EAES on Bariatric Surgery: Update 2020 Endorsed by IFSO‐EC, EASO and ESPCOP [31] (Europe)	Yes	No	No	No	Yes	No	Yes	No	No	Yes	No	No	No	No

*Note:* Green = present; red = absent.

Abbreviations: AGA, American Gastroenterological Association; ESPCOP, European Society for the Perioperative Care of the Obese Patient; IFSO‐EC, International Federation for Surgery of Obesity and Metabolic Disorders—European Chapter; NG, NICE (National Institute of Health and Care Excellence) guideline; NICE, National Institute of Health and Care Excellence; VA/DoD, Veterans Affairs/Department of Defense.

### 3.4 Recommendations Within Topic Areas of Interest

Nine of the included guidelines [28, 29, 30, 31, 32, 33, 34, 35, 37] used the Grading of Recommendations, Assessment, Development, and Evaluations (GRADE) framework (or a variation of GRADE), and one [36] used the Scottish Intercollegiate Guidelines Network (SIGN) methodology, both to assess the certainty of the evidence and to move from the evidence to a decision on the strength of each recommendation. Table S10 contains full details regarding the recommendations, strength of recommendation, certainty of evidence, and the evidence to decision process for each of the 10 guidelines. Each of the eight topics of interest was addressed by at least one of the included guidelines, but only one of the guidelines (NICE) [37] addressed all eight topics (Table 3).

#### 3.4.1 Reducing Weight Bias

Two of the 10 guidelines (NICE and the US Academy of Nutrition and Dietetics) included recommendations regarding reducing weight bias (Table 3) [34, 37]. One included weight bias‐specific recommendations in the context of weight management discussions, communications, and follow‐up [37], while the other included weight bias considerations in the context of medical nutrition therapy recommendations [34]. Recommendations within this topic tended to be based on consensus due to a lack of empirical evidence.

#### 3.4.2 Medical Nutrition Therapy

Nine of the 10 guidelines included recommendations regarding medical nutritional therapy (Table 3) [28, 29, 30, 32, 33, 34, 35, 36, 37]. Five guidelines included medical nutrition therapy‐specific recommendations [28, 29, 30, 34, 37] including the following: offering dietary approaches that contribute to a negative energy balance including meal replacements as part of a comprehensive lifestyle intervention, low carbohydrate diets for short‐term (less than six months) weight loss, and against dietary supplements or nutraceuticals for short term weight loss or long‐term weight management [28]; meal plans prescribed by a dietitian, and using traditional and hypoenergetic diets [29]; low and moderately low carbohydrate diets [30]; client‐centered collaborative nutrition care process interventions offered by registered dietitian nutritionists [34]; and flexible individualized interventions that achieve nutritional balance while reducing energy intake including reference to low‐energy and very low‐energy diets [37]. Lack of long‐term studies and study heterogeneity led to withholding recommendation for intermittent fasting in one guideline [30].

Further, two guidelines included medical nutrition therapy considerations as part of lifestyle modification interventions in conjunction with bariatric surgery or metabolic therapy [33, 36]: one as part of lifestyle modification interventions in conjunction with pharmacotherapy [32]; and one as part of lifestyle modification intervention in conjunction with intragastric balloon therapy [35]. The certainty of evidence within this topic ranged from very low to moderate.

#### 3.4.3 Physical Activity

Seven of the 10 guidelines included recommendations regarding physical activity (Table 3) [28, 29, 32, 33, 34, 35, 37]. Three included physical activity‐specific recommendations [28, 29, 37], including aerobic, resistance, and lifestyle physical activity [28]; interventions aimed at controlling sedentary behaviors and using “exercise snacks” [29]; and encouraging adults to increase physical activity (through recommending supervised exercise classes, aiming for a daily step count, or as part of daily life) and reduce time spent inactive even if they do not lose weight as a result [37].

Further, one guideline included physical activity considerations as part of lifestyle modification interventions in conjunction with pharmacotherapy [32], one as part of lifestyle modification interventions in conjunction with bariatric surgery or metabolic therapy [33], one in the context of medical nutrition therapy [34], and one as part of lifestyle modification intervention in conjunction with intragastric balloon therapy [35]. The certainty of evidence within this topic ranged from very low to moderate.

#### 3.4.4 Psychological and Behavioral Interventions

Eight of the 10 guidelines included recommendations regarding psychological and behavioral interventions (Table 3) [28, 29, 32, 33, 34, 35, 36, 37]. Three included specific recommendations [28, 29, 37], including an in‐person group or individual comprehensive lifestyle intervention featuring behavioral components [28]; using behavior change tools; using group psychological interventions over individualized psychological intervention [29]; and encouraging adherence to behavioral management interventions [37].

Further, five guidelines included psychological and behavioral intervention considerations as adjuncts to other interventions: two as part of lifestyle modification interventions in conjunction with bariatric surgery or metabolic therapy [33, 36], one in conjunction with pharmacotherapy [32], one in the context of medical nutrition therapy [34], and one as part of lifestyle modification intervention in conjunction with intragastric balloon therapy [35]. The certainty of evidence within this topic ranged from very low to moderate.

#### 3.4.5 Pharmacotherapy

Four of the 10 guidelines included recommendations regarding pharmacotherapy (Table 3) [28, 29, 32, 37]. In this rapidly evolving area, it is worth noting that pharmacotherapy was discussed in guidelines published in 2020 [28] with searches up to 2019; 2022 [32] with searches up to 2022; 2024 [29] with searches up to 2024; and 2025 [37] with searches up to 2023. Multiple guidelines referred to liraglutide [28, 29, 32, 37], semaglutide [29, 32, 37], naltrexone/bupropion [28, 29, 32], orlistat [28, 32, 37], phentermine/topiramate [28, 32], phentermine [28, 29, 32], and diethylpropion/amfepramone [28, 29, 32]. Certain pharmacotherapies were referenced in one guideline only, such as tirzepatide [37], benzphetamine [28], phendimetrazine [28], fenproporex [29], mazindol [29], clobenzorex [29], antidiabetic medication (sodium‐glucose cotransporter‐2 inhibitors, metformin) [29], and Gelesis 100 oral superabsorbent hydrogel [32]. The content (strength and directionality) of the recommendations, as well as the criteria and conditions for use, varied among the guidelines (further outlined in Table S10). The certainty of evidence within this topic ranged from very low to moderate.

#### 3.4.6 Bariatric Surgery

Six of the 10 guidelines included recommendations regarding bariatric surgery (Table 3) [28, 31, 33, 35, 36, 37]. These included recommendations on the use of metabolic/bariatric surgery in conjunction with a comprehensive lifestyle intervention [28, 33, 35]; varying indications and referral for surgery [28, 31, 33, 35, 37]; medicine while waiting for surgery [37]; preoperative assessment and discussions [31, 36, 37]; perioperative management and discussions [31, 33, 35]; procedures (such as bypass and non‐bypass procedures, adjustable gastric banding [31]; sleeve gastrectomy [31]; Roux‐en‐Y gastric bypass [31]; one‐anastomosis gastric bypass [31]; intragastric balloon [28, 33, 35]; endoscopic gastric remodeling [33]; and aspiration therapies [31, 33]); investigational laparoscopic metabolic and/or bariatric procedures [31]; postoperative care [31, 35]; and longer term follow‐up [31, 35, 37]. The certainty of evidence within this topic ranged from very low to high.

#### 3.4.7 Primary Care and Primary Healthcare

Three of the 10 guidelines included recommendations regarding primary care and primary healthcare (the latter, a broader concept than the former, also incorporating public health measures) (Table 3) [29, 34, 37]. Two guidelines (NICE and the Mexican clinical practice guideline) included primary care and primary healthcare‐specific recommendations [29, 37] including that dietary plans be prescribed by a dietitian rather than a primary care physician [29], and that systems are in place in primary care to implement local strategies including training to provide interventions [37]. The certainty of evidence within this topic ranged from very low to moderate.

#### 3.4.8 Emerging Technologies and Virtual Medicine

Three of the 10 guidelines included recommendations regarding emerging technologies and virtual medicine (Table 3) [28, 34, 37]. Two guidelines (NICE and VA/DoD) included emerging technologies and virtual medicine‐specific recommendations [28, 37] including individual or group‐telephone delivered comprehensive lifestyle interventions as an alternative or in conjunction with in‐person intervention, neither for or against other technology as the primary mode of comprehensive lifestyle intervention delivery [28]; and emphasizing personal choice in the nature of weight management intervention referral, including in‐person individual or group interventions, or digital services depending on the person's preference and needs [37]. The certainty of evidence within this topic ranged from very low to low.

Within the NICE guideline [37], there is signposting to an “early value assessment” on digital technologies for delivering multidisciplinary weight‐management services for use in the UK health system in the interim while awaiting further evidence generation [41]. In addition to listing technologies that can be used to deliver digital weight management services, a range of technologies is outlined that can be used to prescribe and monitor weight management medications [41].

**TABLE 3:** Matrix of obesity management guideline recommendations by topic.

Guideline	Weight bias	Medical nutrition therapy	Physical activity	Psychological and behavioral interventions	Pharmacotherapy	Bariatric surgery	Primary care and primary healthcare	Emerging technologies and virtual medicine
Overweight and Obesity Management NICE Guideline [NG246] 2025 [37] (England and Wales)	Yes	Yes	Yes	Yes	Yes	Yes	Yes	Yes
Mexican Clinical Practice Guidelines for Adult Overweight and Obesity Management 2024 [29] (Mexico)	No	Yes	Yes	Yes	Yes	No^a^	Yes	No
American Society for Gastrointestinal Endoscopy‐European Society of Gastrointestinal Endoscopy Guideline on Primary Endoscopic Bariatric and Metabolic Therapies for Adults with Obesity 2024 [33] (United States and Europe)	No	Yes^b^	Yes^b^	Yes^b^	No	Yes	No	No
Medical Nutrition Therapy Interventions Provided by Dietitians for Adult Overweight and Obesity Management: An Academy of Nutrition and Dietetics Evidence‐Based Practice Guideline 2023 [34] (United States)	Yes^c^	Yes	Yes^c^	Yes^c^	No	No	Yes^c^	Yes^c^
AGA Clinical Practice Guideline on Pharmacological Interventions for Adults With Obesity 2022 [32] (United States)	No	Yes^d^	Yes^d^	Yes^d^	Yes	No	No	No
Effect of Carbohydrate‐Restricted Diets and Intermittent Fasting on Obesity, Type 2 Diabetes Mellitus, and Hypertension Management: Consensus Statement of the Korean Society for the Study of Obesity, Korean Diabetes Association, and Korean Society of Hypertension 2022 [30] (South Korea)	No	Yes	No	No	No	No	No	No
AGA Clinical Practice Guidelines on Intragastric Balloons in the Management of Obesity 2021 [35] (United States)	No	Yes^e^	Yes^e^	Yes^e^	No	Yes	No	No
British Obesity and Metabolic Surgery Society Guidelines on Perioperative and Postoperative Biochemical Monitoring and Micronutrient Replacement for Patients Undergoing Bariatric Surgery‐2020 update [36] (United Kingom)	No	Yes^f^	No	Yes^f^	No	Yes	No	No
VA/DoD Clinical Practice Guideline for the Management of Adult Overweight and Obesity 2020 [28] (United States)	No	Yes	Yes	Yes	Yes	Yes	No	Yes
Clinical Practice Guidelines of the EAES on Bariatric Surgery: Update 2020 Endorsed by IFSO‐EC, EASO and ESPCOP [31] (Europe)	No	No	No	No	No	Yes	No	No

*Note:* Dark green = present; light green = partially present (with further details in footnotes); red = absent.

Abbreviations: AGA, American Gastroenterological Association; ESPCOP, European Society for the Perioperative Care of the Obese Patient; IFSO‐EC, International Federation for Surgery of Obesity and Metabolic Disorders—European Chapter; NICE, National Institute of Health and Care Excellence; VA/DoD, Veterans Affairs/Department of Defense.

^a^ Includes good clinical practice statements.

^b^ As part of lifestyle modification interventions in conjunction with bariatric surgery or metabolic therapy.

^c^ In the context of medical nutrition therapy.

^d^ As part of lifestyle modification interventions in conjunction with pharmacotherapy.

^e^ As part of lifestyle modification intervention in conjunction with intragastric balloon therapy.

^f^ In the context of specialist postoperative dietetic support including support and guidance to achieve long‐term weight loss and weight maintenance.

## 4 Discussion

This review aimed to systematically identify and appraise national and international clinical guidelines for the management of obesity in adults published since 2019, and to our knowledge, is the first systematic review to do this since 2019 [8]. The purpose of the review was to identify relevant high‐quality guidelines suitable for adaptation using the ADAPTE process, requiring guidelines to be sufficiently well‐reported to facilitate replication/update of the evidence base underpinning constituent recommendations.

Each of the eight topics of interest was addressed to varying extents by the included guidelines, with the 2025 NICE guideline on “Overweight and obesity management [NG246]” [37] addressing all eight topics. Nine of the 10 guidelines included recommendations regarding medical nutritional therapy [28, 29, 30, 32, 33, 34, 35, 36, 37], while only two included recommendations regarding weight bias [34, 37]. Nine guidelines used GRADE (or a variation), and one used the SIGN methodology, to assess the certainty of the evidence and move from the evidence to a decision regarding the strength of recommendations. Compared to guidelines in a 2019 systematic review, the included guidelines in the current review were on average, according to the AGREE II tool, of markedly higher quality in the scope and purpose, stakeholder involvement, and clarity of presentation domains; comparable quality in the rigor of development and applicability domains; and lower quality in the editorial independence domain. The latter may be due to the punctilious application of AGREE II criteria and considerations within this domain in the present review. Moreover, the highest scoring guideline in the current review was also of higher‐comparable quality than the highest scoring guideline in the 2019 review [8].

The 2025 NICE guideline on “Overweight and obesity management [NG246]” [37] provided the most extensive coverage of the topics of interest, being the only included guideline to cover all topics. In terms of currency, the NICE guideline was published most recently [37], but its last searches were run in April 2023. Further, the 2025 NICE guideline scored only moderately high in the editorial independence domain, due to the lack of an explicit statement regarding funding. Meanwhile, the Mexican guideline published in 2024 included the most recent searches of any of the identified guidelines (May 2024) but scored lower on rigor of development (62%) [29]. The NICE guideline was the highest scoring overall on AGREE II assessment, and the second highest scoring in the rigor of development domain (76%), surpassed only by the 2022 AGA Clinical Practice Guideline on Pharmacological Interventions for Adults With Obesity (at 82%) [32]. However, it is noteworthy that the AGA guideline is based on a since retracted [38] and republished systematic review [40]. This republication occurred due to potential overlap in the populations studied [39]. However, the AGA guideline has not been updated, and, as a result, adapting this guideline would likely require an examination of the recommendations in light of the findings of the updated systematic review. The NICE GDG used a systematic approach to move from the evidence to decision for each recommendation, considering the relative value placed on the agreed outcomes, the benefits and harms of any interventions, resource use, the overall quality of the evidence, equality implications, and the likely impact on practice or services [37].

The eight topics within scope of this present review were set by the GDG for the National Clinical Guideline on the management of obesity in adults in Ireland. This scope was relatively broad, and our findings are largely consistent with recent systematic and scoping reviews on clinical practice guidelines for the management of obesity [12, 13, 14, 15, 16]. However, we did apply more stringent eligibility criteria than previous reviews, including requiring guidelines to clearly state the systematic approach and evidence base used to underpin the recommendations [42]. These robust eligibility criteria resulted in inclusion only of guidelines that are sufficiently methodologically rigorous and transparent to facilitate adaptation for an upcoming National Clinical Guideline on the management of obesity in adults in Ireland [7, 23]. Prominent guidelines that did not meet eligibility criteria included the Canadian Adult Obesity Clinical Practice Guideline [43] and its national adaptations in Chile [44] and Ireland [45] (and which has also recently been adapted in South Africa) [46]. These were excluded due to not clearly stating the systematic approach and evidence base underpinning recommendations, specifically as the Canadian guideline's search strategies are not freely externally available, and the subsequent national adaptations also did not provide access to these and/or any new or updated search strategies. More recently published guidelines also did not meet our eligibility criteria on necessary steps in evidence synthesis, for example, the international CARDIO Alliance to Improve Disease Outcomes (iCARDIO) Global Implementation Guidelines for the Management of Obesity did not clearly report the systematic approach and evidence base underpinning recommendations [47]. These underscore methodological variation in current obesity guidance, balancing rapid implementation and/or expert consensus with fully documented rigorous evidence synthesis in a rapidly evolving field.

Using the EVOLVE framework, we found that the Mexican clinical guideline [29] involved patients in nine stages, the NICE guideline in four stages [37], and one guideline did not report involving patients at all [30]. Where patients were involved, their role was primarily to highlight patient preference, review the draft guideline, and participate as a panel member. However, they were often excluded from key stages such as scope development and were not given an opportunity to contribute to other stages such as the development of patient information tools due to the absence of these tools in included guidelines.

This review identified a large number of clinical guidelines published recently, and this high volume is expected to continue. For example, a clinical guideline on the management of overweight and obesity in adults, adolescents, and children in Australia is anticipated for publication, and preliminary drafts indicate that this will be within scope and will meet the eligibility criteria of this review once published [48, 49]. The upcoming Australian guideline is of particular interest as it will replace the 2013 National Health and Medical Research Council guideline on the management of overweight and obesity in adults, adolescents, and children in Australia, which scored the highest in terms of quality of all guidelines included in the 2019 systematic review [8, 50]. There are also upcoming guidelines from the American College of Physicians on pharmacologic treatments in adults with overweight and obesity [51]; an Italian Society of Bariatric and Metabolic Surgery for Obesity guideline on endoscopic bariatric treatment of overweight and obesity [52]; and a World Health Organization living guideline on the use and indications of Glucagon‐like peptide‐1 (GLP‐1) receptor agonists for the management of adults living with obesity [53]. However, none at the time of writing have a publicly accessible release date.

To our knowledge, this is the most comprehensive and rigorous review to systematically identify and appraise current national and international clinical guidelines for the management of obesity in adults, and the first to do so in the context of directly informing future adaptation. Moreover, to our knowledge, this is the first review since 2019 to systematically identify and appraise national and international clinical guidelines for the management of obesity in adults [8]. As an additional aspect of the quality appraisal, we assessed the meaningful involvement of people with lived experience of overweight and obesity within the development process of included guidelines.

We included only guidelines published in the English language to ensure accurate interpretation, as this is the sole shared language between reviewers. However, this may have resulted in the exclusion of otherwise eligible guidelines that were not published in English. Moreover, although we conducted a wide‐ranging gray literature search, it is difficult to be truly comprehensive. Moreover, it is evident from the number of recently published clinical guidelines on obesity management and those identified that are currently in progress that this is a rapidly evolving area: Thus, it is possible that guidelines may become outdated relatively quickly. Further, we are aware of one recently published guideline that meets our eligibility criteria: the European Association for the Study of Obesity (EASO) Framework for the Pharmacological Treatment of Obesity [54]; although it was published too late for full inclusion in the present review.

## 5 Conclusion

There are 10 clinical practice guidelines published since 2019 that met inclusion criteria and could be considered for adaptation as part of an ADAPTE process. The guideline with the most extensive scope, and the highest overall AGREE II quality appraisal score, was the UK National Institute of Health and Care Excellence's 2025 guideline on “Overweight and Obesity Management [NG246].”

## Funding

This research is funded by the Health Research Board under grant number ESCG‐2024‐002.

## Conflicts of Interest

The authors declare no conflicts of interest.

## Supporting information


**Table S1:** PRISMA 2020 checklist.
**Table S2:** Search strategy.
**Table S3:** List of organizations searched during gray literature identification.
**Table S4:** Gray literature identification (potential guidelines cited in peer‐reviewed articles).
**Table S5:** Reasons for full‐text exclusion of reports identified through database and platform searches.
**Table S6:** Collated gray literature records for full‐text screening and screening results.
**Table S7:** Characteristics of included guidelines.
**Table S8:** Reported search dates and key clinical questions within scope for included guidelines.
**Table S9:** Quality appraisal of included guidelines (AGREE II).
**Table S10:** Recommendations within scope, certainty of the evidence, strength of recommendations, and evidence to decision process.

## Data Availability

The data that supports the findings of this study are available in the supplementary material of this article.
