# Supplementary material for: National and International Clinical Guidelines for the Management of Obesity in Adults: A Systematic Review
Source: Obes Rev. 2026 May 22;27(12):e70163. doi: 10.1111/obr.70163 (PMC13749261; doi:10.1111/obr.70163)
Supplement: Supplementary file 1 [file OBR-27-e70163-s001.pdf]

Supplementary materials

National and international clinical guidelines for the management of obesity in adults: a systematic review

Paul Doody[1,2*], Ruth Martin[1], Barrie Tyner[1], Marie Carrigan[1], Celine Larkin[1], Michelle O'Neill[1], Susan M. Smith[1,2], Máirín Ryan[1]

[1] Discipline of Public Health and Primary Care, Institute of Population Health, School of Medicine, Trinity College Dublin, the University of Dublin, Dublin, Ireland

[2] Centre in Ireland for Clinical guideline support and Evidence Reviews (CICER), Health Technology Assessment Directorate, Health Information and Quality Authority (HIQA), Ireland

Corresponding author: Dr Paul Doody (paul.doody@tcd.ie / pdoody@hiqa.ie), Discipline of Public Health and Primary Care, Institute of Population Health, School of Medicine, Trinity College Dublin, the University of Dublin, Russell Centre, Tallaght Cross, Dublin, Ireland, D24 DH74, and the Centre in Ireland for Clinical guideline support and Evidence Reviews (CICER), Health Technology Assessment Directorate, Health Information and Quality Authority (HIQA), Ireland.

Supplementary table 1
Supplementary table 2
Supplementary table 3
Supplementary table 4
Supplementary table 5
Supplementary table 6
Supplementary table 7
Supplementary table 8
Supplementary table 9
Supplementary table 10

Supplementary table 1. PRISMA 2020 checklist

Section and Topic	Item #	Checklist item	Location where item is reported
**TITLE**			
Title	1	Identify the report as a systematic review.	Cover page
**ABSTRACT**			
Abstract	2	See the PRISMA 2020 for Abstracts checklist.	1
**INTRODUCTION**			
Rationale	3	Describe the rationale for the review in the context of existing knowledge.	1-2
Objectives	4	Provide an explicit statement of the objective(s) or question(s) the review addresses.	3
**METHODS**			
Eligibility criteria	5	Specify the inclusion and exclusion criteria for the review and how studies were grouped for the syntheses.	4-5
Information sources	6	Specify all databases, registers, websites, organisations, reference lists and other sources searched or consulted to identify studies. Specify the date when each source was last searched or consulted.	3-4
Search strategy	7	Present the full search strategies for all databases, registers and websites, including any filters and limits used.	3, Supplementary Table 2
Selection process	8	Specify the methods used to decide whether a study met the inclusion criteria of the review, including how many reviewers screened each record and each report retrieved, whether they worked independently, and if applicable, details of automation tools used in the process.	4-5
Data collection process	9	Specify the methods used to collect data from reports, including how many reviewers collected data from each report, whether they worked independently, any processes for obtaining or confirming data from study investigators, and if applicable, details of automation tools used in the process.	5-6
Data items	10a	List and define all outcomes for which data were sought. Specify whether all results that were compatible with each outcome domain in each study were sought (e.g. for all measures, time points, analyses), and if not, the methods used to decide which results to collect.	6
	10b	List and define all other variables for which data were sought (e.g. participant and intervention characteristics, funding sources). Describe any assumptions made about any missing or unclear information.	6
Study risk of bias assessment	11	Specify the methods used to assess risk of bias in the included studies, including details of the tool(s) used, how many reviewers assessed each study and whether they worked independently, and if applicable, details of automation tools used in the process.	5-6
Effect measures	12	Specify for each outcome the effect measure(s) (e.g. risk ratio, mean difference) used in the synthesis or presentation of results.	6
Synthesis methods	13a	Describe the processes used to decide which studies were eligible for each synthesis (e.g. tabulating the study intervention characteristics and comparing against the planned groups for each synthesis (item #5)).	N/A
	13b	Describe any methods required to prepare the data for presentation or synthesis, such as handling of missing summary statistics, or data conversions.	6
	13c	Describe any methods used to tabulate or visually display results of individual studies and syntheses.	6

Section and Topic	Item #	Checklist item	Location where item is reported
	13d	Describe any methods used to synthesize results and provide a rationale for the choice(s). If meta-analysis was performed, describe the model(s), method(s) to identify the presence and extent of statistical heterogeneity, and software package(s) used.	6
	13e	Describe any methods used to explore possible causes of heterogeneity among study results (e.g. subgroup analysis, meta-regression).	N/A
	13f	Describe any sensitivity analyses conducted to assess robustness of the synthesized results.	N/A
Reporting bias assessment	14	Describe any methods used to assess risk of bias due to missing results in a synthesis (arising from reporting biases).	N/A
Certainty assessment	15	Describe any methods used to assess certainty (or confidence) in the body of evidence for an outcome.	N/A
**RESULTS**			
Study selection	16a	Describe the results of the search and selection process, from the number of records identified in the search to the number of studies included in the review, ideally using a flow diagram.	7, Figure 1
	16b	Cite studies that might appear to meet the inclusion criteria, but which were excluded, and explain why they were excluded.	7, Supplementary Table 5 and 6
Study characteristics	17	Cite each included study and present its characteristics.	7, 8, Supplementary Table 7
Risk of bias in studies	18	Present assessments of risk of bias for each included study.	8-11, Supplementary Table 9
Results of individual studies	19	For all outcomes, present, for each study: (a) summary statistics for each group (where appropriate) and (b) an effect estimate and its precision (e.g. confidence/credible interval), ideally using structured tables or plots.	8-16
Results of syntheses	20a	For each synthesis, briefly summarise the characteristics and risk of bias among contributing studies.	N/A
	20b	Present results of all statistical syntheses conducted. If meta-analysis was done, present for each the summary estimate and its precision (e.g. confidence/credible interval) and measures of statistical heterogeneity. If comparing groups, describe the direction of the effect.	8-16
	20c	Present results of all investigations of possible causes of heterogeneity among study results.	N/A
	20d	Present results of all sensitivity analyses conducted to assess the robustness of the synthesized results.	N/A
Reporting biases	21	Present assessments of risk of bias due to missing results (arising from reporting biases) for each synthesis assessed.	N/A
Certainty of evidence	22	Present assessments of certainty (or confidence) in the body of evidence for each outcome assessed.	N/A
**DISCUSSION**			
Discussion	23a	Provide a general interpretation of the results in the context of other evidence.	17-20
	23b	Discuss any limitations of the evidence included in the review.	20

Section and Topic	Item #	Checklist item	Location where item is reported
	23c	Discuss any limitations of the review processes used.	20
	23d	Discuss implications of the results for practice, policy, and future research.	19-20
**OTHER INFORMATION**			
Registration and protocol	24a	Provide registration information for the review, including register name and registration number, or state that the review was not registered.	1, 3
	24b	Indicate where the review protocol can be accessed, or state that a protocol was not prepared.	1, 3
	24c	Describe and explain any amendments to information provided at registration or in the protocol.	20
Support	25	Describe sources of financial or non-financial support for the review, and the role of the funders or sponsors in the review.	20
Competing interests	26	Declare any competing interests of review authors.	20
Availability of data, code and other materials	27	Report which of the following are publicly available and where they can be found: template data collection forms; data extracted from included studies; data used for all analyses; analytic code; any other materials used in the review.	3, 4, 7, 8, 11, 12, 15, Supplementary Tables

Supplementary table 2. Search strategy.

**Database Name**	Medline Complete via EBSCOhost
**Date search was run**	06 Jan 2025

#	Query	Limiters/Expanders	Last Run Via	Results
S17	S5 AND S16	Limiters - Publication Date: 20190101-<br>Expanders - Apply equivalent subjects<br>Search modes - Proximity	Interface - EBSCOhost Research Databases<br>Search Screen - Advanced Search<br>Database - MEDLINE Complete	2,005
S16	S6 OR S7 OR S8 OR S9 OR S10 OR S11 OR S12 OR S13 OR S14 OR S15	Expanders - Apply equivalent subjects<br>Search modes - Proximity	Interface - EBSCOhost Research Databases<br>Search Screen - Advanced Search<br>Database - MEDLINE Complete	401,013
S15	TI (guidance OR guideline* or consensus or recommendat*)	Expanders - Apply equivalent subjects<br>Search modes - Proximity	Interface - EBSCOhost Research Databases<br>Search Screen - Advanced Search<br>Database - MEDLINE Complete	210,632
S14	AB (care N2 (pathway or pathways)) OR TI (care N2 (pathway or pathways))	Expanders - Apply equivalent subjects<br>Search modes - Proximity	Interface - EBSCOhost Research Databases<br>Search Screen - Advanced Search<br>Database - MEDLINE Complete	11,252
S13	AB (practice N2 (parameter or parameters)) OR TI (practice N2 (parameter or parameters))	Expanders - Apply equivalent subjects<br>Search modes - Proximity	Interface - EBSCOhost Research Databases<br>Search Screen - Advanced Search<br>Database - MEDLINE Complete	2,072
S12	AB (clinical N2 (pathway or pathways)) OR TI (clinical N2 (pathway or pathways))	Expanders - Apply equivalent subjects<br>Search modes - Proximity	Interface - EBSCOhost Research Databases<br>Search Screen - Advanced Search<br>Database - MEDLINE Complete	9,798
S11	PT Consensus Development Conference Guideline OR PT Consensus Development Conference, NIH OR PT Guideline OR PT Practice Guideline	Expanders - Apply equivalent subjects<br>Search modes - Proximity	Interface - EBSCOhost Research Databases<br>Search Screen - Advanced Search<br>Database - MEDLINE Complete	40,163
S10	(MH "Critical Pathways") OR (MH "Health Planning Guidelines")	Expanders - Apply equivalent subjects<br>Search modes - Proximity	Interface - EBSCOhost Research Databases<br>Search Screen - Advanced Search	12,361

			Database - MEDLINE Complete	
S9	(MH "Clinical Protocols")	Expanders - Apply equivalent subjects<br>Search modes - Proximity	Interface - EBSCOhost Research Databases<br>Search Screen - Advanced Search<br>Database - MEDLINE Complete	30,314
S8	(MH "Consensus Development Conferences as Topic") OR (MH "Consensus Development Conferences, NIH as Topic")	Expanders - Apply equivalent subjects<br>Search modes - Proximity	Interface - EBSCOhost Research Databases<br>Search Screen - Advanced Search<br>Database - MEDLINE Complete	3,013
S7	(MH "Practice Guidelines as Topic")	Expanders - Apply equivalent subjects<br>Search modes - Proximity	Interface - EBSCOhost Research Databases<br>Search Screen - Advanced Search<br>Database - MEDLINE Complete	130,834
S6	(MH "Guidelines as Topic+")	Expanders - Apply equivalent subjects<br>Search modes - Proximity	Interface - EBSCOhost Research Databases<br>Search Screen - Advanced Search<br>Database - MEDLINE Complete	176,435
S5	S1 OR S2 OR S3 OR S4	Expanders - Apply equivalent subjects<br>Search modes - Proximity	Interface - EBSCOhost Research Databases<br>Search Screen - Advanced Search<br>Database - MEDLINE Complete	575,312
S4	AB adipos* OR TI adipos*	Expanders - Apply equivalent subjects<br>Search modes - Proximity	Interface - EBSCOhost Research Databases<br>Search Screen - Advanced Search<br>Database - MEDLINE Complete	143,301
S3	AB (overweight OR over-weight) OR TI (overweight OR over-weight)	Expanders - Apply equivalent subjects<br>Search modes - Proximity	Interface - EBSCOhost Research Databases<br>Search Screen - Advanced Search<br>Database - MEDLINE Complete	96,397
S2	AB obes* OR TI obes*	Expanders - Apply equivalent subjects<br>Search modes - Proximity	Interface - EBSCOhost Research Databases<br>Search Screen - Advanced Search<br>Database - MEDLINE Complete	412,118
S1	(MH "Obesity+")	Expanders - Apply equivalent subjects<br>Search modes - Proximity	Interface - EBSCOhost Research Databases<br>Search Screen - Advanced Search<br>Database - MEDLINE Complete	278,651

Supplementary table 3. List of organisations searched during grey literature identification.

Organisational name<br>URL	No. of results	Date	Additional potentially relevant guideline(s) identified
Agency for Healthcare Research and Quality, US<br>https://www.ahrq.gov/prevention/guidelines	1	20/01/2025	N/A
Academy of Nutrition and Dietetics<br>https://www.eatrightpro.org/	50 (5 pages)	20/01/2025	1. Adult Weight Management (AWM) Guideline (2021-22)<br>2. Medical Nutrition Therapy Interventions Provided by Dietitians for Adult Overweight and Obesity Management: An Academy of Nutrition and Dietetics Evidence-Based Practice Guideline<br>3.Position of the Academy of Nutrition and Dietetics: Medical Nutrition Therapy Behavioral Interventions Provided by Dietitians for Adults With Overweight or Obesity, 2024
American Association of Clinical Endocrinologists and American College of Endocrinology, US<br>https://pro.aace.com/clinical-guidance	50 (5 pages)	17/01/2025	1. American Association of Clinical Endocrinology Consensus Statement: Addressing Stigma and Bias in the Diagnosis and Management of Patients with Obesity/Adiposity-Based Chronic Disease and Assessing Bias and Stigmatization as Determinants of Disease Severity 2023<br>2. Clinical Practice Guidelines For The Perioperative Nutrition, Metabolic, and Nonsurgical Support of Patients Undergoing Bariatric Procedures – 2019 Update: Cosponsored By American Association of Clinical Endocrinologists/American College of Endocrinology, The Obesity Society, American Society For Metabolic & Bariatric Surgery, Obesity Medicine Association, and American Society of Anesthesiologists (Endocrine Practice Volume 25, Supplement 2, December 2019, Pages 1-75)<br>3. AGA Clinical Practice Guideline on Pharmacological Interventions for Adults With Obesity Grunvald, Eduardo et al. Gastroenterology, Volume 163, Issue 5, 1198 - 1225<br>4. Rubino, F., Puhl, R.M., Cummings, D.E. et al. Joint international consensus statement for ending stigma of obesity. Nat Med 26, 485–497 (2020). https://doi.org/10.1038/s41591-020-0803-x
American College of Cardiology<br>https://www.acc.org/Guidelines	50 (5 pages)	17/01/2025	1. Clinical Practice Guidelines For The Perioperative Nutrition, Metabolic, and Nonsurgical Support of Patients Undergoing Bariatric Procedures – 2019 Update<br>2. 2022 AGA Clinical Practice Guideline on Pharmacological Interventions for Adults With Obesity
American Heart Association<br>https://professional.heart.org/	50 (5 pages)	17/01/2025	N/A
Association for the Study of Obesity on the Island of Ireland<br>https://asoi.info/guidelines/	50 (5 pages)	17/01/2025	1. Obesity in Adults: A 2022 Adapted Clinical Practice Guideline for Ireland (18 chapters)
Belgian Health Care Knowledge Centre<br>https://kce.fgov.be/	50 (5 pages)	17/01/2025	N/A
BMJ Best Practice<br>https://bestpractice.bmj.com/info/	50 (5 pages)	17/01/2025	1. Overweight and obesity management, NICE 2025<br>2. 2022 ASMBS and IFSO: indications for metabolic and bariatric surgery<br>3. Pharmacological interventions for adults with obesity, AGA, 2022<br>4. Intragastric balloons in the management of obesity, AGA, 2021<br>5. ASMBS Position Statement on the Rationale for Performance of Upper Gastrointestinal Endoscopy Before and After Metabolic and Bariatric Surgery<br>6. AACE/TOS/ASMBS/OMA/ASA Clinical Practice Guidelines for the Perioperative Nutritional, Metabolic, and

			Nonsurgical Support of the Bariatric Surgery Patient – 2020 Update<br>7. Clinical practice guidelines of the European Association for Endoscopic Surgery (EAES) on bariatric surgery: update 2020 endorsed by IFSO-EC, EASO and ESPCOP<br>8. Obesity: identification, assessment and management, 2023, NICE
Canadian Agency for Drugs and Technologies in Health<br>https://www.cadth.ca/	50 (5 pages)	20/01/2025	1. Guidelines for Canadian bariatric surgical and medical centres: a statement from the Canadian Association of Bariatric Physicians and Surgeons, 2022<br>2. Canadian Adult Obesity Clinical Practice Guidelines<br>3. The practical guide: identification, evaluation, and treatment of overweight and obesity in adults
Canadian Task Force on Preventive Health Care<br>https://canadiantaskforce.ca/	50 (5 pages)	20/01/2025	N/A
Clinical Guidelines Committee of the American College of Physicians<br>https://www.acponline.org/	50 (5 pages)	14/02/2025	1. American Gastroenterological Association pharmacologic management of obesity in adults guideline in 2022
Danish Health Authority<br>https://www.sst.dk/en/english/	50 (5 pages)	20/01/2025	N/A
Department of Health (including National Clinical Guidelines), Ireland<br>http://health.gov.ie	1 (1 page)	20/01/2025	N/A
Department of Veterans Affairs and Department of Defense, US<br>https://www.healthquality.va.gov/	50 (5 pages)	20/01/2025	1. VA/DOD Clinical Practice Guidelines Management of Adult Overweight and Obesity (OBE) (2020)
Endocrine Society<br>https://www.endocrine.org/	50 (5 pages)	20/01/2025	N/A
European Association for the Study of Obesity<br>https://easo.org/clinical-guidelines/	50 (5 pages)	20/01/2025	1. European Practical and Patient-Centred Guidelines for Adult Obesity Management in Primary Care 2019<br>2. European Society of Endocrinology Clinical Practice Guideline: Endocrine work-up in obesity 2020<br>3. European Guidelines for Obesity Management in Adults with a Very Low-Calorie Ketogenic Diet: A Systematic Review and Meta-Analysis 2021<br>4. European Association for the Study of Obesity Position Statement on Medical Nutrition Therapy for the Management of Overweight and Obesity in Adults Developed in Collaboration with the European Federation of the Associations of Dietitians 2022<br>5. National guidelines for obesity care, Swedish guideline 2023<br>6. Overweight and obesity in adults and children, Dutch Association of Medical Specialists, Netherlands 2023<br>7. Obesity (children, adolescents and adults), Finland 2024<br>8. Therapy of overweight and obesity resistant to behavioral treatment in the adult population with metabolic comorbidities Italy 2023<br>9. (Clinical guidelines on the treatment of adults individuals with obesity) – Iceland<br>10. Non-surgical treatment of severe obesity – Denmark<br>11. (Obesity Diagnosis and Treatment Guide) – Türkiye<br>12. (Adult obesity: 2nd and 3rd level support) HAS<br>13. Belgium Association for the Study of Obesity (BASO) treatment guidelines

			14. Surgical treatment of obesity, Dutch Association of Medical Specialists, Netherlands 2020<br>15. Obesity in Adults: Position Statement of Polish Association for the Study on Obesity, Polish Association of Endocrinology, Polish Association of Cardiodiabetology, Polish Psychiatric Association, Section of Metabolic and Bariatric Surgery of the Association of Polish Surgeons, and the College of Family Physicians in Poland<br>16. (Guidelines for prevention and the fight against overweight and obesity)" – Italy<br>17. Standard diagnostic and therapeutic procedure for the complex management of overweight/obesity in adulthood 1st revision – Slovakia
Federation Medical Specialists, Netherlands<br>https://richtlijnendatabase.nl/	50 (5 pages)	20/01/2025	1. Qatar obesity guidelines for adults 2022<br>2. National Clinical Guideline: Bariatric and Metabolic Surgery in Adult Qatar 2021<br>Updated guidelines available online March 2025 (National Clinical Guidelines: The Diagnosis & Management of Obesity in Adults, Ministry of Public Health Qatar, 2024; National Clinical Guidelines: Metabolic And Bariatric Surgery In Adults, Ministry of Public Health Qatar, 2025)
https://www.kaypahoito.fi/suositukset	50 (5 pages)	20/01/2025	N/A
French National Authority for Health<br>https://www.has-sante.fr	50 (5 pages)	20/01/2025	1. Care pathway guide: overweight and obesity in adults (summary) 2024 HAS<br>2. Obesity in adults: Second and third-level management Part II: before and after bariatric surgery (toolkit guide)
Geneva Foundation for Medical Education and Research<br>https://www.gfmer.ch/	50 (5 pages)	20/01/2025	1. European Practical and Patient-Centred Guidelines for Adult Obesity Management in Primary Care 2019<br>2. European Society of Endocrinology Clinical Practice Guideline: Endocrine work-up in obesity 2020<br>3. European Guidelines for Obesity Management in Adults with a Very Low-Calorie Ketogenic Diet: A Systematic Review and Meta-Analysis 2021<br>4. European Association for the Study of Obesity Position Statement on Medical Nutrition Therapy for the Management of Overweight and Obesity in Adults Developed in Collaboration with the European Federation of the Associations of Dietitians 2022<br>5. National guidelines for obesity care, Swedish guideline 2023<br>6. Overweight and obesity in adults and children, Dutch Association of Medical Specialists, Netherlands 2023<br>7. Obesity (children, adolescents and adults), Finland 2024<br>8. Therapy of overweight and obesity resistant to behavioral treatment in the adult population with metabolic comorbidities Italy 2023<br>9. (Clinical guidelines on the treatment of adults individuals with obesity) – Iceland<br>10. Non-surgical treatment of severe obesity – Denmark<br>11. (Obesity Diagnosis and Treatment Guide) – Türkiye<br>12. (Adult obesity: 2nd and 3rd level support) HAS<br>13. Belgium Association for the Study of Obesity (BASO) treatment guidelines<br>14. Surgical treatment of obesity, Dutch Association of Medical Specialists, Netherlands 2020<br>15. Obesity in Adults: Position Statement of Polish Association for the Study on Obesity, Polish Association of Endocrinology, Polish Association of Cardiodiabetology, Polish Psychiatric Association, Section of Metabolic and Bariatric Surgery of the Association of Polish Surgeons, and the College of Family Physicians in Poland<br>16. (Guidelines for prevention and the fight against overweight and obesity)" – Italy<br>17. Standard diagnostic and therapeutic procedure for the complex management of overweight/obesity in adulthood 1st revision – Slovakia

German Association of Scientific Medical Societies https://register.awmf.org/	50 (5 pages)	21/01/2025	1. Guideline for the prevention and treatment of obesity 2024 Germany
German Oncology Guideline Program of the AWMF, DKG and DKH https://www.leitlinienprogramm-onkologie.de	50 (5 pages)	21/01/2025	1. 2022 AGA Clinical Practice Guideline on Pharmacological Interventions for Adults With Obesity<br>2. 2021 AGA Clinical Practice Guidelines on Intragastric Balloons in the Management of Obesity<br>3. 2022 Clinical Practice Guideline for the Management of Overweight and Obesity in Adults in Saudi Arabia
Google https://www.google.com/	100 (10 pages)	21/01/2025	1. European Practical and Patient-Centred Guidelines for Adult Obesity Management in Primary Care 2019<br>2. Mexican Clinical Practice Guidelines for Adult Overweight and Obesity Management<br>3. Clinical Practice Guideline for the Management of Overweight and Obesity in Adults in Saudi Arabia 2022<br>4. American Society for Gastrointestinal Endoscopy–European Society of Gastrointestinal Endoscopy guideline on primary endoscopic bariatric and metabolic therapies for adults with obesity 2024<br>5. The Clinical Practice Guidelines (CPG) for the Management of Obesity Malaysia 2023<br>6. Evaluation and Treatment of Obesity and Its Comorbidities: 2022 Update of Clinical Practice Guidelines for Obesity by the Korean Society for the Study of Obesity
Google Scholar https://scholar.google.com/	100 (10 pages)	21/01/2025	1. Obesity in adults: a clinical practice guideline 2020<br>2. European Society of Endocrinology Clinical Practice Guideline: Endocrine work-up in obesity 2020<br>3. Guidelines for medical nutrition treatment of overweight/obesity in China (2021)<br>4. Medical Nutrition Therapy Interventions Provided by Dietitians for Adult Overweight and Obesity Management: An Academy of Nutrition and Dietetics Evidence-Based Practice Guideline<br>5. Diagnosis of Obesity: 2022 Update of Clinical Practice Guidelines for Obesity by the Korean Society for the Study of Obesity<br>6. 2020 ISPRM/ESPRM guidelines on physical and rehabilitation Medicine professional practice for adults with obesity and related comorbidities<br>7. 2022 AGA Clinical Practice Guideline on Pharmacological Interventions for Adults With Obesity<br>8. 2023 AACE Statement: Addressing Stigma and Bias in the Diagnosis and Management of Patients with Obesity/Adiposity-Based Chronic Disease and Assessing Bias and Stigmatization as Determinants of Disease Severity
Guia Salud, Spain https://portal.guiasalud.es/gpc/	50 (5 pages)	21/01/2025	N/A
Guideline Central https://www.guidelinecentral.com/	50 (5 pages)	21/01/2025	1. Adult Weight Management (AWM) Guideline (2021-22)<br>2. Clinical Practice Guidelines For The Perioperative Nutrition, Metabolic, and Nonsurgical Support of Patients Undergoing Bariatric Procedures – 2019 Update: Cosponsored By American Association of Clinical Endocrinologists/American College of Endocrinology, The Obesity Society, American Society For Metabolic & Bariatric Surgery, Obesity Medicine Association, and American Society of Anesthesiologists<br>3. 2023 IFSO-WGO Obesity Guidelines<br>4. 2022 American Society for Metabolic and Bariatric Surgery (ASMBS) and International Federation for the Surgery of Obesity and Metabolic Disorders (IFSO): Indications for Metabolic and Bariatric Surgery<br>5. Scientific evidence for the updated guidelines on indications for metabolic and bariatric surgery (IFSO/ASMBS)
Guidelines International Network international guideline library and registry of guidelines https://g-i-n.net/international-guidelines-library	0 (0 pages)	21/01/2025	1. Clinical Practice Guideline of Korean Medicine for Obesity 2024
Health Service Executive, Ireland	50 (5	21/01/2025	N/A

www.hse.ie	pages)		
Institute for Clinical Systems Improvement, US https://www.icsi.org/guideline/	Web server was down	21/01/2025	N/A
International Federation for the Surgery of Obesity https://www.ifso.com/	1 (1 page)	21/01/2025	1. 2022 American Society of Metabolic and Bariatric Surgery (ASMBS) and International Federation for the Surgery of Obesity and Metabolic Disorders (IFSO) Indications for Metabolic and Bariatric Surgery<br>2. British Obesity Metabolic Surgery Society Endorsed Guidelines for Psychological Support Pre- and Post-Bariatric Surgery<br>3. Obesity and Metabolic Surgery Society of India (OSSI) Guidelines for Patient and Procedure Selection for Bariatric and Metabolic Surgery 2020<br>4. Clinical Practice Guidelines of the European Association for Endoscopic Surgery (EAES) on Bariatric Surgery: Update 2020 Endorsed by IFSO-EC, EASO and ESPCOP<br>5. British Obesity and Metabolic Surgery Society Guidelines on Perioperative and Postoperative Biochemical Monitoring and Micronutrient Replacement for Patients Undergoing Bariatric Surgery—2020 Update<br>6. EAES Rapid Guideline: Systematic Review, Network Meta-Analysis, CINeMA and GRADE Assessment, and European Consensus on Bariatric Surgery-Extension 2022<br>7. Guidelines for Canadian Bariatric Surgical and Medical Centres: A Statement from the Canadian Association of Bariatric Physicians and Surgeons<br>8. Regional Recommendations for the Treatment and Management of Adult Obesity in the Gulf & Lebanon 2021<br>9. American Society for Gastrointestinal Endoscopy-European Society of Gastrointestinal Endoscopy Guideline on Primary Endoscopic Bariatric and Metabolic Therapies for Adults with Obesity 2024<br>10. Consensus on Definitions and Clinical Practice Guidelines for Patients Considering Metabolic-Bariatric Surgery 2023
Japan Council for Quality Health Care https://minds.jcqhc.or.jp/search/	9 (1 page)	BT 22/01/2025	2020 Joint international consensus statement for ending stigma of obesity
Lenus www.lenus.ie	50 (5 pages)	22/01/2025	1. European recommendations from health care professionals and people living with obesity on safe practice for bariatric and metabolic surgery medical tourism: a modified delphi consensus statement from EASO, IFSO-EC and ECPO. 2024
MAGIC Evidence Ecosystem Foundation https://app.magicapp.org/#/guidelines	50 (5 pages)	22/01/2025	1. EAES Rapid Guideline: Systematic review, network meta-analysis, CINeMA and GRADE assessment, and European consensus on bariatric surgery – Extension 2022 by European Association for Endoscopic Surgery and other Interventional Techniques<br>2. Clinical practice guidelines of the European Association for Endoscopic Surgery (EAES) on bariatric surgery: update 2020 endorsed by IFSO-EC, EASO and ESPCOP
National Academy of Medicine, US https://nam.edu/about-the-nam/	0 (0 pages)	22/01/2025	N/A
National Board of Health and Welfare, Sweden https://www.socialstyrelsen.se/	40 (4 pages)	22/01/2025	N/A
National Health and Medical Research Council Clinical Practice, Australian	50 (5 pages)	22/01/2025	N/A

https://nhmrc.gov.au/about-us/publications			
National Institute for Health and Care Excellence http://www.nice.org.uk	50 (5 pages)	22/01/2025	1. Overweight and obesity management NICE guideline [NG246] 2025
New Zealand Ministry of Health https://www.health.govt.nz/	50 (5 pages)	22/01/2025	N/A
Norwegian Directorate of Health https://www.helsedirektoratet.no/	50 (5 pages)	22/01/2025	N/A
Public Health Agency of Sweden, Sweden https://www.folkhalsomyndigheten.se/	23 (3 pages)	22/01/2025	N/A
Ravijuhend, Estonia https://www.ravijuhend.ee/	39 (4 pages)	22/01/2025	N/A
Royal Australian and New Zealand College of Psychiatrists https://www.ranzcp.org/clinical-guidelines-publications	39 (4 pages)	22/01/2025	N/A
Scottish Intercollegiate Guidelines Network www.sign.ac.uk	50 (5 pages)	22/01/2025	N/A
Singapore Ministry of Health https://www.moh.gov.sg/	37 (4 pages)	22/01/2025	N/A
Socialstyrelsen (Health and Medical Care and Social Services), Sweden https://www.socialstyrelsen.se/english	40 (4 pages)	22/01/2025	N/A
Swiss Centre for International Health https://www.swisstph.ch/en/	0 (0 pages)	22/01/2025	N/A
The Best Practice Advocacy Centre New Zealand, New Zealand https://bpac.org.nz/guidelines/	50 (5 pages)	22/01/2025	N/A
Trip Database https://www.tripdatabase.com/	50 (5 pages)	31/01/2025	1. Definition, Criteria, and Core Concepts of Guidelines for the Management of Obesity Disease in Japan 2024<br>2. Metabolic Dysfunction-Associated Steatotic Liver Disease — Assessment of Patients with Obesity and Metabolic Syndrome — Guideline from the Brazilian Society of Bariatric and Metabolic Surgery<br>3. Peri-Operative Management of Obese Patients in Digestive Surgery: Clinical Practice Guidelines from the French Society of Digestive Surgery<br>4. American Society for Gastrointestinal Endoscopy-European Society of Gastrointestinal Endoscopy Guideline on Primary Endoscopic Bariatric and Metabolic Therapies for Adults with Obesity<br>5. Endoscopic Sleeve Gastroplasty for Obesity<br>6. Tirzepatide for Managing Overweight and Obesity<br>7. European Guidelines on Peri-Operative Venous Thromboembolism Prophylaxis: First Update. Chapter 10: Surgery in the Obese Patient<br>8. SICOB Italian Clinical Practice Guidelines for the Surgical Treatment of Obesity and Associated Diseases Using GRADE Methodology on Bariatric and Metabolic Surgery

			9. Swallowable Gastric Balloon Capsule for Weight Loss<br>10. The Clinical Practice Guidelines (CPG) for the Management of Obesity Malaysian 2023<br>11. Guidelines for Canadian Bariatric Surgical and Medical Centres: A Statement from the Canadian Association of Bariatric Physicians and Surgeons 2022<br>12. Guidelines for the Management of Comorbidities Relevant to Metabolic and Bariatric Surgery SAGES 2024
UpToDate<br>https://www.uptodate.com/	6 (1 page)	05/02/2025	1. ASMBS Updated Position Statement on Sleeve Gastrectomy as a Bariatric Procedure - American Society for Metabolic and Bariatric Surgery<br>2. Endoscopic sleeve gastroplasty for obesity - NICE<br>3. American Society for Gastrointestinal Endoscopy (ASGE)–European Society of Gastrointestinal Endoscopy (ESGE): Guideline on primary endoscopic bariatric and metabolic therapies for adults with obesity (2024)<br>4. ASMBS Updated Position Statement on Single-Anastomosis Duodenal Switch - American Society for Metabolic and Bariatric Surgery<br>5. ASMBS Preoperative Care Pathway for Laparoscopic Roux-en-Y Gastric Bypass - American Society for Metabolic and Bariatric Surgery<br>6. ASMBS Position Statement on the Rationale for Performance of Upper Gastrointestinal Endoscopy Before and After Metabolic and Bariatric Surgery - American Society for Metabolic and Bariatric Surgery<br>7. Postoperative nausea and vomiting in bariatric surgery: a position statement endorsed by the ASMBS and the ISPCOP - American Society for Metabolic and Bariatric Surgery<br>8. ASMBS Updated Position Statement on Metabolic Bone Changes after Bariatric Surgery - American Society for Metabolic and Bariatric Surgery<br>9. Canadian consensus statement: enhanced recovery after surgery in bariatric surgery | Surgical Endoscopy<br>10. American Society for Metabolic and Bariatric Surgery updated statement on single-anastomosis duodenal switch<br>11. ASMBS position statement on preoperative patient optimization before metabolic and bariatric surgery<br>12. American Society of Metabolic and Bariatric Surgery consensus statement on laparoscopic adjustable gastric band management<br>13. Operative management of acute abdomen after bariatric surgery in the emergency setting: the OBA guidelines<br>14. ASMBS Bariatric Emergencies for the General Surgeon Statement<br>15. AGA Clinical Practice Update on Evaluation and Management of Early Complications After Bariatric/Metabolic Surgery: Expert Review<br>16. ASMBS Position Statement on the Relationship Between Obesity and Cancer and the Role of Bariatric Surgery: Risk, Timing of Treatment, Effects on Disease Biology, and Qualification for Surgery<br>17. Nutrition, Physical Activity, and Prescription of Supplements in Pre- and Post-bariatric Surgery Patients: An Updated Comprehensive Practical Guideline
US Preventive Services Task Force<br>https://uspreventiveservicestaskforce.org/uspstf/	50 (5 pages)	31/01/2025	1. Obesity Prevention and Control: Digital Health Interventions for Adolescents with Overweight or Obesity<br>2. Behavioral Weight Loss Interventions to Prevent Obesity-Related Morbidity and Mortality in Adults: Recommendation Statement
World Gastroenterology Organisation<br>https://www.worldgastroenterology.org/guidelines	50 (5 pages)	22/01/2025	1. IFSO-WGO Guidelines on Obesity<br>2. Methodology and Results of a Joint IFSO-WGO Delphi Survey of 94 Intercontinental, Interdisciplinary Experts in Obesity Management
World Obesity Federation	50 (5	30/01/2025	1. Obesity in Adults: A 2022 Adapted Clinical Practice Guideline for Ireland

https://www.worldobesity.org	pages)		2. European practical and patient-centred guidelines for adult obesity management in primary care 2019<br>3. Regional Recommendations for the Treatment and Management of Adult Obesity in the Gulf & Lebanon 2020<br>4. Clinical practice guidelines of the European Association for Endoscopic Surgery (EAES) on bariatric surgery: update 2020 endorsed by IFSO-EC, EASO and ESPCOP<br>5. Mexican Clinical Practice Guidelines for Adult Overweight and Obesity Management 2024<br>6. Obesity in adults: Clinical practice guideline adapted for Chile 2022<br>7. Evidence-based Guideline on Adult Obesity Prevention and Management 2023<br>8. Clinical Guidelines for Management of Obesity in Icelandic Adults (in Icelandic)
World Health Organization<br>www.who.int	50 (5 pages)	17/01/2025	1. Obesity in adults: a clinical practice guideline (Canada)<br>2. European Practical and Patient-Centred Guidelines for Adult Obesity Management in Primary Care<br>3. Clinical practice guidelines of the European Association for Endoscopic Surgery (EAES) on bariatric surgery: update 2020 endorsed by IFSO-EC, EASO and ESPCOP<br>4. Exercise training in the management of overweight and obesity in adults: Synthesis of the evidence and recommendations from the European Association for the Study of Obesity Physical Activity Working Group<br>5. WHO guideline on the use and indications of GLP1 RAs for management of adults living with obesity presently in development https://www.who.int/news-room/articles-detail/public-notice-and-comments-on-the-gdg-for-who-rapid-advice-guideline-on-the-use-and-indications-of-glp1-ras-for-management-of-adults-living-with-obesity

Footnote: Separate search strings were used for each organisation which included url as site specific filter. For example, Agency for Healthcare and Research and Quality, US: https://www.google.com/search?q=site:ahrq.gov/prevention/guidelines+(guideline+OR+guidance+OR+recommendation)+(obesity+OR+overweight+OR+over-weight+OR+adiposity)+adult**

Supplementary table 4. Grey literature identification (potential guidelines cited in peer-reviewed articles).

**Screening source: Tagged articles in Covidence (filtered by author)**		
**Article**	**URL**	**Potential additional guideline(s) identified**
Worksite Health Promotion and the ACSM Consensus Statement on Physical Activity and Excess Body Weight and Adiposity	10.1249/FIT.0000000000001025	N/A
Ultrasound-based measurement of central adiposity: Key considerations and guidelines	10.1111/obr.13716	N/A
How to Dose Vancomycin in Overweight and Obese Patients with Varying Renal (Dys)function in the Novel Era of AUC 400â€“600 mgÂ·h/L-Targeted Dosing	10.1007/s40262-023-01324-5	N/A
NICE recommendations on tirzepatide for weight loss could increase NHS access	10.1136/bmj.q1239	N/A
Use of person-centered language in obesity-related publications across sports medicine journals: a systematic review of adherence to person-centered language guidelines in sports medicine	10.1515/jom-2023-0254	N/A
Low muscle mass and sarcopenic obesity and their relation to comorbidities in a population with class II/III obesity: a study on the diagnostic criteria of the EASO/ESPEN consensus guidelines	10.1159/000538577	N/A
Impact of GLP-1 Receptor Agonists in Gastrointestinal Endoscopy: An Updated Review	10.3390/jcm13185627	1. AGA Clinical Practice Guideline on Pharmacological Interventions for Adults With Obesity, Gastroenterology 2022<br>2. AGA Clinical Practice Guidelines on Intragastric Balloons in the Management of Obesity, Gastroenterology 2021<br>3. Obesity in adults: a clinical practice guideline, CMAJ, 2020
The German guideline "Obesity in pregnancy": comparison with the international approach	10.1007/s00404-023-07314-z	N/A
Validation of a dietary advice protocol for adults with obesity in primary health care according to the Brazilian Dietary Guidelines	10.1093/fampra/cmac132	1.[Clinical Protocol and Therapeutic Guidelines (PCDT) for overweight and obesity in adults], Brazil Ministry of Health, 2022**2.** [Overweight and Obesity in Adults], Brazil Ministry of Health, 2024<br>3. [Manual of Care for Overweight and Obese People in the Scope of Primary Health Care (PHC) of the SUS], Brazil Ministry of Health, 2023

Position of the Academy of Nutrition and Dietetics: Medical Nutrition Therapy Behavioral Interventions Provided by Dietitians for Adults With Overweight or Obesity, 2024	10.1016/j.jand.2023.11.013	1. Medical Nutrition Therapy Interventions provided by Dietitians for Adult Overweight and Obesity Management: An Academy of Nutrition and Dietetics Evidence-Based Practice Guideline. Journal of Academy of Nutrition and Dietetics, 2023
Indications and Coverage of Metabolic and Bariatric Surgery: A Worldwide IFSO Survey Comparing Different National Guidelines	10.1007/s11695-024-07142-3	1. Clinical practice guidelines for the perioperative nutrition, metabolic, and nonsurgical support of patients undergoing bariatric procedures - 2019 update: cosponsored by American Association of Clinical Endocrinologists/American College of Endocrinology, The Obesity Society, American Society for Metabolic & Bariatric Surgery, Obesity Medicine Association, and American Society of Anesthesiologists, Surgery for obesity and related diseases, 2020<br>2. Clinical practice guidelines of the European Association for Endoscopic Surgery (EAES) on bariatric surgery: update 2020 endorsed by IFSO-EC, EASO and ESPCOP<br>3. Evaluation and Treatment of Obesity and Its Comorbidities: 2022 Update of Clinical Practice Guidelines for Obesity by the Korean Society for the Study of Obesity;<br>4. 2022 American Society for Metabolic and Bariatric Surgery (ASMBS) and International Federation for the Surgery of Obesity and Metabolic Disorders (IFSO) Indications for Metabolic and Bariatric Surgery
Clinical practice guidelines for older adults living with overweight and obesity: A scoping review	10.1016/j.nutos.2024.04.006	1. National clinical guidelines: The management of obesity in adults. Ministry of Public Health Qatar. 2020. Version 2.0.<br>2. Obesity National Clinical Management Guidelines, Sultanate of Oman, 2024<br>3. Mexican Clinical Practice Guidelines for Adult Overweight and Obesity Management 2024<br>4. [Obesity (children, adolescents and adults)], The Finnish Medical Society Duodecim, 2024,<br>5. American Association of Clinical Endocrinologists and American College of Endocrinology Comprehensive Clinical Practice Guidelines For Medical Care of Patients with Obesity 2016
New obesity guideline fights against stigmatization. Even family doctors have prejudices	10.1007/s15006-024-4470-6	1. [Interdisciplinary Guideline of Quality S3 on "Prevention and Therapy of Obesity"], German Obesity Society (DAG), 2024

Systematic Development of National Guidelines for Obesity Care: The Swedish Approach	10.1159/000536320	1. Regional Recommendations for the Treatment and Management of Adult Obesity<br>in the Gulf & Lebanon, World Obesity Federation and the Gulf & Lebanon Recommendations Expert Group, 20202. 2022 American Society Of Metabolic And Bariatric Surgery (ASMBS) and International Federation for the Surgery of Obesity and metabolic Disorders (IFSO) indications for metabolic and bariatric surgery, Surgery of Obesity and Related Diseases, 2022<br>3. [National guidelines for obesity care], The National Board of Health and Welfare Sweden,2023
[Clinical approach to the patient with obesity document by the Argentine Society of Medicine, the Uruguayan Society of Internal Medicine, and the International Forum of Internal Medicine]	https://pubmed.ncbi.nlm.nih.gov/39666382/	1. [Clinical approach to the patient with obesity document by the Argentine Society of Medicine, the Uruguayan Society of Internal Medicine, and the International Forum of Internal Medicine], 2024
JJS Medical Nutrition Therapy obesity guidelines updated: what do European dietitians think about their implementation?	10.1159/000535909	European Association for the Study of Obesity Position Statement on Medical Nutrition Therapy for the Management of Overweight and Obesity in Adults Developed in Collaboration with the European Federation of the Associations of Dietitians, 2023
Can a simple circuit resistance training meet aerobic and strength activities recommendations for people living with obesity?	10.1016/j.scispo.2024.06.001	N/A
Assessing the state of obesity care: Quality, access, guidelines, and standards	10.1002/osp4.765	1. Joint international consensus statement for ending stigma of obesity, Rubino, 2020
Current treatment recommendations for obesity	https://www.arzneimitteltherapie.de/heftarchiv/2024/04/aktuelle-therapieempfehlungen-der-adipositas.html	N/A
Obesity in Children and Adolescents: 2022 Update of Clinical Practice Guidelines for Obesity by the Korean Society for the Study of Obesity	10.7570/jomes23060	1. Evaluation and treatment of obesity and its comorbidities: 2022 update of clinical practice guidelines for obesity by the Korean Society for the Study of Obesity.
Maternal obesity management: a narrative literature review of health policies	10.1186/s12905-024-03342-2	N/A
Defining success in adult obesity management: A systematic review and framework synthesis of clinical practice guidelines	10.1111/cob.12631	N/A

Dissemination of the 2022 ASMBS and IFSO Guidelines for Bariatric Surgery: What Has Reached Primary Care Providers?	10.1007/s11695-024-07449-1	1. Clinical practice guidelines for the perioperative nutrition, metabolic, and nonsurgical support of patients undergoing bariatric procedures – 2019 update: cosponsored by American Association of Clinical Endocrinologists/American College of Endocrinology, The Obesity Society, American Society for Metabolic & Bariatric Surgery, Obesity Medicine Association, and American Society of Anesthesiologists, Surgery for Obesity and Related Diseases, 2020
Protocol for the guidelines for the diagnosis and treatment of obesity/overweight in infertile populations (2025)	10.3760/cma.j.cn101441-20241028-00387	N/A
The Gestational Obesity Weight Management: Implementation of National Guidelines (GLOWING) study: a pilot cluster randomised controlled trial	10.1186/s40814-024-01450-2	N/A
[Introduction to the differences in key points of obesity management between domestic and international guidelines]	10.3760/cma.j.cn112148-20240327-00172	1. AGA Clinical Practice Guideline on Pharmacological Interventions for Adults With Obesity 2022<br>2. Expert consensus on TCM diagnosis and treatment of obesity, 2022<br>3. Guideline for rational medication of obesity in primary care, Chinese Medical Association, 2021<br>4. Guidelines for medical nutrition treatment of overweight/obesity in China, Asia Pacific Journal of Clinical Nutrition, 2021<br>5. Guideline for primary care of obesity, Chinese Medical Association, 2019<br>6. Chinese Guidelines for Surgical Treatment of Obesity and Type 2 Diabetes, 2019<br>7. The 20 actions for Weight-Loss Actions for Healthy Weight Management in Chinese Residents: An Expert Consensus Based on Scientific and Evidence-based Results, 2023, The Obesity Prevention and Control Section Expert Group of the Chinese Nutrition Society<br>8. Chinese Expert Consensus on Preoperative Clinical Pathways for Case Management in Obesity and Metabolic Surgery (2023 Edition)<br>9 Chinese Expert Consensus on Precision Obesity Metabolic Surgery (2022 Edition)<br>10. Expert consensus on the weight management process in overweight or obese people, Chinese Journal of Health Management, 2021
Implementation of lifestyle medicine in healthcare: An inventory of Dutch clinical practice guidelines	10.1159/000538585	N/A

Gestational weight gain: Toward best practices in managing gestational weight gain in patients with obesity: Comparison of recommendations	10.1016/j.ejogrb.2024.05.009	N/A
Clinical practice guidelines for the management of overweight and obesity published internationally: A scoping review	10.1111/obr.13700	N/A
The latest evidence and clinical guidelines for use of meal replacements in very-low-calorie diets or low-calorie diets for the treatment of obesity	10.1111/dom.15819	1. Guidelines for Canadian bariatric surgical and medical centres: a statement from the Canadian Association of Bariatric Physicians and Surgeons, 2022
European recommendations from health care professionals and people living with obesity on safe practice for bariatric and metabolic surgery medical tourism: a modified delphi consensus statement from EASO, IFSO-EC and ECPO	10.1097/JS9.0000000000002171	1. European Recommendations from Health Care Professionals and People Living with Obesity on Safe Practice for Bariatric and Metabolic Surgery Medical Tourism: A Modified Delphi Consensus Statement from EASO, IFSO-EC and ECPO, 2024
Upcoming Italian clinical practice guidelines on endoscopic bariatric treatment of overweight and obesity: design and methodological aspects	10.1007/s13304-024-01843-1	1. American Society for Gastrointestinal Endoscopy–European Society of Gastrointestinal Endoscopy guideline on primary endoscopic bariatric and metabolic therapies for adults with obesity 2024<br>2. British Obesity Metabolic Surgery Society endorsed guidelines for psychological support pre- and postbariatric surgery, 20193. Clinical Practice Guidelines For The Perioperative Nutrition, Metabolic, and Nonsurgical Support of Patients Undergoing Bariatric Procedures – 2019 Update: Cosponsored By American Association of Clinical Endocrinologists/American College of Endocrinology, The Obesity Society, American Society For Metabolic & Bariatric Surgery, Obesity Medicine Association, and American Society of Anesthesiologists<br>4. OSSI (Obesity and Metabolic Surgery Society of India) Guidelines for Patient and Procedure Selection for Bariatric and Metabolic Surgery, 2020<br>5. British Obesity and Metabolic Surgery Society Guidelines on perioperative and postoperative biochemical monitoring and micronutrient replacement for patients undergoing bariatric surgery—2020 update<br>6. EAES rapid guideline: systematic review, network meta-analysis, CINeMA and GRADE assessment, and European consensus on bariatric surgery–extension 2022<br>7. Guidelines for Canadian bariatric surgical and medical centres: a statement from the Canadian Association of Bariatric Physicians and Surgeons, 2022<br>8. IFSO (International Federation for Surgery of Obesity and Metabolic

		Disorders) Consensus Conference Statement on OneAnastomosis Gastric Bypass (OAGB-MGB):<br>Results of a Modified Delphi Study, 2020<br>9. Very-low-calorie ketogenic diet (VLCKD) in the management of metabolic diseases: systematic review and consensus statement from the Italian Society of Endocrinology (SIE), 2019<br>10. SICOB Italian clinical practice guidelines for the surgical treatment of obesity,2024<br>11. SICOB-endorsed national Delphi consensus on obesity treatment optimization: focus on diagnosis, pre-operative management, and weight regain/insufficient weight loss approach, 2023
Scientific Evidence for the Updated Guidelines on Indications for Metabolic and Bariatric Surgery (IFSO/ASMBS)	10.1007/s11695-024-07370-7	N/A
Development of the International Federation for Surgery of Obesity and Metabolic Disorders-European Chapter (IFSO-EC) Grade-Based Guidelines on the Surgical Treatment of Obesity Using Multimodal Strategies: Design and Methodological Aspects	10.3390/jcm13175106	N/A
An international Delphi consensus on patient preparation for metabolic and bariatric surgery	10.1111/cob.12722	N/A
A Guideline-Directed Approach to Obesity Treatment	10.2337/dsi24-0001	N/A
Consensus on pharmacological treatment of obesity in Latin America	10.1111/obr.13683	N/A
Primary care for individuals with Obesity: A critical Overview and recommendations	https://cccn.ca/_uploads/663e79369419f.pdf	N/A
Guidance for the phased introduction of new medical therapies for weight management: A joint position statement by the Society for Endocrinology and Obesity Management Collaborative UK	10.1159/000538577	N/A
Erratum to: Definition, criteria, and core concepts of guidelines for the management of obesity disease in Japan	10.1507/endocrj.EJ71-6_AuthorCorrection	1. Guidelines for the Management of Obesity Disease, Japan Society for the Study of Obesity, 2022
A Closer Look at ACC/AHA and ESCÂ Guidelines for Managing Obesity and Overweight in Adults	10.1016/j.jacadv.2023.100570	N/A

ADA issues new screening, obesity management recommendations	https://www.medscape.com/viewarticle/ada-issues-new-screening-obesity-management-recommendations-2023a1000v2p	N/A
Summarizing Consensus Guidelines on Obesity Management: A Joint, Multidisciplinary Venture of the International Federation for the Surgery of Obesity & Metabolic Disorders (IFSO) and World Gastroenterology Organisation (WGO)	10.1097/MCG.0000000000001916	1. ASMBS position statement on weight bias and stigma, 2019<br>2. Clinical practice guidelines for the perioperative nutrition, metabolic, and nonsurgical support of patients undergoing bariatric procedures – 2019 update: cosponsored by American Association of Clinical Endocrinologists/American College of Endocrinology, The Obesity Society, American Society for Metabolic & Bariatric Surgery, Obesity Medicine Association, and American Society of Anesthesiologists, Obesity, 2019
Samenvatting van het Kinderdeel van de 'Richtlijn overgewicht en obesitas bij volwassenen en kinderen. Diagnostiek, ondersteuning en zorg voor mensen met obesitas of overgewicht in combinatie met risicofactoren en/of comorbiditeit'	10.1007/s12508-023-00387-w	N/A
Review of an international pilot project to adapt the Canadian Adult Obesity Clinical Practice Guideline	10.1016/j.obpill.2023.100090	1. Obesity in adults: clinical practice guide adapted for Chile 2022
Improving access and evaluation for body contouring surgery in massive weight loss patients with unified, public guidelines	10.1016/j.bjps.2023.10.085	N/A
Beyond the BMI: a Critical Analysis of the Edmonton Obesity Staging System and the New Guidelines for Indications for Metabolic and Bariatric Surgery	10.1007/s11695-023-06516-3	N/A
Implementing evidence-based obesity management guidelines requires development of medical competencies: A commentary outlining future directions in obesity education in Canada	10.1016/j.obpill.2023.100086	N/A
Managing obesity: A need to bring guidelines to practice	10.1016/j.mjafi.2022.11.005	N/A
How are adolescents engaged in obesity and chronic disease prevention policy and guideline development? A scoping review	10.1186/s41256-023-00294-2	N/A

Obesity: current state of the problem, multidisciplinary approach. (based on the consensus of the World Gastroenterological Organization Obesity 2023and the European guideline on obesity care in patients with gastrointestinal and liver diseases, 2022)	10.31146/1682-8658-ecg-218-10-5-47	N/A
How do guideline recommended energy targets compare with measured energy expenditure in critically ill adults with obesity: A systematic literature review	10.1016/j.clnu.2023.02.003	N/A
Methodology and Results of a Joint IFSO-WGO Delphi Survey of 94 Intercontinental, Interdisciplinary Experts in Obesity Management	10.1007/s11695-023-06757-2	1. IFSO-WGO Guidelines on Obesity 2022
Achieving equity in bariatric and metabolic surgery: why do we need new guidelines?	10.1111/ans.18767	1. 2022 American Society of Metabolic and Bariatric Surgery (ASMBS) and International Federation for the Surgery of Obesity and Metabolic Disorders (IFSO) indications for metabolic and bariatric surgery, Obesity. Surgery. 2023
Zinc and Copper Deficiency in Bariatric Patients: Time to Revise Guidelines?	10.1093/bjs/znad258.202	N/A
Approved Anti-Obesity Medications in 2022 KSSO Guidelines and the Promise of Phase 3 Clinical Trials: Anti-Obesity Drugs in the Sky and on the Horizon	10.7570/jomes23032	N/A
[Consensus paper of the Austrian Obesity Society on the diagnosis and treatment of people with obesity]	10.1007/s00508-023-02276-3	1. [Consensus paper of the Austrian Obesity Society on the diagnosis and treatment of people with obesity], 2023
Hidradenitis suppurativa should be recognized as a weight-related comorbidity in National Institute for Health and Care guidelines on managing obesity	10.1093/ced/llad109	1. Semaglutide for managing overweight and obesity [TA875], NICE, 2023
European Association for the Study of Obesity Position Statement on Medical Nutrition Therapy for the Management of Overweight and Obesity in Adults Developed in Collaboration with the European Federation of the Associations of Dietitians	10.1159/000528083	1. European Association for the Study of Obesity Position Statement on Medical Nutrition Therapy for the Management of Overweight and Obesity in Adults Developed in Collaboration with the European Federation of the Associations of Dietitians, 2023<br>2. European Guidelines for Obesity Management in Adults with a Very Low-Calorie Ketogenic Diet: A Systematic Review and Meta-Analysis, 2021
Are Current Guidelines Perpetuating Weight Stigma? A Weight-Skeptical Approach to the Care of Patients with Obesity	10.1007/s11606-022-07821-w	N/A

Diagnosis of Obesity: 2022 Update of Clinical Practice Guidelines for Obesity by the Korean Society for the Study of Obesity	10.7570/jomes23031	N/A
Clinical pathways in the management of the obese: Pre- and postoperative aspects	10.1016/j.jviscsurg.2022.12.009	1. Guidelines for perioperative care in bariatric surgery: enhanced recovery after surgery (ERAS) Society recommendations: a 2021 update<br>2. Nutrition, Physical Activity, and Prescription of Supplements in Pre- and Post-bariatric Surgery Patients: a Practical Guideline, Obesity Surgery, 2019
Obesity in Pregnancy: A Comprehensive Review of Influential Guidelines	10.1097/OGX.0000000000001091	N/A
Moving beyond eat less, move more using willpower: Reframing obesity as a chronic disease impact of the 2020 Canadian obesity guidelines reframed narrative on perceptions of self and the patientâ€“provider relationship	10.1111/cob.12615	N/A
Reply to Letter to the Editor: Beyond the BMI: a Critical Analysis of the Edmonton Obesity Staging System and the New Guidelines for Indications for Metabolic and Bariatric Surgery	10.1007/s11695-023-06517-2	N/A
Overweight and obesity in children and adolescents: what care pathway in 2023?	10.1016/S0985-0562(24)00011-6	N/A
Therapy of obesity. Review of selected guidelines	10.15557/PiMR.2023.0027	1. [Clinical recommendations for the management of patients with obesity 2022. PTLO position] Polish Philosophical Society.2022)
Primary care physicians' knowledge and attitudes about obesity, adherence to treatment guidelines and association with confidence to treat obesity: a Swedish survey study	10.1159/000530456	N/A
Practical guideline on obesity care in patients with gastrointestinal and liver diseases â€“ Joint ESPEN/UEG guideline	10.1016/j.clnu.2023.03.021	1. Endocrine and Nutritional Management of the Post-Bariatric Surgery Patient: An Endocrine Society Clinical Practice Guideline , 2010 (retired 2021)
How will the latest modifications to the CG189 NICE guidelines for obesity management likely impact clinical care?	10.21037/hbsn-23-143	N/A
Addressing Obesity and Dental Caries: Rationale and Practical Guidelines, a Narrative Review	10.4103/jss.jss_264_22	N/A
New Obesity Guidelines Submitted by Bariatric Surgeon Dr. Shashank Shah	link.gale.com/apps/doc/A778096562/HRCA?u=anon~d1a	1. ESI Clinical Practice Guidelines for the Evaluation and Management of Obesity In India, Endocrine Society of India, 2022

	b407c&sid=sitemap&xid=cca3ebc	
Review and evaluation of vancomycin dosing guidelines for obese individuals	10.1080/17425255.2022.2098106	N/A
[Patient care pathway before bariatric surgery]	https://pubmed.ncbi.nlm.nih.gov/35289523/	N/A
Adherence to Treatment Recommendations in Chronic Disease: What Is (Im)Possible? Expert Conclusions from the 30th ECOG Workshop 2021	10.1159/000526406	N/A
Guidelines for treating child and adolescent obesity: A systematic review	10.3389/fnut.2022.902865	N/A
Differences Between the 2016 and 2022 Editions of the Enhanced Recovery After Bariatric Surgery (ERABS) Guidelines: Call to Action of FAIR Data and the Creation of a Global Consortium of Bariatric Care and Research	10.1007/s11695-022-06132-7	N/A
New Horizons. A New Paradigm for Treating to Target with Second-Generation Obesity Medications	10.1210/clinem/dgab848	N/A
Addressing the Urgent Need in Obesity Care and Management: A Consensus in South and Southeast Asia	10.1111/obr.13503	N/A
Pregnancy after bariatric surgery: A comparison of antenatal care practices to the 2019 international consensus recommendations in Queensland, Australia	10.1111/obr.13502	N/A
A Quality Improvement Initiative Using EPIC Smartsets to Improve Adherence to AAP Guidelines for Obesity Management at a Primary Care Pediatrics Clinic	https://publications.aap.org/pediatrics/article/149/1	N/A
Reporting of weight loss outcomes in bariatric surgery following introduction of 2015 ASMBS guidelines	10.1016/j.soard.2022.06.296	N/A
International Adaptation of the Canadian Adult Obesity Clinical Practice Guidelines	10.1002/oby.23626	N/A
Guideline compliance in bariatric surgery: a French nationwide study	10.1016/j.soard.2021.12.024	N/A

Gestational weight gain below instead of within the guidelines per class of maternal obesity: a systematic review and meta-analysis of obstetrical and neonatal outcomes	10.1016/j.ajogmf.2022.100682	N/A
20 years of obesity treatment: New guidelines, semaglutide among the biggest advances	https://www.healio.com/news/endocrinology/20220314/20-years-of-obesity-treatment-new-guidelines-semaglutide-among-the-biggest-advances	N/A
Intervention in obese patients in primary care and adherence to international guidelines	10.14704/NQ.2022.20.13.NQ88020	N/A
The Australian Obesity Management Algorithm: A simple tool to guide the management of obesity in primary care	10.1016/j.orcp.2022.08.003	N/A
Measurement of obesity in primary care practice: Chronic conditions matter	10.1093/fampra/cmab170	N/A
An Audit of Compliance With NICE Guideline: Obesity: Identification, Assessment and Intervention by the Forensic Community Mental Health Team Based at a Supported Accommodation	10.1192/bjo.2022.492	N/A
Peripartum care of persons with obesity: a scoping review of recommendations and practical tools for implementation	10.1136/bmjopen-2022-061430	N/A
Changes of Guidelines in the Management of Obese Patients With Diabetes in the Metabolic Surgery Perspective	10.17476/jmbs.2022.11.2.13	N/A
Gestational Weight Gain in Patients With Obesity and Diabetes: A Comparison of 2009 Institute of Medicine Versus 2019 JAMA Recommendations	https://journals.lww.com/greenjournal/Abstract/2022/05001/Gestational_Weight_Gain_in_Patients_With_Obesity.267.aspx	N/A
Thrombotic and hemorrhagic risk in bariatric surgery with multimodal rehabilitation programs comparing 2 reduced guidelines for pharmacological prophylaxis	10.1016/j.cireng.2021.03.020	N/A

Obesity Surgery	https://pubmed.ncbi.nlm.nih.gov/34819222/	N/A
Are we 'being, having or living with' overweight?: Combating stigma with the development of communication guidelines on obesity for a public health organisation	10.1111/obr.13503	N/A
Current status of obesity treatment in Korea: based on the 2020 Korean Society for the Study of Obesity guidelines for obesity management	10.5124/jkma.2022.65.7.388	N/A
Patient body composition is largely ignored in evidence informing hypertension guidelines in pregnancy	10.1016/j.ajog.2021.11.693	N/A
Development of the Italian Clinical Practice Guidelines on Bariatric and Metabolic Surgery: Design and Methodological Aspects	10.3390/nu15010189	N/A
A review of current guidelines for the treatment of obesity	10.37765/ajmc.2022.89292	N/A
Comprehensive Understanding for Application in Korean Patients with Type 2 Diabetes Mellitus of the Consensus Statement on Carbohydrate-Restricted Diets by Korean Diabetes Association, Korean Society for the Study of Obesity, and Korean Society of Hyperte	10.4093/dmj.2022.0051	N/A
Improving compliance with venous thromboembolism prophylaxis guidelines in obese inpatients	10.1111/imj.15899	N/A
Peripartum care and obesity: A scoping review of evidence-based recommendations and practical modalities for implementation	10.1016/j.ajog.2021.11.1150	N/A
Primary care physicians' knowledge and attitudes about obesity, adherence to treatment guidelines and association with confidence to treat obesity: a Swedish survey study	10.1186/s12875-022-01811-x	N/A
Adaptation of the Canadian Adult Obesity Clinical Practice Guidelines for Ireland	10.1159/000524649	N/A

Drivers of medicalization in the Canadian Adult Obesity Clinical Practice Guidelines	10.17269/s41997-022-00662-4	N/A
A cross-sectional survey: The "Saudi guidelines for the prevention and management of obesity": Awareness and adaptation in Al-Qassim province	10.4103/jfmpc.jfmpc_631_22	N/A
Bariatric Tourism is Safe and Effective When Performed in a High-Volume Center (19801 patients) Following ASMBS Guidelines	10.1016/j.soard.2022.06.241	N/A
[Procedural guideline for laparoscopic one anastomosis gastric bypass (OAGB) (2022 edition)]	10.3760/cma.j.cn441530-20220718-00318	1. Guidelines for Standardized Surgery for Laparoscopic one anastomosis gastric bypass (OAGB) (2022 Edition), (2022 edition), Chinese Society for Metabolic and Bariatric Surgery(CSMBS), 2022
WHO discussion paper: draft recommendations for the prevention and management of obesity over the life course, including potential targets.	https://www.who.int/publications/m/item/who-discussion-paper-draft-recommendations-for-the-prevention-and-management-of-obesity-over-the-life-course-including-potential-targets	N/A
Interventions and guidelines for promoting maternal and child health in the interconception period: a literature and policy review	10.1016/S0140-6736(21)02631-3	N/A
Barriers and facilitators to implement national guidelines about weight management in primary care: A quantitative study using TDF	10.1159/000516203	N/A
No Uniformity in the References of Clinical Practice Guidelines for Bariatric Surgery: a Review of 3 Similar Guidelines Published in 2020	10.1007/s11695-021-05746-7	N/A
AGA Technical Review on Intragastric Balloons in the Management of Obesity	10.1053/j.gastro.2021.02.043	N/A
Spotlight: Intragastric Balloons in the Management of Obesity	10.1053/j.gastro.2021.03.004	N/A
Does post-bariatric surgery nutritional follow-up in primary care in the UK meet current clinical guidance?	10.1159/000515911	N/A

A Global Survey by the International Federation for the Surgery of Obesity and Metabolic Disorders (IFSO) on Perceptions of Bariatric Medical Tourism (BMT) by Health Professionals: Guidelines from IFSO for BMT	10.1007/s11695-020-05185-w	N/A
Treatment of overweight and obesity during and after a pandemic. Let's not wait for the development of complications - New guidelines for doctors	10.12740/PP/130768	N/A
European Guidelines for Obesity Management in Adults with a Very Low-Calorie Ketogenic Diet: A Systematic Review and Meta-Analysis	10.1159/000515381	N/A
Itâ€™s changed my life!' Evaluation and improvement of a pilot Tier 2 weight management course, 'Balance'	10.1108/MHRJ-07-2019-0025	N/A
Synopsis of the 2020 U.S. VA/DoD Clinical Practice Guideline for the Management of Adult Overweight and Obesity	10.1093/milmed/usab114	N/A
Weight management across preconception, pregnancy, and postpartum: A systematic review and quality appraisal of international clinical practice guidelines	10.1111/obr.13310	N/A
Micronutrient status in bariatric surgery patients receiving postoperative supplementation per guidelines: Insights from a systematic review and meta-analysis of longitudinal studies	10.1111/obr.13249	N/A
Endobariatric procedures for obesity: clinical indications and available options	10.1177/2631774520984627	N/A
Thrombotic and hemorrhagic risk in bariatric surgery with multimodal rehabilitation programs comparing 2 reduced guidelines for pharmacological prophylaxis	10.1016/j.ciresp.2020.11.008	N/A
Formulation and interpretation of the Chinese Guidelines for Surgical Treatment of Obesity and Type 2 Diabetes Mellitus	10.5582/bst.2021.01287	N/A
A critical review of obesity in healthcare systems in brazil and portugal: Pathways, guidelines and strategies	10.2174/1874944502114010206	N/A

High-Intensity Interval Training in Metabolic Diseases	10.1249/fit.0000000000000703	N/A
Toolkit Offers Guidelines to Surgery Centers Treating Obese Patients	https://www.reliasmedia.com/articles/147674-toolkit-offers-guidelines-to-surgery-centers-treating-obese-patients	N/A
Announcement of an updated Belgian consensus on the assessment and management of obesity	10.1080/17843286.2020.1727665	N/A
Correction to: Nutrition, Physical Activity, and Prescription of Supplements in Pre- and Post-bariatric Surgery Patients: a Practical Guideline (Obesity Surgery, (2019),	10.1007/s11695-019-04323-3	1. BASO consensus on the assessment and management of overweight and obesity, Belgian Association for the Study of Obesity, 2020
Anti-obesity drug therapy in clinical practice: Evidence of a poor prescriptive attitude	10.1016/j.biopha.2020.110320	N/A
Direct Oral Anticoagulants in Obesity: An Updated Literature Review	10.1177/1060028020923584	N/A
Implementation of a clinical practice guideline in a primary care setting for the prevention and management of obesity in adults	10.1111/nuf.12453	N/A
"There's always something else": Patient perspectives on improving the implementation of obesity guidelines in general practice	10.1016/j.orcp.2020.09.004	N/A
U.s. va and dod clinical practice guideline for the management of overweight and obesity: In brief	10.1002/oby.23063	N/A
Monitoring gestational weight gain and prepregnancy BMI using the 2009 IOM guidelines in the global population: a systematic review and meta-analysis	10.1186/s12884-020-03335-7	N/A
Weight Loss Interventions as Treatment of Obesity Hypoventilation Syndrome. A Systematic Review	10.1513/AnnalsATS.201907-554OC	N/A
Value-Based Healthcare and Enhanced Recovery After Surgery Implementation in a High-Volume Bariatric Center in Italy	10.1007/s11695-020-04464-w	N/A

Association of the National Health Guidance Intervention for Obesity and Cardiovascular Risks With Health Outcomes Among Japanese Men	10.1001/jamainternmed.2020.4334	N/A
The American Academy of Pediatrics hypertension guidelines identify obese youth at high cardiovascular risk among individuals non-hypertensive by the European Society of Hypertension guidelines	10.1177/2047487319868326	N/A
The ZEPmax-Program-Effective, Guideline-Based Multimodal Treatment of Obesity and Related Diseases	10.1055/a-1181-8065	N/A
Prevalent bariatric dietary practices: Is India on the same page?	10.4103/jmas.JMAS_205_19	N/A
Switching the focus from weight to health: Canada's adult obesity practice guideline set a new standard for obesity management	10.1016/j.eclinm.2020.100636	N/A
A narrative review on the clinical practice guidelines for the management of overweight/obesity in adults	10.22159/ajpcr.2020.v13i2.36404	N/A
Dosage of Anticoagulants in Obesity: Recommendations Based on a Systematic Review	10.1055/s-0040-1718405	N/A
Correction to Guideline No. 392-Pregnancy and Maternal Obesity Part 2: Team Planning for Delivery and Postpartum Care (Journal of Obstetrics and Gynaecology Canada (2019)	10.1016/j.jogc.2020.01.011	N/A
Clinical practice guideline for the diagnosis and treatment of pediatric obesity: recommendations from the Committee on Pediatric Obesity of the Korean Society of Pediatric Gastroenterology Hepatology and Nutrition	10.3345/kjp.2018.07360	N/A
[Understanding the China Blue Paper on Obesity Prevention and Control and policy implications and recommendations for obesity prevention and control in China]	10.3760/cma.j.issn.0253-9624.2019.09.003	1. [Guidelines for the prevention and control of overweight and obesity in Chinese adults (Trial)], 2012
Obesity in pregnancy: a comparison of four national guidelines	10.1080/14767058.2018.1440546	N/A

Nutrition, Physical Activity, and Prescription of Supplements in Pre- and Post-bariatric Surgery Patients: a Practical Guideline	10.1007/s11695-019-04112-y	N/A
Caring for pregnant women with obesity: Implications of policy on practice...Australian College of Midwives National Conference, 17-19 September 2019, National Convention Centre Canberra, Canberra, Australia	10.1016/j.wombi.2019.07.219	N/A
Erratum: 2018 Korean society for the study of obesity guideline for the management of obesity in Korea (Journal of Obesity & Metabolic Syndrome (2019) 28 (40-45) DOI: 10.7570/jomes.2019.28.1.40)	10.7570/JOMES.2019.28.2.143	N/A
Corrigendum: 2018 Korean Society for the Study of Obesity Guideline for the Management of Obesity in Korea	10.7570/jomes.2019.28.2.143	N/A
Management of overweight and obesity in primary care-A systematic overview of international evidence-based guidelines	10.1111/obr.12889	N/A
Bariatric surgery for type 2 diabetics-are we following recommendations? Service improvement audit in a district general hospital, UK	10.1007/s11695-019-04101-1	N/A
Cost consequences of non-compliance with GLP-1 analogues treatment guidelines in France	10.1007/s00125-019-4946-6	1. Care pathway guide: overweight and obesity in adults, Haute Autorite de Sante (HAS) France, 2024<br>2. [Adult obesity: 2nd and 3rd level management], HAS, 2024
Making space for weight bias: The process of including weight bias in Canadian clinical practice guidelines for the management of obesity in adults	10.1159/000489691	N/A
"90% of the time, it's not just weight": General practitioner and practice staff perspectives regarding the barriers and enablers to obesity guideline implementation	10.1016/j.orcp.2019.04.001	N/A
[Recommendations for monitoring nutritional deficiencies after bariatric surgery]	https://pubmed.ncbi.nlm.nih.gov/30892841/	N/A
Canadian consensus conference statement: Enhanced recovery after surgery (ERAS) in bariatric surgery	10.1007/s00464-019-06703-3	N/A

Care pathways in obesity in France: A subject in effervescence, but brakes to lift!	10.1016/j.cnd.2019.03.004	N/A

Supplementary table 5. Reasons for full text exclusion of reports identified through database and platform searches.

Systematic review of national and international obesity guidelines reasons for full text exclusion for reports identified through database and platform searches		
**Title**	**DOI/URL**	**Reason**
SAGES guidelines for the management of comorbidities relevant to metabolic and bariatric surgery	10.1007/s00464-024-11433-2	Describe the management of obesity as part of a guideline on another health condition , or solely in a specific subpopulation
Expert consensus on digestive endoscopic treatment for obesity in China	10.3760/cma.j.cn321463-20231130-00501	Not published in English
Gastro-esophageal diagnostic workup before bariatric surgery or endoscopic treatment for obesity: position statement of the International Society of Diseases of the Esophagus	10.1093/dote/doae006	Does not provide recommendations relating to at least one of the specified areas of interest
Praktické klinické doporučení pro perioperační péči v bariatrické chirurgii [Practical Clinical Recommendations for Perioperative Care in Bariatric Surgery 2023]:	10.33699/PIS.2023.102.7.283-297	Not published in English
Definition, criteria, and core concepts of guidelines for the management of obesity disease in Japan	10.1507/endocrj.EJ23-0593	Not published in English
Changing the global obesity narrative to recognize and reduce weight stigma: A position statement from the World Obesity Federation	10.1111/obr.13642	Does not clearly state the systematic approach and evidence base that underpins the guideline recommendations
Current recommendations for procedure selection in class I and II obesity developed by an expert modified Delphi consensus	10.1038/s41598-024-54141-6	Does not clearly state the systematic approach and evidence base that underpins the guideline recommendations
Guidelines for obesity clinic consultations in primary healthcare clinics	10.5124/jkma.2024.67.4.240	Not published in English;
American Society for Metabolic and Bariatric Surgery position statement on one-anastomosis gastric bypass	10.1016/j.soard.2023.11.003	Does not clearly state the systematic approach and evidence base that underpins the guideline recommendations
Recommendations on dietary treatment of obesity in adults: 2024 position of the Polish Society of Dietetics	10.5114/jhi.2024.141201	Does not clearly state the systematic approach and evidence base that underpins the guideline recommendations
SICOB Italian clinical practice guidelines for the surgical treatment of obesity and associated diseases	10.1007/s13304-024-01996-z	Does not clearly state the systematic approach and evidence base that underpins the guideline recommendations

using GRADE methodology on bariatric and metabolic surgery		
Scientific evidence for the updated guidelines on indications for metabolic and bariatric surgery (IFSO/ASMBS)	10.1016/j.soard.2024.05.009	Does not clearly state the systematic approach and evidence base that underpins the guideline recommendations
International consensus position statement on the role of obesity management medications in the context of metabolic bariatric surgery: expert guideline by the International Federation for the Surgery of Obesity and Metabolic Disorders (IFSO)	10.1093/bjs/znae283	Does not clearly state the systematic approach and evidence base that underpins the guideline recommendations
Italian guidelines for the management of adult individuals with overweight and obesity and metabolic comorbidities that are resistant to behavioral treatment	10.1007/s40618-024-02361-y	Describe the management of obesity as part of a guideline on another health condition , or solely in a specific subpopulation
MGB-OAGB International Club-Results of a Modified Delphi Consensus on Controversies in OAGB	10.1007/s11695-024-07563-0	Does not clearly state the systematic approach and evidence base that underpins the guideline recommendations
Ketogenic nutritional therapy (KeNuT)â€"a multi-step dietary model with meal replacements for the management of obesity and its related metabolic disorders: a consensus statement from the working group of the Club of the Italian Society of Endocrinology (SI	10.1007/s40618-023-02258-2	Does not clearly state the systematic approach and evidence base that underpins the guideline recommendations
Guideline for chronic weight management and clinical practice of anti-obesity medications(2024 version)	10.3760/cma.j.cn311282-20240412-00149	Not published in English
Expert consensus on glucagon-like peptide-1 receptor agonist analogs combined with lifestyle interventions for weight loss (2024 edition)	10.3760/cma.j.cn115791-20240816-00498	Not published in English
SICOB-endorsed national Delphi consensus on obesity treatment optimization: focus on diagnosis, pre-operative management, and weight regain/insufficient weight loss approach	10.1007/s40519-023-01537-4	Does not clearly state the systematic approach and evidence base that underpins the guideline recommendations
Peri-operative management of obese patients in digestive surgery: Clinical practice guidelines from the French Society of Digestive Surgery	10.1016/j.jviscsurg.2023.03.005	Does not clearly state the systematic approach and evidence base that underpins the guideline recommendations

Obesity in South and Southeast Asia-A new consensus on care and management	10.1111/obr.13520	Does not clearly state the systematic approach and evidence base that underpins the guideline recommendations
Nutrition, Physical Activity, and Prescription of Supplements in Pre- and Post-bariatric Surgery Patients: An Updated Comprehensive Practical Guideline	10.1007/s11695-023-06703-2	Does not clearly state the systematic approach and evidence base that underpins the guideline recommendations
PRACTICAL CLINICAL RECOMMENDATIONS FOR PERIOPERATIVE CARE IN BARIATRIC SURGERY 2023: ADAPTION OF ERAS (ENHANCED RECOVERY AFTER SURGERY) RECOMMENDATIONS WITH CONSENSUAL VOTING OF WORKING GROUP OF JOINT SECTION OF BARIATRIC AND METABOLIC SURGERY OF CZECH SU	10.33699/PIS2023.102.7.283-297	Not published in English
Practical clinical recommendations for perioperative care in bariatric surgery 2023	10.48095/ccgh2023509	Not published in English
Clinical practice guidelines for perioperative care in bariatric surgery 2023: Adapted ERAS (enhanced recovery after surgery) guidelines with consensual voting of the working group of the Joint Bariatric and Metabolic Surgery Section of the Czech Surgery Society	10.33699/PIS.2023.102.7.283-297	Not published in English
Overweight and obesity in adults: 14 key messages to improve practices 2023	10.1016/S0985-0562(24)00013-X	Not published in English
Recommendations for the prevention and management of obesity in the Iraqi population	10.1080/00325481.2023.2172914	Does not clearly state the systematic approach and evidence base that underpins the guideline recommendations
An analysis of enhanced recovery pathways for bariatric surgery -preoperative fasting, carbohydrate loading, and aspiration risk: a position statement from the International Society for the Perioperative Care of Patients with Obesity	10.1016/j.soard.2022.12.030	Does not clearly state the systematic approach and evidence base that underpins the guideline recommendations
Obesity: identification, assessment and management []CG 189, NICE 2023	https://www.nice.org.uk/guidance/cg189	Have been superseded by a more recent guideline
American Association of Clinical Endocrinology Consensus Statement: Addressing Stigma and Bias in the Diagnosis and Management of Patients with	10.1016/j.eprac.2023.03.272	Does not clearly state the systematic approach and evidence base that underpins the guideline recommendations

Obesity/Adiposity-Based Chronic Disease and Assessing Bias and Stigmatization as Determinants of Disease Severi		
Evaluation and Treatment of Obesity and Its Comorbidities: 2022 Update of Clinical Practice Guidelines for Obesity by the Korean Society for the Study of Obesity	10.7570/jomes23016	Does not clearly state the systematic approach and evidence base that underpins the guideline recommendations
Best practice approach for redo-surgeries after sleeve gastrectomy, an expert's modified Delphi consensus	10.1007/s00464-023-09879-x	Does not clearly state the systematic approach and evidence base that underpins the guideline recommendations
Achieving consensus on the language of obesity: a modified Delphi study	10.1016/j.eclinm.2023.102061	Does not include a rating of the quality of evidence that underpins the recommendations
Consensus on Current Landscape and Treatment Trends of Obesity in India for Primary Care Physicians	10.59556/japi.71.0349	Does not clearly state the systematic approach and evidence base that underpins the guideline recommendations
What to remember from the HAS 2022 best clinical practice recommendations: Obesity in adults: “2nd and 3rd levels of care management – Part I: medical management”?	10.1016/S0985-0562(24)00012-8	Not published in English
Managing the gastrointestinal side effects of GLP-1 receptor agonists in obesity: recommendations for clinical practice	10.1080/00325481.2021.2002616	Does not clearly state the systematic approach and evidence base that underpins the guideline recommendations
ESI Clinical Practice Guidelines for the Evaluation and Management of Obesity In India	10.4103/2230-8210.356236	Does not clearly state the systematic approach and evidence base that underpins the guideline recommendations
Obesity in adults: Clinical practice guideline adapted for Chile	10.5867/medwave.2022.10.2649	Does not clearly state the systematic approach and evidence base that underpins the guideline recommendations
Enhanced recovery after bariatric surgery: an Italian consensus statement	10.1007/s00464-022-09498-y	Does not clearly state the systematic approach and evidence base that underpins the guideline recommendations
Chinese expert consensus on the prevention and treatment of postoperative nausea and vomiting after laparoscopic sleeve gastrectomy (2022 edition)	10.3760/cma.j.cn115610-20220823-00467	Not published in English
Obesity clinical practice guideline	http://www.jasso.or.jp/contents/magazine/journal.html	Not published in English

Medical care of obesity in adults: Recommendations from the High Health Authority and the French Federation of Nutrition	10.1016/j.cnd.2022.07.003	Not published in English
Clinical Recommendations to Manage Gastrointestinal Adverse Events in Patients Treated with Glp-1 Receptor Agonists: A Multidisciplinary Expert Consensus	10.3390/jcm12010145	Does not clearly state the systematic approach and evidence base that underpins the guideline recommendations
Guidelines for Canadian bariatric surgical and medical centres: a statement from the Canadian Association of Bariatric Physicians and Surgeons	10.1503/cjs.020719	Does not clearly state the systematic approach and evidence base that underpins the guideline recommendations
2022 American Society for Metabolic and Bariatric Surgery (ASMBS) and International Federation for the Surgery of Obesity and Metabolic Disorders (IFSO): Indications for Metabolic and Bariatric Surgery	10.1016/j.soard.2022.08.013	Does not clearly state the systematic approach and evidence base that underpins the guideline recommendations
Operative management of acute abdomen after bariatric surgery in the emergency setting: the OBA guidelines	10.1186/s13017-022-00452-w	Does not clearly state the systematic approach and evidence base that underpins the guideline recommendations
Chinese expert consensus on precision obesity metabolic surgery (2022 edition)	10.3760/cma.j.cn115610-20220915-00523	Not published in English
EAES rapid guideline: systematic review, network meta-analysis, CINeMA and GRADE assessment, and European consensus on bariatric surgery-extension 2022	10.1007/s00464-022-09008-0	Describe the management of obesity as part of a guideline on another health condition , or solely in a specific subpopulation
Updating obesity management strategies: An audit of Italian specialists	10.1007/s40519-022-01402-w	Does not clearly state the systematic approach and evidence base that underpins the guideline recommendations
Obesity in Adults: A 2022 Adapted Clinical Practice Guideline for Ireland	10.1159/000527131	Does not clearly state the systematic approach and evidence base that underpins the guideline recommendations
Expert consensus on obesity prevention and treatment in China	10.7652/jdyxb202204023	Not published in English
[Expert Consensus on Obesity Prevention and Treatment in China]	10.3760/cma.j.cn112338-20220402-00253	Not published in English
Guidelines for medical nutrition treatment of overweight/obesity in China (2021)	10.6133/apjcn.202209_31(3).0013	Does not clearly state the systematic approach and evidence base that underpins the guideline recommendations

[Chinese expert consensus on precision obesity metabolic surgery (2022 edition)]	10.3760/cma.j.cn441530-20220717-00315	Not published in English; Barrie Tyner (2025-02-07 22:03:12)(Select): Full text not available in English - only the abstract
Recommendations for nutritional care after bariatric surgery: Recommendations for best practice and SOFFCO-MM/AFERO/SFNCM/expert consensus	10.1016/j.jviscsurg.2020.10.013	Does not clearly state the systematic approach and evidence base that underpins the guideline recommendations
Exercise training in the management of overweight and obesity in adults: Synthesis of the evidence and recommendations from the European Association for the Study of Obesity Physical Activity Working Group	10.1111/obr.13273	Does not clearly state the systematic approach and evidence base that underpins the guideline recommendations
Brazilian Consensus on Endoscopic Sleeve Gastroplasty	10.1007/s11695-020-04915-4	Does not clearly state the systematic approach and evidence base that underpins the guideline recommendations
The first modified Delphi consensus statement on sleeve gastrectomy	10.1007/s00464-020-08216-w	Does not clearly state the systematic approach and evidence base that underpins the guideline recommendations
2020 Korean Society for the Study of Obesity Guidelines for the Management of Obesity in Korea	10.7570/jomes21022	Does not clearly state the systematic approach and evidence base that underpins the guideline recommendations
Multidisciplinary clinical consensus on diagnosis and treatment of obesity (2021 edition)	10.3760/cma.j.cn115610-20210829-00422	Not published in English
Patient and procedure selection for bariatric and metabolic surgery in Malaysia- The Malaysian Consensus	https://pubmed.ncbi.nlm.nih.gov/33742633/	Does not clearly state the systematic approach and evidence base that underpins the guideline recommendations
Obesity in adults: a clinical practice guideline	10.1503/cmaj.191707	Does not clearly state the systematic approach and evidence base that underpins the guideline recommendations
Thai society for metabolic and bariatric surgery consensus guideline on bariatric surgery for the treatment of obese patient in Thailand	https://mahidol.elsevierpure.com/en/publications/thai-society-for-metabolic-and-bariatric-surgery-consensus-guidel	Does not include a rating of the quality of evidence that underpins the recommendations
How to fight the obesity stigma: An international consensus	10.53126/MEB39437	Does not clearly state the systematic approach and evidence base that underpins the guideline recommendations
Metabolic and Bariatric Surgery Chapter of the Association of Polish Surgeons. Bariatric and metabolic surgery care standards	10.5114/wiitm.2020.97935	Does not clearly state the systematic approach and evidence base that underpins the guideline recommendations

Joint international consensus statement for ending stigma of obesity	10.1038/s41591-020-0803-x	Does not clearly state the systematic approach and evidence base that underpins the guideline recommendations
European Society of Endocrinology Clinical Practice Guideline: Endocrine work-up in obesity	10.1530/eje-19-0893	Does not clearly state the systematic approach and evidence base that underpins the guideline recommendations
Clinical Practice Guidelines for the Perioperative Nutrition, Metabolic, and Nonsurgical Support of Patients Undergoing Bariatric Procedures â€" 2019 Update: Cosponsored by American Association of Clinical Endocrinologists/American College of Endocrinology,	10.1002/oby.22719	Does not clearly state the systematic approach and evidence base that underpins the guideline recommendations
ISPRM/ESPRM guidelines on Physical and Rehabilitation Medicine professional practice for adults with obesity and related comorbidities	10.23736/S1973-9087.20.06232-2	Describe the management of obesity as part of a guideline on another health condition , or solely in a specific subpopulation
Main prophylactic measures in bariatric endoscopy. Spanish Expert Recommendations Guideline	10.17235/reed.2020.6970/2020	Does not clearly state the systematic approach and evidence base that underpins the guideline recommendations
Canadian consensus statement: enhanced recovery after surgery in bariatric surgery	10.1007/s00464-019-06911-x	Does not clearly state the systematic approach and evidence base that underpins the guideline recommendations
OSSI (Obesity and Metabolic Surgery Society of India) Guidelines for Patient and Procedure Selection for Bariatric and Metabolic Surgery	10.1007/s11695-020-04497-1	Does not clearly state the systematic approach and evidence base that underpins the guideline recommendations
The importance of language in engagement between health-care professionals and people living with obesity: a joint consensus statement	10.1016/S2213-8587(20)30102-9	Does not clearly state the systematic approach and evidence base that underpins the guideline recommendations
A Spanish Society joint SECO and SEEDO approach to the Post-operative management of the patients undergoing surgery for obesity	10.1007/s11695-019-04043-8	Does not clearly state the systematic approach and evidence base that underpins the guideline recommendations
2018 Korean Society for the Study of Obesity Guideline for the Management of Obesity in Korea	10.7570/jomes.2019.28.1.40	Does not clearly state the systematic approach and evidence base that underpins the guideline recommendations
British Obesity Metabolic Surgery Society endorsed guidelines for psychological support pre- and post-bariatric surgery	10.1111/cob.12339	Does not clearly state the systematic approach and evidence base that underpins the guideline recommendations

Duodenal switch in revisional bariatric surgery: conclusions from an expert consensus panel	10.1016/j.soard.2019.03.009	Does not clearly state the systematic approach and evidence base that underpins the guideline recommendations
Clinical Practice Guidelines For The Perioperative Nutrition, Metabolic, and Nonsurgical Support of Patients Undergoing Bariatric Procedures â€“ 2019 Update: Cosponsored By American Association of Clinical Endocrinologists/American College of Endocrinology	10.4158/gl-2019-0406	Does not clearly state the systematic approach and evidence base that underpins the guideline recommendations
Reoperative surgery for nonresponders and complicated sleeve gastrectomy operations in patients with severe obesity. An international expert panel consensus statement to define best practice guidelines	10.1016/j.soard.2018.11.006	Does not clearly state the systematic approach and evidence base that underpins the guideline recommendations
Spanish consensus document on bariatric endoscopy. Part 2: specific endoscopic treatments	10.17235/reed.2019.4922/2017	Does not clearly state the systematic approach and evidence base that underpins the guideline recommendations
European Practical and Patient-Centred Guidelines for Adult Obesity Management in Primary Care	10.1159/000496183	Does not clearly state the systematic approach and evidence base that underpins the guideline recommendations
ASMBS updated position statement on bariatric surgery in class I obesity (BMI 30-35 kg/m(2))	10.1016/j.soard.2018.05.025	Published prior to 2019
Position of the Academy of Nutrition and Dietetics: Interventions for the Treatment of Overweight and Obesity in Adults	https://doi.org/10.1016/j.jand.2015.10.031	Published prior to 2019
American Association of Clinical Endocrinologists and American College of Endocrinology Comprehensive Clinical Practice Guidelines For Medical Care of Patients with Obesity	https://doi.org/10.4158/EP161365.GL	Published prior to 2019
European Guidelines for Obesity Management in Adults	10.1159/000442721	Published prior to 2019
Pharmacological Management of Obesity: An Endocrine Society Clinical Practice Guideline	10.1210/jc.2014-3415	Published prior to 2019

Supplementary table 6. Collated grey literature records for full text screening and screening results.

Name of full text	Outcome	Final reason for exclusion (if applicable)
Overweight and obesity management NICE guideline [NG246], 2025	Include	N/A
National Clinical Guidelines :The Diagnosis & Management of Obesity in Adults, Ministry of Public Health Qatar, 2024	Exclude	Does not clearly state the systematic approach and evidence base that underpins the guideline recommendations
National Clinical Guidelines: Metabolic And Bariatric Surgery In Adults, Ministry of Public Health Qatar, 2025	Exclude	Does not clearly state the systematic approach and evidence base that underpins the guideline recommendations
European Association for the Study of Obesity Position Statement on Medical Nutrition Therapy for the Management of Overweight and Obesity in Adults Developed in Collaboration with the European Federation of the Associations of Dietitians 2023	Exclude	Does not clearly state the systematic approach and evidence base that underpins the guideline recommendations
[National guidelines for obesity care], The National Board of Health and Welfare Sweden,2023	Exclude	Not published in English
Overgewicht en obesitas bij volwassenen en kinderen [Overweight and obesity in adults and children], Dutch Association of Medical Specialists, Netherlands 2023	Exclude	Not published in English
[Obesity (children, adolescents and adults)], Finland, 2024	Exclude	Not published in English
Therapy of overweight and obesity resistant to behavioral treatment in the adult population with metabolic comorbidities Associazione Medici Endocrinologi (AME), Italy, 2023	Exclude	Not published in English
[Clinical guidelines on the treatment of adults individuals with obesity], Directorate of Health, Iceland, 2020	Exclude	Not published in English
[Non-surgical treatment of severe obesity], Dansk Endokrinologisk Selskab, Denmark, 2023	Exclude	Not published in English
[Obesity Diagnosis and Treatment Guide], Türkiye Endokrinoloji ve Metabolizma Derneği , Turkey, 2019	Exclude	Not published in English
[Adult obesity: 2nd and 3rd level support], Haute Autorité de santé, France, 2022	Exclude	Not published in English
BASO consensus on the assessment and management of overweight and obesity, Belgian Association for the Study of Obesity, 2020	Exclude	Not published in English
[Surgical treatment of obesity], Dutch Association of Medical Specialists, Netherlands 2020	Exclude	Not published in English
Obesity in Adults: Position Statement of Polish Association for the Study on Obesity, Polish Association of Endocrinology, Polish Association of Cardiodiabetology, Polish Psychiatric Association, Section of Metabolic and Bariatric Surgery of the Association of Polish Surgeons, and the College of Family Physicians in Poland, 2023	Exclude	Does not clearly state the systematic approach and evidence base that underpins the guideline recommendations
[Guidelines for prevention and the fight against overweight and obesity], Ministero della Salute, Italy, 2019	Exclude	Not published in English
[Standard diagnostic and therapeutic procedure for the complex management of overweight/obesity in adulthood],Slovakia, 2023	Exclude	Not published in English

Implementation of Obesity Science Into Clinical Practice: A Scientific Statement From the American Heart Association, 2024	Exclude	Does not provide recommendations relating to at least one of the specified areas of interest
Tirzepatide for managing overweight and obesity, NICE, 2024	Exclude	Does not clearly state the systematic approach and evidence base that underpins the guideline recommendations
Semaglutide for managing overweight and obesity, NICE, 2023	Exclude	Does not clearly state the systematic approach and evidence base that underpins the guideline recommendations
Liraglutide for managing overweight and obesity, NICE, 2020	Exclude	Does not clearly state the systematic approach and evidence base that underpins the guideline recommendations
The H2020 “NoHoW Project”: A Position Statement on Behavioural Approaches to Longer-Term Weight Management, 2021	Exclude	Does not clearly state the systematic approach and evidence base that underpins the guideline recommendations
Digital technologies for delivering multidisciplinary weight-management services: early value assessment, NICE, 2024	Exclude	Does not clearly state the systematic approach and evidence base that underpins the guideline recommendations
Swallowable gastric balloon capsule for weight loss, NICE, 2020	Exclude	Does not clearly state the systematic approach and evidence base that underpins the guideline recommendations
American society for metabolic and bariatric surgery: intra-operative care pathway for minimally invasive Roux-en-Y gastric bypass, 2024	Exclude	Does not clearly state the systematic approach and evidence base that underpins the guideline recommendations
Guidelines for Perioperative Care in Bariatric Surgery: Enhanced Recovery After Surgery (ERAS) Society Recommendations: A 2021 Update	Exclude	Does not clearly state the systematic approach and evidence base that underpins the guideline recommendations
IFSO Bariatric Endoscopy Committee Evidence-Based Review and Position Statement on Endoscopic Sleeve Gastroplasty for Obesity Management, 2024	Exclude	Does not include recommendations for adults (minimum aged ≥18 years)
IFSO- WGO Guideline Obesity, 2023	Exclude	Does not clearly state the systematic approach and evidence base that underpins the guideline recommendations
Endoscopic sleeve gastroplasty for obesity, NICE, 2024	Exclude	Does not clearly state the systematic approach and evidence base that underpins the guideline recommendations
Society for Endocrinology guidelines for the diagnosis and management of post-bariatric hypoglycaemia, 2024	Exclude	Describe the management of obesity as part of a guideline on another health condition , or solely in a specific subpopulation
Metabolic bone changes after bariatric surgery: 2020 update, American Society for Metabolic and Bariatric Surgery Clinical Issues Committee position statement	Exclude	Does not clearly state the systematic approach and evidence base that underpins the guideline recommendations
American Society for Metabolic and Bariatric Surgery updated statement on single-anastomosis duodenal switch 2020	Exclude	Does not clearly state the systematic approach and evidence base that underpins the guideline recommendations
[Guideline for the prevention and treatment of obesity], German Obesity Society (DAG), Germany, 2024	Exclude	Not published in English
Clinical Practice Guideline for the Management of Overweight and Obesity in Adults in Saudi Arabia, 2022	Exclude	Does not clearly state the systematic approach and evidence base that underpins the guideline recommendations
The Clinical Practice Guidelines (CPG) for the Management of Obesity, Malaysia, 2023	Exclude	Does not clearly state the systematic approach and evidence base that underpins the guideline recommendations

Clinical Practice Guideline of Korean Medicine for Obesity , 2024	Exclude	Not published in English
Regional Recommendations for the Treatment and Management of Adult Obesity in the Gulf & Lebanon, World Obesity Federation and the Gulf & Lebanon Recommendations Expert Group, 2020	Exclude	Does not clearly state the systematic approach and evidence base that underpins the guideline recommendations
Consensus On Definitions And Clinical Practice Guidelines For Patients Considering Metabolic-Bariatric Surgery, 2023	Exclude	Does not clearly state the systematic approach and evidence base that underpins the guideline recommendations
European recommendations from health care professionals and people living with obesity on safe practice for bariatric and metabolic surgery medical tourism: a modified delphi consensus statement from EASO, IFSO-EC and ECPO, 2024	Exclude	Does not clearly state the systematic approach and evidence base that underpins the guideline recommendations
Methodology and Results of a Joint IFSO-WGO Delphi Survey of 94 Intercontinental, Interdisciplinary Experts in Obesity Management, 2023	Exclude	Does not clearly state the systematic approach and evidence base that underpins the guideline recommendations
[Clinical Protocol and Therapeutic Guidelines (PCDT) for overweight and obesity in adults], Brazil Ministry of Health, 2022	Exclude	Not published in English
[Manual of Care for Overweight and Obese People in the Scope of Primary Health Care (PHC) of the SUS], Brazil Ministry of Health, 2023	Exclude	Not published in English
Obesity National Clinical Management Guidelines, Sultanate of Oman, 2024	Exclude	Does not clearly state the systematic approach and evidence base that underpins the guideline recommendations
[Clinical approach to the patient with obesity document by the Argentine Society of Medicine, the Uruguayan Society of Internal Medicine, and the International Forum of Internal Medicine], 2024	Exclude	Not published in English
Expert consensus on TCM diagnosis and treatment of obesity, 2022	Exclude	Not published in English
Guideline for rational medication of obesity in primary care, Chinese Medical Association, 2021	Exclude	Not published in English
Guidelines for primary diagnosis and treatment of obesity, Chinese Medical Association, 2019	Exclude	Not published in English
Chinese Guidelines for Surgical Treatment of Obesity and Type 2 Diabetes, 2019	Exclude	Not published in English
Chinese expert consensus on the weight management process in overweight or obese people, 2021	Exclude	Not published in English
Chinese Expert Consensus on Preoperative Clinical Pathways for Case Management in Obesity and Metabolic Surgery (2023 Edition)	Exclude	Not published in English
Chinese Expert Consensus on Precision Obesity Metabolic Surgery (2022 Edition)	Exclude	Not published in English
Guidelines for the Management of Obesity Disease, Japan Society for the Study of Obesity, 2022	Exclude	Not published in English
ASMBS position statement on weight bias and stigma, 2019	Exclude	Does not clearly state the systematic approach and evidence base that underpins the guideline recommendations
[Consensus paper of the Austrian Obesity Society on the diagnosis and treatment of people with obesity], 2023	Exclude	Not published in English

Nutrition, physical activity, and prescription of supplements in pre- and post-bariatric surgery patients: a practical guideline, 2019	Exclude	Are at a regional, local, or non-national healthcare organisation level
[Clinical recommendations for the management of patients with obesity 2022. PTLO position] Polish Philosophical Society.2022	Exclude	Not published in English
Endocrine and Nutritional Management of the Post-Bariatric Surgery Patient: An Endocrine Society Clinical Practice Guideline, 2010	Exclude	Published prior to 2019
Guidelines for Standardized Surgery for Laparoscopic one anastomosis gastric bypass (OAGB) (2022 Edition), (2022 edition), Chinese Society for Metabolic and Bariatric Surgery(CSMBS), 2022	Exclude	Not published in English
Care pathway guide: overweight and obesity in adults, Haute Autorite de Sante (HAS) France, 2024	Exclude	Does not clearly state the systematic approach and evidence base that underpins the guideline recommendations
ASMBS position statement on preoperative patient optimization before metabolic and bariatric surgery, 2021	Exclude	Does not clearly state the systematic approach and evidence base that underpins the guideline recommendations
American Society of Metabolic and Bariatric Surgery consensus statement on laparoscopic adjustable gastric band management, 2022	Exclude	Does not clearly state the systematic approach and evidence base that underpins the guideline recommendations
ASMBS Bariatric Emergencies for the General Surgeon Statement, 2023	Exclude	Does not clearly state the systematic approach and evidence base that underpins the guideline recommendations
AGA Clinical Practice Update on Evaluation and Management of Early Complications After Bariatric/Metabolic Surgery: Expert Review, 2021	Exclude	Does not clearly state the systematic approach and evidence base that underpins the guideline recommendations
ASMBS Position Statement on the Relationship Between Obesity and Cancer and the Role of Bariatric Surgery, 2020	Exclude	Does not clearly state the systematic approach and evidence base that underpins the guideline recommendations
Postoperative nausea and vomiting in bariatric surgery: a position statement endorsed by the ASMBS and the ISPCOP, 2021	Exclude	Does not clearly state the systematic approach and evidence base that underpins the guideline recommendations
ASMBS Preoperative Care Pathway for Laparoscopic Roux-en-Y Gastric Bypass, American Society for Metabolic and Bariatric Surgery, 2021	Exclude	Does not clearly state the systematic approach and evidence base that underpins the guideline recommendations
Behavioral Weight Loss Interventions to Prevent Obesity-Related Morbidity and Mortality in Adults: Recommendation Statement, U.S. Preventive Services Task Force, 2019	Exclude	Does not clearly state the systematic approach and evidence base that underpins the guideline recommendations
Evidence-based Guideline on Adult Obesity Prevention and Management, Taiwan, 2023	Exclude	Not published in English

Supplementary table 7. Characteristics of included guidelines.

Guideline title	Country	First published	Last updated	Population(s)	Overall setting(s)	Topics within scope covered in the guideline
Overweight and obesity management NICE guideline [NG246]	England and Wales	January 2025	February 2025: minor update to wording	Children, young people and adults living with overweight or obesity	All settings where publicly funded health and social care services provided	Weight bias; Medical nutrition therapy; Physical activity; Psychological and behavioural interventions; Pharmacotherapy; Bariatric surgery; Primary care and primary healthcare; Emerging technologies and virtual medicine
Mexican Clinical Practice Guidelines for Adult Overweight and Obesity Management	Mexico	October 2024	No update	Adults living with overweight and obesity	Interdisciplinary healthcare settings	Medical nutrition therapy; Physical activity; Psychological and behavioural interventions; Pharmacotherapy; Primary care and primary healthcare
American Society for Gastrointestinal Endoscopy (ASGE)-European Society of Gastrointestinal Endoscopy (ESGE) guideline on primary endoscopic bariatric and metabolic therapies for adults with obesity	US and Europe	April 2024	No update	Adults with obesity	Healthcare settings	Bariatric surgery; *Medical nutrition therapy*; Physical activity*; Psychological and behavioural interventions**<br>**As part of lifestyle modification interventions in conjunction with bariatric surgery or metabolic therapy*
Medical Nutrition Therapy Interventions Provided by Dietitians for Adult Overweight and Obesity Management: An Academy of Nutrition and Dietetics Evidence-Based Practice Guideline	US	March 2023	No update	Adults with overweight or obesity	Medical Nutrition Therapy intervention settings	Medical nutrition therapy; *Weight bias*; Physical activity*; Psychological and behavioural interventions*; Primary care and primary healthcare*; Emerging technologies and virtual medicine**<br>**In the context of medical nutrition therapy*
American Gastroenterological Association Clinical Practice Guideline on Pharmacological Interventions for Adults With Obesity	US	November 2022	No update	Adults living with overweight and obesity	Healthcare settings	Pharmacological; *Medical nutrition therapy*; Physical activity*; Psychological and behavioural interventions**<br>**As part of lifestyle modification interventions in conjunction with pharmacotherapy*
Effect of Carbohydrate-Restricted Diets and Intermittent Fasting on Obesity, Type 2 Diabetes Mellitus, and Hypertension Management: Consensus Statement of the Korean Society for the Study of Obesity,	South Korea	June 2022	No update	Adults living with overweight or obesity	Healthcare settings	Medical nutrition therapy

Guideline title	Country	First published	Last updated	Population(s)	Overall setting(s)	Topics within scope covered in the guideline
Korean Diabetes Association, and Korean Society of Hypertension						
American Gastroenterological Association Clinical Practice Guidelines on Intragastric Balloons in the Management of Obesity	US	April 2021	No update	Adults with BMI >25 kg/m^2 seeking an intervention for weight loss	Not reported	Bariatric surgery; *Medical nutrition therapy*; Physical activity*; Psychological and behavioural interventions** **As part of lifestyle modification intervention in conjunction with intragastric balloon therapy*
British Obesity and Metabolic Surgery Society Guidelines on Perioperative and Postoperative Biochemical Monitoring and Micronutrient Replacement for Patients Undergoing Bariatric Surgery-2020 Update	UK	November 2020	No update	Adults undergoing: adjustable gastric band, sleeve gastrectomy, Rouxen-Y gastric bypass and biliopancreatic diversion/duodenal switch	Surgical settings	Bariatric surgery; *medical nutrition therapy*; Psychological and behavioural interventions** **In the context of specialist postoperative dietetic support including support and guidance to achieve long-term weight loss and weight maintenance*
Veterans Association /Department of Defense Clinical Practice Guideline for the Management of Adult Overweight and Obesity	US	July 2020	No update	Adults living with overweight and obesity	Veterans Association /Department of Defense healthcare settings	Medical nutrition therapy; Physical activity; Psychological and behavioural interventions; Pharmacotherapy; Bariatric surgery; Emerging technologies and virtual medicine
Clinical practice guidelines of the European Association for Endoscopic Surgery (EAES) on bariatric surgery: update 2020 endorsed by IFSO-EC, EASO and ESPCOP	Europe	April 2020	No update	Adults living with obesity	Surgical settings	Bariatric surgery

Supplementary table 8. Reported search dates and key clinical questions within scope for included guidelines.

Guideline title	Reported search dates and key clinical/research questions within scope
Overweight and obesity management NICE guideline [NG246]	Search dates<br>RQ 1.3 and 1.4 Clinical effectiveness: 1st January 2000 to 13th April 2023, cost-effectiveness: 1st January 2000 to 1st February 2022<br>The searches were limited from 2000 for systematic reviews, RCTs and observational studies and limited from 2010 for qualitative studies.<br>RQ 2.1 Clinical effectiveness: 1st January 2006 to 14th April 2023, cost-effectiveness: 1st January 2006 to 4th March 2022<br>RQ 2.2 Clinical effectiveness: 1st November 2013 - 19th January 2022, cost-effectiveness: from inception to 3rd February 2022<br>The clinical effectiveness search was designed as an update of the Colquitt Cochrane review from 2014, which was based on searches done in November 2013.<br>RQ 2.5 Clinical effectiveness: 1st January 2007 - 14th April 2023, cost-effectiveness: 1st January 2007 - 12th April 2022<br><br>Key clinical questions within scope<br>1.3 What approaches are effective and cost-effective in increasing uptake of weight management services in adults?<br>1.4 What are the barriers and facilitators to increasing uptake of weight management services in adults?<br>2.1 What is the effectiveness and cost effectiveness of total or partial meal replacements, intermittent fasting, plant-based and low carbohydrate diets, in achieving and maintaining weight loss in adults living with overweight or obesity?<br>2.2 What referral criteria for bariatric surgery are most effective to achieve weight loss and maintain a healthier weight in adults living with obesity?<br>2.5 What is the effectiveness, cost-effectiveness and acceptability of psychological approaches to address the counterproductive effect of weight stigma in achieving or maintaining weight loss, or negating the adverse impact of weight stigma, in children, young people, and adults?
Mexican Clinical Practice Guidelines for Adult Overweight and Obesity Management	Search date<br>29th May 2024<br>Key clinical questions within scope<br>**Clinical evaluation**<br>**Interdisciplinary obesity care**<br>1. In adults 18 years of age or older with Class II obesity with or without comorbidities, should they be referred to a multidisciplinary program versus conventional treatment?<br>**Treatment and follow up**<br>**Medical nutrition therapy**<br>2. In adults 18 years of age or older with overweight or obesity, should a dietary plan be prescribed by a nutritionist compared to a physician at the first point of contact?<br>3. In Mexican adults 18 years of age or older with overweight or obesity, should the Milpa Diet (type of traditional Mexican diet) be used compared to a balanced hypoenergetic diet?<br>**Physical activity**<br>4. In adults 18 years of age or older with overweight or obesity, should interventions aimed at controlling sedentary behaviors be used versus not using them?

	5. In adults 18 years of age or older with overweight or obesity, should exercise snacks be used compared to not using them?<br>**Behavioural interventions and mental health**<br>6, In adults 18 years of age or older with overweight or obesity, should mental health screening be performed in primary care compared to not screening?<br>7. In adults 18 years of age or older with overweight or obesity, should behavioral change tools be used versus not using them?<br>8. In adults 18 years of age or older with overweight or obesity, should group psychological interventions be used compared to individualized psychological interventions?<br>**Pharmacotherapy**<br>9. In adults 18 years of age or older with overweight or obesity, should phentermine be used for six months or longer compared to lifestyle/placebo interventions?<br>10. In adults 18 years of age or older with overweight or obesity, should fenproporex be used for six months or longer compared to lifestyle/placebo interventions?<br>11. In adults 18 years of age or older with overweight or obesity, should mazindol be used for six months or longer compared to lifestyle/placebo interventions?<br>12. In adults 18 years of age or older with overweight or obesity, should amfepramone be used for six months or longer compared to life-style/placebo interventions?<br>13. In adults 18 years of age or older with overweight or obesity, should clobenzorex be used for six months or longer compared to lifestyle/placebo interventions?<br>14. In adults 18 years of age or older with overweight or obesity without diabetes, should glucagon-like peptide 1 (GLP-1) agonists (liraglutide, semaglutide) be used compared to lifestyle/placebo interventions?<br>15. In adults 18 years of age or older with overweight or obesity without diabetes, should naltrexone/bupropion be used compared to lifestyle/placebo interventions?<br>16. In adults aged 18 years or older with obesity (BMI ≥ 30 kg/m^2), should pharmacotherapy for obesity be phenotyped compared to conventional prescribing?<br>17. In adults 18 years of age or older with overweight or obesity, should combination obesity drug therapy (liraglutide, semaglutide, naltrex-one/bupropion) be used compared to obesity drug monotherapy?<br>18. In adults 18 years of age or older with overweight or obesity without impaired glucose metabolism, should oral hypoglycemic agents (Na +-glucose cotransporter 2 [iSGLT2] inhibitors or metformin) be used compared to lifestyle interventions?
American Society for Gastrointestinal Endoscopy (ASGE)-European Society of Gastrointestinal Endoscopy (ESGE) guideline on primary endoscopic bariatric	Search dates<br>Endoscopic bariatric and metabolic therapies: 1st August 2022<br>Periprocedural care: 1990 to 15th December 2022<br>Key clinical questions within scope<br>1. Population: Adults with body mass indices of 27-29.9 kg/m^2 with at least 1 obesity-related comorbidity or body mass indices ≥30 kg/m^2.<br>Intervention: Endoscopic bariatric and metabolic therapy + lifestyle modification, Comparator: Lifestyle modification.<br>Outcomes: % total weight loss (TWL), HbA1c reduction, serious adverse event rate<br>2. Population: Adults with obesit.

and metabolic therapies for adults with obesity	Intervention: Intragastric balloon + lifestyle modification.<br>Comparator: Lifestyle modification, Outcome : %TWL, serious adverse events rate<br>3. Population: Adults with obesity undergoing intragastric balloon placement (IGB) Intervention: Antiemetics.<br>Comparator: No antiemetics,<br>Outcome: Incidence of postoperative nausea and vomiting (PONV), Rate of emergency department (ED) visit within 30 days for PONV<br>4. Population: Adults with obesity undergoing intragastric balloon placement, Intervention: Pain medications, Comparator: No pain medications. Outcome: Rate of ED visit within 30 days for pain management<br>5. Population: Adults with obesity undergoing intragastric balloon placement. Intervention: proton pump inhibitors (PPIs). Comparator: No PPIs, Outcome: Rate of gastric ulcer, Rate of bleeding serious adverse events<br>6. Popualtion: Adults with obesity, Intervention: Endoscopic gastric remodeling + lifestyle modification, Comparator: Lifestyle modification, Outcome: %TWL, serious adverse events rate<br>7. Population: Adults with obesity undergoing endoscopic gastric remodeling, Intervention: Antiemetics. Comparator: No antiemetics, Outcome: Incidence of PONV, Rate of ED visit within 30 days for PONV<br>8. Population: Adults with obesity undergoing endoscopic gastric remodeling, Intervention: Pain, Comparator: medications, Outcome: No pain medications, Rate of ED visit within 30 days for pain management<br>9. Population: Adults with obesity undergoing endoscopic gastric remodeling (EGR), Intervention: Prophylactic antibiotics, Comparator: No prophylactic antibiotics, Outcome: Rate of gastric ulcer, Rate of bleeding serious adverse events<br>10. Population: Adults with obesity undergoing endoscopic gastric remodeling, Intervention: PPIs, Comparator: No PPIs, Outcome: Rate of postprocedure infection<br>11. Population: Adults with obesity. Intervention: Aspiration therapy + lifestyle modification, Comparator: Lifestyle modification, Outcome: %TWL, serious adverse events rate<br>12. Population: Adults with obesity. Intervention:Transpyloric shuttle + lifestyle modification, Comparator: Lifestyle modification, Outcome: %TWL, serious adverse events rate<br>13. Population: Adults with obesity, Intervention: Duodenal–jejunal bypass liner + lifestyle modification. Comparator: Lifestyle modification, Outcome: HbA1c reduction, %TWL, serious adverse events rate<br>14 Population: Adults with obesity, Intervention: Duodenal mucosal resurfacing + lifestyle modification, Comparator: Lifestyle modification, Outcome: HbA1c reduction, serious adverse events rate
Medical Nutrition Therapy Interventions Provided by Dietitians for Adult Overweight and Obesity Management: An Academy of Nutrition and Dietetics	<u>Search dates</u><br>1st January 2008 to 25th January 2021<br><u>Research question</u><br>In adults with overweight or obesity, what is the effect of weight management interventions provided by a dietitian (or international equivalent), compared with usual care or no intervention from a dietitian, on body mass index, percent weight loss, waist circumference, blood pressure, fasting blood glucose, quality of life, cost-effectiveness, and adverse events?

Evidence-Based Practice Guideline	
American Gastroenterological Association Clinical Practice Guideline on Pharmacological Interventions for Adults With Obesity	<u>Search date</u><br>1st January 2022<br><u>Research question</u><br>**Problem**<br>Adults with BMI ≥ 30 kg/m^2, or ≥ 27 kg/m^2 and weight-related complications, who have had an inadequate response to lifestyle interventions<br>**Intervention**<br>Food and Drug Administration (FDA)-approved medications to treat obesity<br>(semaglutide 2.4 mg, liraglutide 3.0 mg, phentermine-topiramate extended-release (ER), naltrexone-bupropion ER, orlistat, phentermine, diethylpropion, and Gelesis100 oral superabsorbent hydrogel)<br>**Comparison**<br>Lifestyle interventions alone<br>**Outcome**<br>Benefits:<br>- % Total Body Weight loss (% TBWL)<br>- ≥ 5% Total Body Weight loss<br>- ≥10% Total Body Weight loss<br>Harms:<br>- Treatment discontinuation due to adverse events<br>- Serious Adverse Events<br>Minimal important difference<br>Smallest change in the outcome that would be identified as clinically important was set at -3% TBWL between groups
Effect of Carbohydrate-Restricted Diets and Intermittent Fasting on Obesity, Type 2 Diabetes Mellitus, and Hypertension Management: Consensus Statement of the Korean Society for the Study of Obesity, Korean	<u>Search dates</u><br>1st January 2010 to 8th June 2021<br><u>Key clinical questions within scope</u><br>1. Are carbohydrate-restricted diets helpful for weight reduction in patients living with overweight or obesity?<br>2. Are intermittent fasting helpful for weight reduction in patients living with overweight or obesity?

Diabetes Association, and Korean Society of Hypertension	
American Gastroenterological Association Clinical Practice Guidelines on Intragastric Balloons in the Management of Obesity	<u>Search dates</u><br>Database inception to September 2019, with update for new randomised control trials in January 2020.<br>Relevant exclusion criteria: evaluated balloons before 1990 or evaluated balloons placed for fewer than 6 months)<br><u>Key clinical questions within scope</u><br>1.1: In individuals with obesity seeking a weight-loss intervention, should intragastric balloon (IGB) therapy with lifestyle modification vs lifestyle modification alone be used for improvement of patient important outcomes?<br>1.2: In this population, individuals who undergo IGB therapy, should fluid-filled balloons vs gas filled balloons be used?<br>2: In individuals with obesity undergoing IGB therapy, should moderate- to high-intensity concomitant lifestyle modification vs concomitant, standard of care (SOC) lifestyle modification be used for improvement of patient important outcomes?<br>3: In individuals undergoing IGB therapy, should concomitant proton pump inhibitors (PPIs) vs no proton pump inhibitors be used for improvement of patient important outcomes?<br>4: In individuals who have undergone IGB therapy, should antiemetic therapy vs no antiemetic therapy be used for improvement of patient important outcomes?<br>5: In individuals undergoing IGB therapy, should perioperative laboratory screen for nutritional deficiencies vs no screening be performed?<br>6: In individuals who have undergone IGB therapy, should multivitamin prophylaxis vs no prophylaxis be given?<br>7: In patients who have undergone IGB therapy, should concomitant or subsequent therapy vs standard of care lifestyle modification be used for improvement of patient important outcomes?
British Obesity and Metabolic Surgery Society Guidelines on perioperative and postoperative biochemical monitoring and micronutrient replacement for patients undergoing bariatric surgery—2020 update	<u>Search dates</u><br>2014 - January 2018<br><u>Key clinical questions within scope</u><br>What are the clinical recommendations that are appropriate for:<br>• Preoperative nutritional screening/or monitoring before bariatric surgery;<br>• Postoperative nutritional screening and/or monitoring after bariatric surgery;<br>• Vitamin and mineral supplementation to prevent nutritional deficiencies following bariatric surgery;<br>• Treatment of nutritional deficiencies before/after bariatric surgery?
Veterans Association (VA)/Department of Defense (DoD) Clinical Practice Guideline for	<u>Search dates</u><br>1st February 2013 to 8th April 2019<br><u>Key clinical questions within scope</u>

the Management of Adult Overweight and Obesity	1. What are the benefits and harms of comprehensive lifestyle interventions on weight loss and health outcomes?<br>2. Among adults who have achieved initial weight loss, what are the benefits and harms of comprehensive lifestyle interventions on weight maintenance and health outcomes?<br>3. What is the comparative effectiveness of different modes of delivering comprehensive lifestyle interventions on weight loss or weight maintenance and health outcomes?<br>4. What is the comparative effectiveness and harms of various dietary approaches on short and long-term weight loss and health outcomes?<br>5. What are the benefits and harms of physical activity on short- and long-term weight loss and health outcomes?<br>6. What are the benefits and harms of Food and Drugs Administration (FDA) approved medications for short-term use (<6 months) or chronic use (>6 months) on weight loss and health outcomes?<br>7. What are the benefits and harms of FDA approved medications on weight maintenance and health outcomes?<br>8. What are the benefits and harms of dietary supplements or nutraceuticals on initial weight loss and long-term weight loss?<br>9. What are the benefits and harms of bariatric surgery on short- and long-term weight loss, health outcomes, and comorbid health conditions?<br>10. What is the comparative effectiveness of different forms of bariatric surgery on short- and long-term weight loss, health outcomes, and comorbid health conditions?<br>11. What are the benefits and harms of FDA-approved weight loss devices on short- and long-term weight loss, health outcomes, and comorbid health conditions?<br>12. What is the comparative effectiveness and harms of FDA-approved weight loss devices on short- and long-term weight loss status, health outcomes, and comorbid health conditions?
Clinical practice guidelines of the European Association for Endoscopic Surgery (EAES) on bariatric surgery: update 2020 endorsed by IFSO-EC, EASO and ESPCOP	<u>Search dates</u><br>January 2005 to 1st November 2018<br><u>Research questions</u><br>**Topic 1 Indication to surgery**<br>• Should bariatric surgery vs. non-surgical management be used for weight loss?<br>**Topic 2 Preoperative workup**<br>• Should preoperative Helicobacter pylori (H. pylori) eradication vs. standard care be used for prevention of foregut symptoms in patients undergoing bariatric surgery?<br>• Should preoperative diet consultation vs. standard care be used for weight loss in patients undergoing bariatric surgery?<br>• Should assessment of pre-operative psychological conditions vs. no assessment be used in bariatric surgery prior to operation?<br>• Should preoperative esophagogastroscopy vs. no endoscopy be used for preoperative evaluation in patients undergoing bariatric surgery?<br>**Topic 3 Perioperative management**<br>• Should perioperative obstructive sleep apnea screening vs. no screening be used for prevention of respiratory complications after bariatric surgery?<br>• Should perioperative continuous positive airway pressure (CPAP) vs. no CPAP be used for prevention of respiratory complications after bariatric surgery?<br>• Should high dose pharmacological antithrombotic prophylaxis vs. standard dose antithrombotic prophylaxis be used for prevention of thromboembolism in patients undergoing bariatric surgery?

	• Should inferior vena cava filter vs. low molecular weight heparine and intermittent pneumatic compression be used for prevention of thromboembolism in patients undergoing bariatric surgery?<br>• Should an Enhanced Recovery After Surgery (ERAS) protocol vs. standard care be used for patients undergoing bariatric surgery?<br>• Should multimodal analgesia with minimal use of opioids vs. standard analgesia be used for pain control in patients after bariatric surgery?<br>**Topic 4. Bariatric procedures**<br><u>One anastomosis procedures</u><br>• Should one-anastomosis gastric bypass (OAGB) vs. gastric plication be used for weight loss?<br>• Should OAGB vs. sleeve gastrectomy be used for weight loss?<br>• Should OAGB vs. Roux-en-Y gastric bypass (RYGB) be used for weight loss?<br>• Should OAGB vs. adjustable gastric banding (AGB) be used for weight loss?<br>• Should single-anastomosis duodeno-ileal switch (SADI-S) vs. sleeve gastrectomy be used for weight loss?<br>• Should SADI-S vs. biliopancreatic diversion with duodenal switch (BPD/DS) be used for weight loss?<br>• Should SADI-S vs. RYGB be used for weight loss?<br><u>BPD</u><br>• Should BPD/DS vs. RYGB be used for weight loss?<br>• Should BPD/DS vs. sleeve gastrectomy be used for weight loss?<br><u>RYGB</u><br>• Should RYGB vs. gastric plication be used for weight loss in obese patients?<br>•Should RYGB vs. AGB be used for weight loss in obese patients?<br>• Should RYGB vs. sleeve gastrectomy be used for weight loss in obese patients?<br><u>Sleeve gastrectomy</u><br>• Should sleeve gastrectomy vs. laparscopic adjustable gastric banding (LAGB) be used for weight loss?<br>• Should sleeve gastrectomy vs. gastric plication be used for weight loss?<br>• Should sleeve gastrectomy with antral resection (2-3 cm) vs. antral preservation (>5 cm) be used for weight loss?<br>• Should sleeve gastrectomy calibrated on bougie size ≤36 Fr vs. >36 Fr be used for weight loss?<br>• Should staple line reinforcement vs. no staple line reinforcement be used in patients undergoing sleeve gastrectomy?<br>**Topic 5 Revisional surgery**<br>• Should revisional surgery vs. no revisional surgery be used for failed bariatric surgery?<br>**Topic 6 Postoperative care**<br>• Should schedule post-operative follow up vs. no post-operative follow up be used in patients undergoing bariatric surgery?<br>• Should treatment with ursodeoxycolic acid vs. no treatment with ursodeoxycolic acid be used for prevention of gallstone disease after bariatric surgery?<br>• Should nutritional (micro and/or macronutrients) supplementation vs. no supplementation be used in in patients undergoing bariatric surgery?<br>• Should proton pump inhibitor (PPI) treatment vs. no PPI treatment be used in patients undergoing bariatric surgery?<br>• Should postoperative diet vs. no postoperative diet be used in patients undergoing bariatric surgery?

	• Should delayed pregnancy vs. early pregnancy be used for prevention of fetal complications?<br>**Topic 7 Investigational procedures**<br>• Should EndoBarrier® vs. non-surgical management be used for weight loss?<br>• Should AspireAssist® vs. non-surgical management be used for weight loss?<br>• Should abiliti® vs. non-surgical management be used for weight loss?<br>• Should vBloc® vs. non-surgical management be used for weight loss?<br>• Should Pose® procedure vs. non-surgical management be used for weight loss?<br>• Should OverStitch™ vs. non-surgical management be used for weight loss?<br>• Should Duodenal Mucosal Resurfacing vs. non-surgical management be used for weight loss?

Supplementary table 9. Quality appraisal of included guidelines (AGREE II).

Reviewer 1 and 2

Title	Domain 1			Domain 2			Domain 3								Domain 4			Domain 5				Domain 6		Overall
Item	1	2	3	4	5	6	7	8	9	10	11	12	13	14	15	16	17	18	19	20	21	22	23	Overall
NICE 2025	7,7	7,7	7,7	7,7	7,7	7,7	7,7	7,7	7,6	5,4	6,5	4,5	6,5	4,4	7,7	7,7	6,7	7,6	7,7	7,7	7,7	3,2	7,7	7,7
Mexico 2024	7,7	7,7	7,7	6,7	6,7	7,7	6,7	6,7	6,6	6,6	6,5	5,6	1,1	1,1	7,6	6,7	7,7	3,2	2,1	3,2	1,1	7,7	4,5	5,5
ASGE/ ESGE 2024	5,6	7,7	7,7	7,6	4,5	1,2	7,7	6,7	7,7	5,5	6,7	6,7	4,5	1,1	7,6	7,6	7,7	6,5	1,2	1,2	1,1	1,1	7,7	5,5
AND 2023	7, 7	7, 6	7,6	7,7	6,6	7,7	7,7	7,7	7,7	3,3	3,2	4,4	5,6	1,1	7,7	7,7	7,7	3,4	2,1	5,6	1,1	7,7	3,4	5,5
AGA Pharmacological 2022	6,5	7,7	7,7	7,7	4,3	7,7	7,7	7,7	7,7	3,4	6,7	6,7	4,5	6,5	7,7	7,7	7,7	2,1	4,5	2,3	2,1	4,4	2,2	6,6
Korea 2022	6,6	7,7	7,6	1,1	1,1	1,5	7,7	5,6	6,6	4,4	6,5	6,7	1,1	1,1	7,6	7,6	3,2	1,1	2,1	1,1	1,1	6,7	4,4	3,3
AGA IGB 2021	7,7	7,7	6,5	7,7	3,4	7,6	7,7	7,7	6,6	3,4	5,6	4,5	4,5	6,7	6,5	7,6	7,7	2,1	2,1	2,1	1,1	4,4	3,3	4,4
BOMSS 2020	6, 6	4, 5	6, 7	5, 5	3, 4	7, 6	7,7	6,6	4,4	3,4	2,3	4,4	1,1	3,2	7, 7	7, 7	7, 7	2, 3	1, 1	1, 1	1, 1	5, 6	3, 4	3, 3
VA/DoD 2020	7,7	7,7	7,7	6,7	6,7	7, 7	7,7	6,7	6,6	4,4	6,6	7,7	4,3	1,1	7,6	7,7	7,7	4,4	4,5	2,2	2,1	2,1	4,5	5,5
EAES 2020	6,5	7,7	7,7	6,6	7,6	6,7	6,7	7,7	4,5	4,3	5,4	5,6	1,1	6,7	7,7	7,7	7,7	5,5	4,5	4,5	3,4	7,7	4,3	6,6

Key: AGA - American Gastroenterological Association; AND - Academy of Nutrition and Dietetics; BOMSS - British Obesity & Metabolic Surgery Society EAES - European Association for the Study of Obesity; IFSO-EC: International Federation for Surgery of Obesity and Metabolic Disorders - European Chapter; IGB - intragastric balloons; ESPCOP: European Society for the Peri-operative Care of the Obese Patient; AGA: American Gastroenterological Association; NICE: National Institute of Health and Care Excellence; VA/DoD: Veterans Affairs/Department of Defence.

Supplementary table 10. Recommendations within scope, certainty of the evidence, strength of recommendations, and evidence to decision process.

Guideline title	Recommendations within scope	Certainty of evidence	Strength of recommendations	Rating systems used for the certainty of the evidence and recommendations, and evidence to decision process
Mexican Clinical Practice Guidelines for Adult Overweight and Obesity Management	**Clinical evaluation.**<br>**Interdisciplinary obesity care**<br>1. Referring adults aged 18 years or older with Class 2 obesity, with or without comorbidities to a multidisciplinary program.<br>**Treatment and follow up**<br>**Medical nutrition therapy**<br>2. Dietary plans be prescribed by a dietitian compared to a primary care physician in adults aged 18 years or older with obesity.<br>3. Using Milpa Diet (type of traditional Mexican diet) or a balanced hypoenergetic diet in Mexican adults aged 18 years or older with overweight or obesity.<br>**Physical activity**<br>4. Using interventions aimed at controlling sedentary behaviors in adults aged 18 years or older with overweight or obesity.<br>5. Using "exercise snacks" in adults aged 18 years or older with overweight or obesity.<br>**Behavioural interventions and mental health**<br>6. Conducting mental health screening at the primary care level in adults aged 18 years or older with overweight or obesity.<br>7. Using behavioral change tools in adults aged 18 years or older with overweight or obesity.<br>8. Using group psychological interventions compared to individualized psychological interventions in adults aged 18 years or older with overweight or obesity.<br>**Pharmacotherapy**<br>9. Using phentermine for six months or more compared to lifestyle interventions/placebo in adults aged 18 years or older with overweight or obesity.<br>10. Not using fenproporex for six months or more compared to lifestyle interventions/placebo in adults aged 18 years or older with overweight or	1. Very low<br>2. Very low<br>3. Very low<br>4. Low<br>5. Very low<br>6. Very low<br>7. Very low<br>8. Very low<br>9. Very low<br>10. Very low<br>11. Very low<br>12. Very low<br>13. Very low<br>14. Moderate<br>15. Low<br>16. Very low<br>17. Very low<br>18. Low	1. Conditional<br>2. Conditional<br>3. Conditional<br>4. Conditional<br>5. Conditional<br>6. Conditional<br>7. Conditional<br>8. Conditional<br>9. Conditional<br>10. Conditional<br>11. Conditional<br>12. Conditional<br>13. Strong<br>14. Conditional<br>15. Conditional<br>16. Conditional<br>17. Conditional<br>18. Conditional	GRADE and GRADE-CERQual (Confidence in the Evidence from Reviews of Qualitative research) methodology was used to rate the evidence.<br><br>GRADE EtD framework was used to assign strength of recommendation.<br><br>An in-person workshop with three rounds of voting (defined a priori as an agreement of ≥75%) was organised in June 2024 which used the interactive EtD (iEtD) platform to assign the strength of the recommendations

	obesity.<br>11. Not using mazindol for six months or more compared to lifestyle interventions/placebo in adults aged 18 years or older with overweight or obesity.<br>12. Not using amfepramone for six months or more compared to lifestyle interventions/placebo in adults aged 18 years or older with overweight or obesity.<br>13. Not using clobenzorex for six months or more compared to lifestyle interventions/placebo in adults aged 18 years or older with overweight or obesity.<br>14. Using long-term GLP-1 agonists (liraglutide, semaglutide) compared to lifestyle interventions/placebo in adults aged 18 years or older with overweight or obesity without diabetes.<br>15. Using naltrexone/bupropion long-term compared to lifestyle interventions/placebo in adults aged 18 years or older with overweight or obesity without diabetes.<br>16. Phenotyping pharmacotherapy for obesity compared to conventional prescription in adults aged 18 years or older with obesity (BMI ≥ 30 kg/m^2).<br>17. Not using combined drug therapy for obesity (including liraglutide, semaglutide, naltrexone/bupropion, orlistat) compared to monotherapy in adults aged 18 years or older with overweight or obesity.<br>18. Not using oral antidiabetic medications (Sodium-glucose Cotransporter-2 [SGLT2] inhibitors or metformin) compared to lifestyle interventions/placebo in adults aged 18 years or older with overweight or obesity without impaired glucose metabolism.			
American Society for Gastrointestinal Endoscopy (ASGE)-European Society of Gastrointestinal Endoscopy (ESGE) guideline on	1. In adults with overweight or obesity, the ASGE–ESGE suggests the use of endoscopic bariatric and metabolic therapies plus lifestyle modification (LM) over LM alone for patients with a BMI ≥ 30 kg/m^2 with or without an obesity-related comorbidity or a BMI of 27 to 29.9 kg/m^2 with at least 1 obesity related comorbidity.<br>2. In adults with obesity, the ASGE–ESGE suggests the use of an IGB plus LM over LM alone<br>3. In adults with obesity undergoing IGB placement, the ASGE–ESGE suggests the use of antiemetics periprocedurally.<br>4. In adults with obesity undergoing IGB placement, the ASGE–ESGE suggests the use of pain medications periprocedurally.	1. Very low<br>2. Moderate<br>3.Very Low<br>4. Very low<br>5. Very low<br>6. Moderate<br>7. Very low<br>8. Very low<br>9. Very low<br>10. Very low<br>11. Low	1. Conditional<br>2. Conditional<br>3. Conditional<br>4. Conditional<br>5. Conditional<br>6. Conditional<br>7. Conditional<br>8. Conditional<br>9. Conditional<br>10.Conditional<br>11. Conditional	GRADE methodology was used to rate the evidence.<br><br>An evidence-to-decision framework was used to formulate recommendations using the GRADEpro Guideline Development Tool (https://gradepro.org).

primary endoscopic bariatric and metabolic therapies for adults with obesity	5. In adults with obesity undergoing IGB placement, the ASGE–ESGE suggests the use of proton pump inhibitors while the IGB is in place.<br>6. In adults with obesity, the ASGE–ESGE suggests treatment with EGR plus LM over LM alone.<br>7. In adults with obesity undergoing EGR, the ASGE–ESGE suggests the use of antiemetics periprocedurally.<br>8. In adults with obesity undergoing EGR, the ASGE–ESGE suggests the use of pain medications periprocedurally.<br>9. In adults with obesity undergoing EGR, the ASGE–ESGE suggests the use of prophylactic antibiotics periprocedurally.<br>10. In adults with obesity undergoing EGR, the ASGE–ESGE suggests the use of short-term proton pump inhibitors periprocedurally.<br>11. In adults with obesity, the ASGE–ESGE suggests treatment with aspiration therapy plus LM over LM alone.<br>12. In adults with obesity, the ASGE–ESGE recommends treatment with a transpyloric shuttle only in the context of a clinical trial.	12. Knowledge gap	12. No recommendation	According to the GRADE framework, recommendations are labelled as 'strong' or 'conditional' and are phrased as 'we recommend' or 'we suggest'.
Medical Nutrition Therapy Interventions Provided by Dietitians for Adult Overweight and Obesity Management: An Academy of Nutrition and Dietetics Evidence-Based Practice Guideline	**1.0. Medical Nutrition Therapy (MNT) Approach for Adult Overweight and Obesity Management**<br>1.1 It is reasonable for registered dietitian nutritionists (RDNs) or international equivalents to use the Nutrition Care Process (NCP) to provide effective, client-centered interventions based on shared decision-making and clinical judgement and individualized to each client's needs, circumstances, and goals.<br>1.2 MNT provided by RDNs or international equivalents is recommended for adults with overweight or obesity to improve cardiometabolic outcomes, quality of life (QoL), and weight outcomes, as appropriate for and desired by each client.<br>1.3 RDNs or international equivalents should collaborate with an interprofessional health care team to provide comprehensive, multicomponent care for adults with overweight or obesity, as appropriate for and desired by each client.<br>1.4 It is reasonable for RDNs or international equivalents to monitor and evaluate client outcomes and adapt goals and interventions, including those for weight maintenance, and provide resources as needed for each client.<br>1.5 It is reasonable for RDNs or international equivalents to minimize the effects of weight bias and weight stigma and its consequences by targeting client-centered goals, individualizing interventions according to complex	1.1. Consensus<br>1.2. Moderate<br>1.3. Low<br>1.4 Consensus<br>1.5. Consensus<br>2.1. Low<br>2.2 Low<br>2.3 Low<br>2.4 Low<br>2.5. Low<br>2.6. Moderate<br>2.7. Consensus<br>3.1. Low<br>3.2. Low<br>4.2. Moderate	1.1. Consensus<br>1.2. Strong<br>1.3. Strong<br>1.4 Consensus<br>1.5. Consensus<br>2.1. Weak<br>2.2 Strong<br>2.3 Strong<br>2.4 Weak<br>2.5. Weak<br>2.6. Strong<br>2.7. Consensus<br>3.1. Strong<br>3.2. Strong<br>4.2. Strong	GRADE methodology was used to rate the evidence.<br><br>GRADE's EtD framework was used to draft recommendations.<br><br>Each recommendation was assigned a grade for strength of recommendation (number 1 or 2) and strength of supporting evidence (letter A to D).<br><br>Recommendations were graded 'consensus' if evidence was not available or unclear.

	contributors of overweight and obesity, communicating using client-preferred terms, and providing an inclusive physical environment.<br>**2.0. Delivering MNT Interventions**<br>2.1 RDNs or international equivalents may provide at least 5 interactive sessions, when feasible and desired by each adult client with overweight or obesity, to achieve the greatest potential improvement in outcomes. Frequency of contacts should be tailored to each client's preferences and needs.<br>2.2 RDNs or international equivalents should provide overweight and obesity management interventions for a duration of at least 1 year to improve and optimize cardiometabolic and weight outcomes, as appropriate for and desired by each client.<br>2.3 After completion of overweight and obesity management interventions, RDNs or international equivalents should provide follow-up contacts at least every 3 months, for as long as desired by each client, to facilitate maintenance of weight loss and improved cardiometabolic outcomes.<br>2.4 RDNs or international equivalents may use telehealth, in-person contacts, or a blend of these delivery methods when providing MNT interventions to adults with overweight or obesity. Outcomes may be optimized by including in-person contacts.<br>2.5 RDNs or international equivalents may use both individual and group delivery methods when providing MNT interventions to adults with overweight or obesity, as feasible and appropriate for each client.<br>2.6 RDNs or international equivalents providing MNT interventions for adults with overweight and obesity should coordinate care in a variety of settings, including primary care/outpatient, community, and workplace settings, to access and support each client with resources in the environment that best suits individualized needs.<br>2.7 It is reasonable and necessary for RDNs or international equivalents to be aware of and use existing channels of payment for services for adults with overweight or obesity to improve client access to care.<br>**3.0. Dietary and Lifestyle Intervention Approaches**<br>3.1 RDNs or international equivalents should advise adult clients with overweight or obesity that many different dietary patterns can be individualized to support client-centered goals. Prescribed dietary approaches should achieve and maintain nutrient adequacy and be realistic for client			

	adherence. Prescribed calorie levels should be tailored based on estimated or measured needs and should be adjusted to improve weight outcomes, as appropriate for and desired by each client.<br>3.2 RDNs or international equivalents should advise the following components as part of a comprehensive adult overweight and obesity management intervention to improve cardiometabolic outcomes, QoL, and weight outcomes, as appropriate for and desired by each client:<br>- Nutritionally adequate diet with adjusted calories to improve weight outcomes or a nutritionally adequate, energy-balanced diet for weight maintenance<br>- Behavioral strategies, including self-monitoring (diet, physical activity, weight)<br>- Appropriate physical activity to meet client goals (within the RDN's scope of practice or refer to an exercise practitioner)<br>**4.0. Special Populations**<br>4.2. Adults with obesity who receive pharmacotherapy or metabolic and bariatric surgery should collaborate with RDNs or international equivalents, as part of an interprofessional health care team, to improve and maintain a healthy diet that meets nutritional needs and advances weight loss efforts to improve cardiometabolic outcomes			
AGA Clinical Practice Guideline on Pharmacological Interventions for Adults With Obesity	1. In adults with obesity or overweight with weight-related complications, who have had an inadequate response to lifestyle interventions, the AGA recommends adding pharmacological agents to lifestyle interventions over continuing lifestyle interventions alone.<br>2. In adults with obesity or overweight with weight-related complications, the AGA suggests using semaglutide 2.4 mg with lifestyle modifications, compared with lifestyle modifications alone.<br>3. In adults with obesity or overweight with weight-related complications, the AGA suggests using liraglutide 3.0 mg with lifestyle modifications, compared with lifestyle modifications alone.<br>4. In adults with obesity or overweight with weight-related complications, the AGA suggests using phentermine-topiramate ER with lifestyle modifications, compared with lifestyle modifications alone.<br>5. In adults with obesity or overweight with weight-related complications, the AGA suggests using naltrexone-bupropion ER with lifestyle modifications, compared with lifestyle modifications alone.<br>6. In adults with obesity or overweight with weight-related complications, AGA	1. Moderate<br>2. Moderate<br>3 Moderate<br>4. Moderate<br>5. Moderate<br>6. Moderate<br>7. Low<br>8. Low<br>9. Knowledge gap	1. Strong<br>2. Conditional<br>3 Conditional<br>4. Conditional<br>5. Conditional<br>6. Conditional<br>7. Conditional<br>8. Conditional<br>9. No recommendation	GRADE methodology was used to rate the evidence.<br><br>The recommendations were formulated through consensus using the evidence-to-decision framework.<br><br>Recommendations were identified as 'strong' ('we recommend') or 'conditional' ('we suggest').

	suggests against the use of orlistat.<br>7. In adults with obesity or overweight with weight-related complications, the AGA suggests using phentermine with lifestyle modifications, compared with lifestyle modifications alone.<br>8. In adults with obesity or overweight with weight-related complications, the AGA suggests using diethylpropion with lifestyle modifications, compared with lifestyle modifications alone.<br>9. In adults with BMI between 25 and 40 kg/m^2, the AGA recommends using Gelesis 100 oral superabsorbent hydrogel only in the context of a clinical trial.			
Effect of Carbohydrate-Restricted Diets and Intermittent Fasting on Obesity, Type 2 Diabetes Mellitus, and Hypertension Management: Consensus Statement of the Korean Society for the Study of Obesity, Korean Diabetes Association, and Korean Society of Hypertension	1. In adults with overweight or obesity, a moderately-low carbohydrate diet or low carbohydrate diet (mLCD) can be considered a dietary regimen for weight reduction since similar or greater effects on weight loss are observed than the generally recommended diets.<br>2. The recommendation for intermittent fasting (IF) in adults with overweight or obesity will be withheld due to the lack of long-term studies and the heterogeneity of previous studies.	1. Moderate<br>2. N/A	1. Conditional<br>2. N/A	GRADE methodology was useded to rate the evidence and assign the strength of recommendation.<br><br>Each subcommittee member participated in the joint production of each part of the guideline.<br><br>The outcome was agreed by consensus by the chair, vice-chair, and two assigned reviewers of the subcommittee
AGA Clinical Practice Guidelines on Intragastric	1. In individuals with obesity seeking a weight-loss intervention who have failed a trial of conventional weight-loss strategies, AGA suggests the use of IGB therapy with lifestyle modification over lifestyle modification alone.<br>2. In individuals with obesity undergoing IGB therapy, AGA recommends	1. Moderate<br>2. Moderate<br>3. Moderate<br>4. Low	1. Conditional<br>2. Strong<br>3. Strong<br>4. Conditional	GRADE framework was used to rate the certainty of the evidence and the GRADE EtD framework was used to

Balloons in the Management of Obesity	moderate- to high-intensity concomitant lifestyle modification interventions to maintain and augment weight loss.<br>3. In individuals undergoing IGB therapy, AGA recommends prophylaxis with PPIs.<br>4. In individuals undergoing IGB therapy, AGA suggests using the intraoperative anesthetic regimens associated with the lowest incidence of nausea along with perioperative antiemetics. AGA suggests a scheduled antiemetic regimen for 2 weeks after IGB placement.<br>5. In individuals undergoing IGB therapy, AGA suggests against perioperative laboratory screening for nutritional deficiencies.<br>6. AGA suggests daily supplementation with 1-2 adult dose multivitamins after IGB placement.<br>7. After IGB removal, AGA suggests subsequent weight-loss or maintenance interventions that include dietary interventions, pharmacotherapy, repeat IGB, or bariatric surgery. The choice of weight-loss or maintenance method after IGB is determined based on the patient's context and comorbidities following a shared decision-making approach.	5. Low<br>6. Very low<br>7. Low	5. Conditional<br>6. Conditional<br>7. Conditional	formulate the strength of the recommendations.<br><br>All recommendations were identified as strong or conditional.
British Obesity and Metabolic Surgery Society Guidelines on perioperative and postoperative biochemical monitoring and micronutrient replacement for patients undergoing bariatric surgery—2020 update	**Preoperative nutritional assessment**<br>1. All people should have a comprehensive nutritional assessment prior to bariatric surgery<br>*Haematinics*<br>2. Check full blood count including haemoglobin, ferritin, folate and vitamin B12 levels)<br>*Vitamin D, calcium and parathyroid hormone*<br>3. Check serum 25-hydroxyvitamin D levels<br>4. Check serum calcium levels<br>5. Check serum/plasma parathyroid hormone levels<br>*Thiamine*<br>6. There is insufficient evidence to support a recommendation to screen an individual's thiamine levels pre-surgery; however, some individuals may have low levels<br><br>**Postoperative care and biochemical monitoring**<br>7. Specialist postoperative dietetic support should be provided including individualized nutritional supplementation, support and guidance to achieve long-term weight loss and weight maintenance	**Preoperative nutritional assessment**<br>1. Grade D<br>2. Grade B<br>3. Grade B<br>4. Grade D<br>5. Grade B<br>6. Grade D<br>**Postoperative care and biochemical monitoring**<br>7. Grade D<br>8. Grade D<br>9. Grade D<br>10. Grade B<br>11. Grade B<br>12. Grade B	**Preoperative nutritional assessment**<br>1. Evidence Level (EL) 4<br>2. EL 2 (1+ to 4)<br>3. EL 2 (2++ to 3)<br>4. EL 4<br>5. EL 2 (2++ to 3)<br>6. EL 3 (2− to 3)<br>**Postoperative care and biochemical monitoring**<br>7. EL 4<br>8. EL 4<br>9. EL 4<br>10. EL 2 (2+ to 2−)<br>11. EL 2 (1+ to 2−)<br>12. EL 2 (2++ to 2−)	Recommendations graded based on Scottish Intercollegiate Guidelines Network methodology<br><br>Levels of evidence<br>1++: High-quality meta-analyses, systematic reviews of randomised controlled trials (RCTs) or RCTs with a very low risk of bias<br>1+: Well-conducted meta-analyses, systematic reviews or RCTs with a low risk of bias<br>1-: Meta-analyses, systematic reviews or RCTs with a high risk of bias<br>2++: High-quality systematic

	8. People who have bariatric surgery should have a postoperative follow-up care package within the bariatric surgery service for a minimum of two years. This should include monitoring nutritional intake, dietary and nutritional assessment, advice and support<br>9. People discharged from bariatric surgery service follow-up should undergo monitoring of nutritional status at least once a year as part of a shared care model of management<br>*Haematinics*<br>*Full blood count and ferritin*<br>10. Check full blood count and serum ferritin at regular intervals post-surgery<br>*Folate*<br>11. Check serum folate levels at regular intervals post-surgery<br>*Vitamin B12*<br>12. Check vitamin B12 levels at regular intervals following sleeve gastrectomy (SG), Roux-en-Y gastric bypass (RYGB) and malabsorptive procedures such as biliopancreatic diversion with duodenal switch (BPD/DS)<br>*Vitamin D, calcium and parathyroid hormone*<br>*Vitamin D*<br>13. Check serum 25-hydroxyvitamin D levels at regular intervals post-surgery<br>14. Serum 25-hydroxyvitamin D levels of 75 nmol/L or greater are considered sufficient<br>*Fat-soluble vitamins A, E and K*<br>*Vitamin A*<br>15. Consider checking serum vitamin A levels if patient reports steatorrhoea or symptoms of vitamin A deficiency, for example, night blindness or protein malnutrition<br>16. Check serum vitamin A levels at regular intervals following malabsorptive procedures such as BPD/DS<br>*Vitamin E*<br>17. Check serum vitamin E levels at regular intervals following malabsorptive procedures such as BPD/DS<br>18. Check serum vitamin E levels if unexplained anaemia or neuropathy<br>*Vitamin K*<br>19. Check vitamin K1 and PIVKA-II levels at regular intervals following malabsorptive procedures such as BPD/DS<br>*Trace minerals: zinc, copper, selenium and magnesium*	13. Grade B<br>14. Grade D<br>15. Grade D<br>16. Grade B<br>17. Grade B<br>18. Grade D<br>19. Grade B<br>20. Grade B<br>21. Grade C<br>22. Grade D<br>23. Grade D<br>24. Grade C<br>**Postoperative vitamin and mineral supplementation**<br>25. Grade B<br>26. Grade B<br>27. Grade D<br>28. Grade B<br>29. Grade D<br>30. Grade D<br>31. Grade C<br>32. Grade B<br>33. Grade C<br>34. Grade C<br>35. Grade D<br>36. Grade B<br>37. Grade C<br>38. Grade D<br>39. Grade D<br>40. Grade B<br>41. Grade B<br>42. Grade D	13. EL 2 (1+ to 3)<br>14. EL 4<br>15. EL 4 (2+ to 4)<br>16. EL 2 (1+ to 2)<br>17. EL 2 (1+ to 2+)<br>18. EL 4<br>19. EL 2 (1+ to 3)<br>20. EL 2 (1+ to 3)<br>21. EL 3 (2− to 3)<br>22. EL 4<br>23. EL 2 (2−)<br>24. EL 2 (2+)<br>**Postoperative vitamin and mineral supplementation**<br>25. EL 2 (1+ to 2−)<br>26. EL 2 (1+ to 2−)<br>27. EL 4 (1+ to 4)<br>28. EL 2 (1+ to 2−)<br>29. EL 4 (2 to 4)<br>30. EL 4 (2 to 4)<br>31. EL 2 (1− to 4)<br>32. EL 2 (1+ to 3)<br>33. EL 2 (1+ to 4)<br>34. EL 2 (1+ to 4)<br>35. EL 4<br>36. EL 2<br>37. EL 2<br>38. EL 4<br>39. EL 2 (2−)<br>40. EL 2 (1+ to 2−)<br>41. EL 2<br>42. EL 4	reviews of case control or cohort studies<br>High-quality case-control or cohort studies with a very low risk of confounding or bias and a high probability that the relationship is causal<br>2+: Well-conducted case-control or cohort studies with a low risk of confounding or bias and a moderate probability that the relationship is causal<br>2-: Case-control or cohort studies with a high risk of confounding or bias and a significant risk that the relationship is not causal<br>3: Non-analytical studies, e.g., case reports and case series<br>4: Expert opinion<br><br>Grades of recommendation<br>The grade of recommendation relates to the strength of supporting evidence and not the clinical importance of the recommendation<br>A: At least one meta-analysis, systematic review or RCT rated as 1++ and directly applicable to the target population; or a body

	Zinc<br>20. Check serum/plasma zinc levels at regular intervals following SG, RYGB or BPD/DS<br>21. Check serum copper levels at regular intervals following SG, RYGB or BPD/DS<br>Selenium<br>22. Check serum selenium levels if there is chronic diarrhoea, metabolic bone disease, unexplained anaemia or unexplained cardiomyopathy<br>23. Check serum selenium levels at regular intervals following RYGB<br>24. Check serum selenium levels at regular intervals following malabsorptive procedures such as BPD/DS<br><br>**Postoperative vitamin and mineral supplementation**<br>*Iron*<br>25. Following SG, RYGB or malabsorptive procedures such as BPD/DS, recommend that people take additional<br>elemental iron<br>26. Consider starting with 200-mg ferrous sulphate, 210-mg ferrous fumarate or 300-mg ferrous gluconate daily<br>and twice daily in menstruating women and adjust depending on blood results<br>*Folic acid*<br>27. Advise people to take a complete multivitamin and mineral supplement providing 400- to 800-µg folic acid per day<br>*Vitamin B12*<br>28. Following SG, RYGB or malabsorptive procedures such as BPD/DS, recommend routine supplementation with vitamin B12 intramuscular injections<br>*Calcium and vitamin D*<br>*Vitamin D*<br>29. Adjust vitamin D3 supplementation to maintain serum 25-hydroxyvitamin D levels of 75 nmol L−1 or higher<br>30. Maintenance levels of between 2000 and 4000 IU oral vitamin D3 per day may be required following SG and RYGB and higher following malabsorptive procedures such as BPD/DS<br>*Vitamins A, E and K*<br>*Vitamin A*			of evidence consisting principally of studies rated as 1+, directly applicable to the target population and demonstrating overall consistency of results<br>B: A body of evidence including studies rated as 2++, directly applicable to the target population and demonstrating overall consistency of results; or extrapolated evidence from studies rated as 1++ or 1+<br>C: A body of evidence including studies rated as 2+, directly applicable to the target population and demonstrating overall consistency of results; or<br>D: Extrapolated evidence from studies rated as 2++<br>Evidence level 3 or 4; or extrapolated evidence from studies rated as 2+

	31. Following RYGB, especially in people, consider that some may require additional routine oral vitamin A supplementation, especially if symptoms such as deterioration in night vision and dry eyes are present<br>32. Following malabsorptive procedures such as BPD/DS, recommend daily supplementation with additional oral vitamin A<br>*Vitamin E*<br>33. Following malabsorptive procedures such BPD/DS, recommend daily oral supplementation with additional vitamin E<br>*Vitamin K*<br>34. Following malabsorptive procedures such BPD/DS, recommend daily oral supplementation with additional vitamin K<br>*Water-miscible forms of fat-soluble vitamins*<br>35. Water-miscible forms of fat-soluble vitamins may improve absorption especially after malabsorptive procedures<br>*Zinc and copper*<br>36. Recommend a multivitamin and mineral containing at least the government recommended daily allowance for zinc<br>37. Following malabsorptive procedures such BPD/DS, the optimal level of zinc supplementation is not known but will be higher than that for RYGB or SG. We recommend starting with at least 30-mg oral zinc daily, which may be contained within the oral multivitamin and mineral supplement<br>38. Following RYGB, SG and BPD/DS, recommend complete multivitamin and mineral oral supplement containing 2-mg copper<br>*Selenium*<br>39. Recommend a complete multivitamin and mineral supplement containing selenium<br>40. Following malabsorptive procedures such as BPD/DS, additional routine oral supplementation with selenium may be needed to prevent deficiency<br>*Thiamine*<br>41. Recommend a complete multivitamin and mineral supplement containing at least government dietary recommendations for thiamine<br>42. Prescribe oral thiamine 200–300 mg daily, vitamin B co strong 1 or 2 tablets, three times a day to people with symptoms such as dysphagia, vomiting, poor dietary intake or fast weight loss			

Veterans Association /Department of Defense Clinical Practice Guideline for the Management of Adult Overweight and Obesity	**Comprehensive lifestyle interventions**<br>1. We recommend offering an in-person group or individual comprehensive lifestyle intervention that always includes behavioral, dietary, and physical activity components for patients with overweight or obesity.<br>2. There is insufficient evidence to recommend a specific number of sessions of a comprehensive lifestyle intervention for patients with overweight or obesity.<br>3. We suggest offering a comprehensive lifestyle intervention for weight maintenance to patients who have completed a comprehensive lifestyle intervention for weight loss.<br>4. We suggest offering an individual or group telephone-delivered comprehensive lifestyle intervention for weight loss, either as an alternative to or in conjunction with an in-person intervention.<br>5. There is insufficient evidence for or against offering a comprehensive lifestyle intervention for weight loss that uses technology as its primary mode of delivery.<br>**Physical activity component of comprehensive lifestyle intervention**<br>6. We suggest choosing one or more of the following as the physical activity component of a comprehensive lifestyle intervention: aerobic, resistance, and/or lifestyle physical activity.<br>**Dietary component of comprehensive lifestyle intervention**<br>7. We recommend offering patients a dietary approach that contributes to a negative energy balance to achieve weight loss as the dietary component of a comprehensive lifestyle intervention.<br>8. We suggest meal replacement (for example portion-controlled shake, protein bar, or meal) as an option to achieve negative energy balance as a component of a comprehensive lifestyle intervention.<br>**Long term pharmacotherapy**<br>9. We suggest offering prescribed pharmacotherapy (specifically liraglutide, naltrexone/bupropion, orlistat, or phentermine/topiramate) for long-term weight loss in patients with a body mass index ≥30 kg/m^2 and for those with a body mass index ≥27 kg/m^2 who also have obesity-associated conditions, in conjunction with a comprehensive lifestyle intervention.<br>10. There is insufficient evidence to recommend for or against offering phentermine monotherapy, benzphetamine, diethylpropion, or phendimetrazine, for short-term, long-term, or intermittent weight loss in patients with overweight or obesity.	1. Moderate<br>2. Low<br>3. Moderate<br>4. Low<br>5. Very low<br>6. Low<br>7. Moderate<br>8. Moderate<br>9. Moderate<br>10. Low<br>11. Low-very low<br>12. Low-very low<br>13. Very low<br>14. Very low<br>15 Moderate<br>16. Moderate<br>17. Moderate<br>18. Very low	1. Strong for<br>2. Neither for nor against<br>3. Weak for<br>4. Weak for<br>5. Neither for nor against<br>6. Weak for<br>7. Strong for<br>8. Weak for<br>9. Weak for<br>10. Neither for nor against<br>11. Weak against<br>12. Weak for<br>13. Weak for<br>14. Neither for nor against<br>15. Neither for nor against<br>16. Weak for<br>17. Neither for nor against<br>18. Weak for	The GRADE methodology framework was used to assess the certainty of the evidence and evidence to decision making (EtD) framework was used to assign a strength for each recommendation.<br><br>Relative strength of each recommendations was classified as 'Strong' or 'Weak' and also the direction of the recommendation ('Strong for', 'Weak for', 'no recommendation for or against', 'Weak for', 'Strong against')

	**Dietary supplements and nutraceuticals**<br>11. We suggest against using dietary supplements or nutraceuticals for clinically meaningful short-term weight loss or long-term weight management.<br>**Metabolic/bariatric procedures and devices**<br>12. We suggest offering the option of metabolic/bariatric surgery, in conjunction with a comprehensive lifestyle intervention, to patients with a body mass index of 30 kg/m^2 and type 2 diabetes mellitus.<br>13. We suggest offering the option of metabolic/bariatric surgery, in conjunction with a comprehensive lifestyle intervention, for long-term weight loss/maintenance and/or to improve obesity-associated conditions) in adult patients with a body mass index ≥ 40 kg/m^2 or those with body mass index ≥ 35 kg/m^2 with obesity-associated condition(s).<br>14. There is insufficient evidence to recommend for or against metabolic/bariatric surgery to patients over age 65.<br>15. There is insufficient evidence to recommend for or against percutaneous gastrostomy devices for weight loss in patients with obesity.<br>**Short-term weight loss**<br>16. We suggest offering intragastric balloons in conjunction with a comprehensive lifestyle intervention to patients with obesity (body mass index ≥30 kg/m^2) who prioritize short-term (up to six months) weight loss.<br>17. There is insufficient evidence to recommend for or against intragastric balloons for long-term weight loss to support chronic weight management or maintenance.<br>18. We suggest offering a low-carbohydrate diet over a low-fat diet as the dietary component of a comprehensive lifestyle intervention for patients who prioritise short-term (up to six months) weight loss.			
Clinical practice guidelines of the European Association for Endoscopic Surgery (EAES) on bariatric surgery: update 2020	**Indication for bariatric surgery**<br>1. Laparoscopic bariatric surgery should be considered for patients with BMI ≥40 kg/m^2 and for patients with BMI ≥35–40 kg/m^2 with associated comorbidities that are expected to improve with weight loss.<br>2. Laparoscopic bariatric/metabolic surgery should be considered for patients with BMI ≥ 30–35 kg/m^2 and type 2 diabetes and/or arterial hypertension with poor control despite optimal medical therapy.<br><br>**Preoperative work-up**<br>1. No recommendation can be made for either routine H. pylori eradication or	**Indication for bariatric surgery**<br>1. Moderate - Low<br>2. Moderate - Low<br>**Preoperative work-up**<br>1. Very low	**Indication for bariatric surgery**<br>1. Strong<br>2. Strong<br>**Preoperative work-up**<br>1. Conditional for either intervention or comparator	GRADE methodology was used for rating the evidence and the GRADE EtD framework was used to assign strength of recommendation.<br><br>All panel members took part in three web based Delphi

endorsed by IFSO-EC, EASO and ESPCOP	no eradication prior to bariatric surgery on the basis of available evidence.<br>2. Preoperative dietitian consultation should be considered for patients undergoing bariatric surgery.<br>3. Esophagogastroscopy can be considered as a routine diagnostic test prior to bariatric surgery.<br>4. Psychological evaluation can be considered before bariatric surgery.<br>A previous diagnosis of binge eating or depression may not be considered as an absolute contraindication to surgery.<br><br>**Perioperative management**<br>1. Screening for obstructive sleep apnea using the STOP-BANG criteria can be considered prior to bariatric surgery.<br>2. Perioperative Continuous Positive Airway Pressure (CPAP) should be considered in patients with severe obstructive sleep apnea syndrome who are undergoing bariatric surgery.<br>3. No recommendation can be made on the dose and duration of pharmacological thromboprophylaxis in patients after bariatric surgery.<br>4. Inferior vena cava filter is not recommended for thromboprophylaxis in patients undergoing bariatric surgery.<br>5. No recommendation for either an enhanced recovery after surgery (ERAS) protocol or standard care can be made on the basis of available evidence.<br>6. Perioperative multimodal analgesia with minimal opioid usage may be considered in patients undergoing bariatric surgery.<br><br>**Non-bypass procedures**<br>1. Adjustable gastric banding surgeries are associated with a high rate of reoperations for complications or conversion to another bariatric procedure for insufficient weight loss in the long term.<br>2. Sleeve gastrectomy may be preferred over adjustable gastric banding for weight loss and control/resolution of metabolic comorbidities.<br>3. Sleeve gastrectomy may offer improved short-term weight loss and resolution of type 2 diabetes compared to gastric plication. No signifcant differences are observed at mid-term. Long-term comparative data on weight loss and metabolic effects are, however, lacking.<br>4. There is insufficient evidence to recommend routine stapler line reinforcement (buttress, glues, suturing, clips) to reduce the leak rate.	2. Very low<br>3. Very low<br>4. Very low<br>**Perioperative management**<br>1. Very low<br>2. Very low-low<br>3. Moderate-low<br>4. Low<br>5. Very low<br>6. High-very low<br>**Non-bypass procedures**<br>1. N/A<br>2. Low<br>3. Very low<br>4. N/A<br>5. High-Low<br>6. Moderate-Very low<br>7. Very low<br>**Bypass procedures**<br>1. Low<br>2. Low<br>3. Moderate-very low<br>4.Moderate-low<br>5. Low<br>6. Very low-low	2. Strong<br>3. Conditional<br>4. Conditional<br>**Perioperative management**<br>1. Conditional<br>2. Strong<br>3. Conditional for either intervention or comparator<br>4. Strong<br>5. Conditional for either intervention or comparator<br>6. Conditional<br>**Non-bypass procedures**<br>1. Position statement<br>2. Conditional<br>3. Position statement<br>4. Position statement<br>5. Strong<br>6. Conditional<br>7. Position statement<br>**Bypass procedures**<br>1. Strong<br>2. Position statement<br>3. Position statement<br>4. Conditional<br>5. Conditional for	rounds for the draft recommendations.<br><br>GRADEpro Guideline Development Tool (GDT) software was used to create evidence tables, in the development of recommendations and in the Delphi process (PanelVoice 2.0 add-on).

	5. Staple line reinforcement in sleeve gastrectomy should be considered to reduce the risk of perioperative complications (overall mortality, bleeding).<br>6. A bougie size <36F compared to a bougie sized ≥36F may be recommended for calibration in sleeve gastrectomy as it is associated with greater weight loss in the mid-term.<br>7. More extensive antral resection (2–3 cm from the pylorus versus>5 cm antral preservation) potentially offers greater weight loss in the short term without a signifcant increase in post-operative complications. Long term data are, however, lacking.<br><br>**Bypass procedures**<br>1. Roux-en-Y Gastric Bypass (RYGB) should be preferred over adjustable gastric banding.<br>2. RYGB results in greater weight loss and control/remission of insulin resistance and type 2 diabetes compared to gastric plication.<br>3. RYGB ofers similar mid-term weight loss and control/remission of metabolic comorbidities compared to sleeve gastrectomy. Long-term comparative data are, however, lacking.<br>4. RYGB can be preferred over sleeve gastrectomy in patients with severe gastroesophageal refux disease and/or severe esophagitis.<br>5. No recommendation for either biliopancreatic diversion with duodenal switch (BPD/DS) or sleeve gastrectomy can be made on the basis of available comparative evidence.<br>6. With regard to mid-term weight loss there is no difference between BPD/DS and RYGB. BPD/DS is superior to RYGB for control/remission of type 2 diabetes. Long-term comparative data are, however, lacking.<br><br>**One anastomosis procedures**<br>1. OAGB may offer greater short-term weight loss compared to RYGB, gastric plication, adjustable gastric banding and sleeve gastrectomy. Long-term comparative data are, however, lacking. The effect on nutritional deficiencies remains controversial.<br>2. No recommendation on single-anastomosis duodeno-ileal switch (SADI-S) compared with one anastomosis gastric bypass (OAGB), BPD/DS, RYGB or sleeve gastrectomy can be made on the basis of available evidence.	**One anastomosis procedures**<br>1. High-very low<br>2. Low<br>**Revisional surgery**<br>1. Very low<br>**Postoperative care**<br>1. Low<br>2. Very low<br>3. Very low<br>4. Moderate<br>5. Very low<br>6. Very low<br>**Investigational procedures**<br>1. Very low<br>2. Very low	either intervention or comparator<br>6. Position statement<br>**One anastomosis procedures**<br>1. Position statement<br>2. Conditional for either intervention or comparator<br>**Revisional surgery**<br>1. Position statement<br>**Postoperative care**<br>1. Strong<br>2. Conditional<br>3. Strong<br>4. Strong<br>5. Strong<br>6. Strong<br>**Investigational procedures**<br>1. Position statement<br>2. Position statement	

	**Revisional surgery**<br>1. No evidence-based criteria for indication to revisional bariatric/metabolic surgery are available to date. The panel advises that the clinical decision to proceed to revisional bariatric/metabolic surgery be based on a complete multidisciplinary assessment of the patient, as recommended for the primary procedure.<br><br>**Postoperative care**<br>1. Scheduled multidisciplinary post-operative follow-up should be provided to every patient undergoing bariatric/metabolic surgery.<br>2. Treatment with ursodeoxycholic acid could be considered during the weight loss phase to prevent gallstones formation.<br>3. Micro and/or macronutrients supplementation is recommended after bariatric surgery according to the type of the procedure and to the deficiencies documented during the follow-up.<br>4. Proton-pump inhibitor (PPI) therapy should be given to patients undergoing bypass procedures for the prevention of marginal ulcers.<br>5. Postoperative nutritional and behavioral advice should be provided to patients undergoing bariatric surgery.<br>6. Pregnancy following bariatric surgery should be delayed during the weight loss phase.<br><br>**Investigational procedures**<br>1. For duodenal-jejunal bypass sleeves, aspiration devices, gastric electrical stimulation, vagal blockade and duodenal mucosal resurfacing, the quality of evidence was too low to provide any recommendations.<br>2. Endoluminal suturing procedures may have a role in the treatment of patients with obesity with BMI <40 kg/m^2.<br>Position statements do not constitute recommendations			
Overweight and obesity management NICE guideline [NG246]	**General principles of care**<br><br>Discussion, communication and follow up<br><br>**Discussion, communication and follow up**<br>1.1.1 Before or during any discussions, think about the wider determinants and the context of overweight and obesity. These include:<br>• general health and current comorbidities	**[F] Evidence reviews for the effectiveness of different diets in achieving and**	See rating system - explicit and specific strength of recommendations were not reported. Instead a controlled vocabulary was	GRADE framework for pairwise meta-analyses of interventional evidence<br>Modified GRADE for intervention studies analysed using network meta-analysis<br>GRADE-CERQual for

	• weight-related comorbidities, including family history of weight-related comorbidities<br>• weight history and previous experiences of managing overweight or obesity<br>• experiences of weight stigma<br>• impact of bullying and adverse childhood experiences<br>• practicality of addressing weight and readiness to engage with change<br>• developmental stage (for children and young people)<br>• ethnicity<br>• language<br>• socioeconomic status and financial constraints<br>• personal and family circumstances, including living arrangements and major life events<br>• recent pregnancy<br>• how any medicines the person is taking may affect their weight or appetite<br>• current or previous experiences of eating disorders or disordered eating<br>• psychosocial considerations (for example, depression, anxiety or sense of self-esteem or self-perception)<br>• physical disabilities<br>• neurodevelopmental conditions and special educational needs and disabilities (SEND). **[2025]**<br>1.1.2 Before discussing overweight, obesity or central adiposity, take into account:<br>• the context of the discussion or appointment and whether it is appropriate or important to discuss weight or take measurements on this occasion<br>• that the subject of weight may have been raised many times before<br>• your own feelings and sensitivities about weight<br>• that people can be affected by an eating disorder at any weight (see NICE's guideline on eating disorders)<br>• cultural factors that may be relevant.	**maintaining weight loss**<br><br>Evidence reviews underpinning recommendations 1.16.1 to 1.16.12 and research recommendations in the NICE guideline GRADE: quality of evidence ranged from high to very low, with only a small number of findings rated high quality and most being rated as low or very low.<br><br>**[I] Evidence review for psychological approaches to address weight stigma in children, young people,**	used to denote the strength of the recommendation.	qualitative evidence synthesis findings<br>Modified GRADE-CERQual for published qualitative evidence synthesis (QES)<br>Mixed methods studies - either same as intervention studies and qualitative studies or as agreed with NICE methods and economics team quality assessment lead<br><br>A decision-making committee was responsible for developing recommendations.<br><br>A controlled vocabulary was used to denote the strength of the recommendation: “Reflect the strength of the recommendation<br>In 'strong' recommendations for actions that should (or should not) be offered, use directive language such as 'offer' (or 'do not offer'), 'advise', or 'ask about'. If there is a closer balance between benefits and harms (activities or interventions that could be used), use 'consider' to reflect that the recommendation is 'weak'.''

	1.1.3 Ask permission to discuss overweight, obesity or central adiposity. If they do not wish to discuss it further on this occasion, respect the person's choice (and that of their family or carer, if relevant) and either explore the reason sensitively or delay discussion until an appropriate time. **[2025]**<br>1.1.4 Record the outcome of the discussion to ensure that subsequent healthcare professionals are aware that the matter has been raised, and know about the person's views and any actions already taken to manage overweight or obesity. **[2025]**<br>1.1.5 Ensure that all discussions linked to overweight, obesity and central adiposity are conducted in a sensitive, non-judgemental and person-centred manner by:<br>• using non-stigmatising language (for example, 'living with overweight')<br>• identifying and exploring the person's own preferred terms (and those of their family or carers, if relevant)<br>• focusing on improvements in health and wellbeing rather than simply talking about weight (for example, using terms like 'healthier weight' and 'improved health' may be more acceptable than 'preventing obesity' for some people)<br>• staying positive, supportive and solution-based<br>• taking into account the person's thoughts, views and cultural, religious or spiritual beliefs (and those of their family or carers, if relevant) about overweight and obesity management<br>• being mindful of the factors that prevent or restrict weight loss (for example, some medicines)<br>• taking into account the determinants and context of overweight and obesity (see recommendation 1.1.1)<br>1.1.6 Ensure that all written, visual and verbal communications with people living with overweight and obesity use non-stigmatising language and images and is tailored to any particular needs, such as Easy Read literature. Resources and advice that could help conduct conversations in a sensitive and positive way include: obesity image banks [2025].<br>**Equipment**<br>1.1.7 Equip specialist settings for treating people who are living with obesity with, for example, suitable seating and adequate weighing and monitoring equipment. **[2006]**	**and adults**<br><br>GRADE: quality of evidence ranged from moderate to very low for quantitative evidence<br>CERQual: Confidence in the evidence was low<br>The committee noted that the quantitative evidence ranged from moderate to very low quality, with most of the evidence being low quality.<br>The committee also noted that the evidence was assessed as low confidence due to		

	1.1.8 Ensure hospitals have access to suitable equipment – such as larger scanners and beds – when providing general care for people who are living with severe obesity. **[2006]**<br><br>**<u>Preventing overweight, obesity and central adiposity</u>**<br><br>**Managers and budget holders in local or regional strategic partnerships**<br><br>1.5.3 Ensure that systems are in place in primary care to implement the local overweight and obesity strategy. Enable health professionals with specific training, including public health practitioners working on their own or as part of multidisciplinary teams, to provide interventions to prevent and manage obesity. [2006]<br>1.5.4 Address barriers to healthcare professionals providing support and advice (such as internalised and societal weight stigma), particularly their concerns about the effectiveness of interventions, people's receptiveness and ability to change, and the impact of advice on relationships with the person. [2006, amended 2025]<br><br>**<u>Identifying and assessing overweight, obesity and central adiposity</u>**<br>1.9 Identifying and assessing overweight and obesity in adults<br>When to take and record measurements in adults<br>1.9.1 Avoid attributing all symptoms to overweight or obesity (diagnostic overshadowing). If the person is presenting with another health problem or condition (such as hip pain), address this problem or condition first before deciding whether it is appropriate to ask permission to discuss weight. [2025]<br>1.9.2 Ask for permission each time before discussing overweight, obesity or central adiposity and before taking measurements. See the section on discussion, communication and follow up for steps to think about before discussing overweight, obesity and central adiposity and how to ensure discussions are sensitive and non-judgemental. [2025]<br>1.9.4 Ensure that records are kept up to date and shared between providers, if possible and with permission, for people who have self-referred to overweight and obesity management interventions. [2025]<br>1.11 Discussing results and referral for adults	methodological limitations and adequacy<br><br>**[C] Evidence review for referral for bariatric surgery**<br><br>Evidence reviews underpinning recommendations 1.10.1 to 1.10.2 and 1.10.6 to 1.10.7 and research recommendations in the NICE guideline<br><br>GRADE: Quality of the evidence ranged from moderate to very low.<br>The committee noted that the evidence from RCTs, comparative observational		

	Discussing the results with adults<br>1.11.1 Give adults information about the severity of their overweight or obesity and central adiposity and the impact this has on their risk of developing other long-term conditions (such as type 2 diabetes, cardiovascular disease, hypertension, dyslipidaemia, certain cancers and respiratory, musculoskeletal and other metabolic conditions such as non-alcoholic fatty liver disease). [2006, amended 2022]<br>1.11.2 Offer advice and discuss the possibility of referral to an overweight and obesity management service with the person, taking into account their individual needs and preferences (see also recommendation 1.11.6). [2025]<br>1.11.3 Address the drivers of overweight and obesity (for example, social context, mental health and wellbeing, and stigma) if possible. Refer as needed for assessment for any comorbidities, or to other services such as social care, physiotherapy, eating disorder services or the NHS diabetes prevention programme. [2025]<br>1.11.4 Discuss and agree realistic, personalised health goals (and any other related goals such as clothes fitting better, taking part in active play with children or grandchildren, finding it easier to breathe when walking or climbing stairs, being able to tie shoelaces or fasten a standard-length seatbelt). Include the importance and wider benefits of making sustainable, long-term changes to dietary behaviours and increasing physical activity levels. [2025]<br>Choosing interventions with adults<br>1.11.5 Ensure that healthcare professionals involved in identifying overweight, obesity and central adiposity are familiar with the local overweight and obesity management pathway, including:<br>• local and national behavioural overweight and obesity management interventions and what these may involve<br>• links to support services, such as mental health support<br>• referral criteria and process for funded referrals<br>• the capacity of services<br>and are aware of services that are available locally and nationally. [2025]<br>1.11.6 Discuss and agree the type and level of intervention with adults who:<br>• are living with overweight or obesity or<br>• have increased health risk based on their waist-to-height ratio.<br><br>Take into account people's individual needs and preferences, and factors	studies, and systematic reviews of comparative observational studies ranged from moderate to very low quality, with the majority of the evidence being low to very low.<br><br>**[E] Evidence reviews for increasing uptake of weight management services in children, young people, and adults**<br><br>Evidence reviews underpinning recommendations 1.2.3 to 1.2.4, 1.11.2, 1.11.4, 1.11.7 to 1.11.11, 1.11.14 to		

	such as weight-related comorbidities, ethnicity, socioeconomic status, family medical history, eating disorders, disabilities including learning disabilities, neurodevelopmental conditions, and special educational needs and disabilities (SEND). See the sections on behavioural interventions, physical activity approaches, dietary approaches, medicines for overweight and obesity and surgical interventions. [2022, amended 2025]<br>1.11.7 Discuss any previous or ongoing overweight and obesity management interventions or attempts, including:<br>• acknowledging any progress the person has already made<br>• their positive or negative experiences with interventions<br>• any barriers, or concerns, they may have about making changes and meeting their personal goals<br>• any cultural and social context or assumptions about health and diet, and the impact of deviating from these to improve their health. [2025]<br>1.11.8 Identify available behavioural overweight and obesity management interventions that are:<br>• appropriate for the person, taking their preferences and previous experiences into account if possible<br>• culturally appropriate or have been adapted for different cultural communities and dietary practices<br>• tailored to specific demographic groups, such as men only or for older adults.<br><br>Explain how these may be beneficial (for example, peer support). [2025]<br>1.11.9 Inform people if there are any known costs associated with taking part in the intervention or continuing it after a funded referral period has ended. [2025]<br>1.11.10 Emphasise the person's choice in the referral. Refer them to an in-person individual or group intervention, or digital services according to preference and availability. [2025]<br>1.11.11 Give people information about more sources of long-term community or healthcare support (for example, provided by social prescribers, health coaches, pharmacists, local support groups, online groups or networks, friends and family, Talking Therapies, free healthcare-endorsed apps, national campaigns, and local community groups such as walking or gardening groups).	1.11.15, 1.12.3, 1.12.6 to 1.12.11, 1.13.1 to 1.13.5, 1.14.8, 1.14.24 to 1.14.31, 1.19.2, 1.19.19 to 1.19.21, 1.19.23 to 1.19.25 and research recommendations in the NICE guideline<br><br>The quantitative findings were assessed using GRADE. Of the 11 outcomes, three were moderate quality, six were low quality, and two were very low quality. Confidence ratings given to the qualitative evidence using the		

	These can be used while waiting for and alongside an overweight and obesity management intervention. See NICE's guideline on behaviour change: digital and mobile health interventions**. [2025]**<br>**Referring adults to specialist services**<br>1.11.12 Offer a higher level of intervention to people with weight-related comorbidities (see the section on assessing and managing comorbidities in adults). Adjust the approach depending on the person's clinical needs, for example for people with a BMI over 35 kg/m^2 who have recently developed diabetes or for people with a BMI of 50. [2022]<br>1.11.13 Consider referral to specialist overweight and obesity management services if:<br>• the underlying causes of overweight or obesity need to be assessed<br>• the person has complex disease states or needs that cannot be managed adequately in behavioural overweight and obesity management services (for example, the extra support needs of people with learning disabilities)<br>• less intensive management has been unsuccessful<br>• specialist interventions (such as a very-low-calorie diet) may be needed<br>• surgery or certain medicines is being considered.<br><br>For more information see specialist overweight and obesity services. [2006, amended 2025]<br>1.11.14 If the person declines a referral to an intervention:<br>• acknowledge and respect their choice, being aware that a person's decision to accept referral may be temporarily or permanently influenced by many factors, including the wider determinants of overweight and obesity<br>• either explore the reason sensitively or delay discussion until an appropriate time<br>• ensure they have the opportunity to discuss referral again in future<br>• give them information about other ways to make sustainable, long-term changes to their dietary behaviours and physical activity levels. **[2025]**<br>1.11.15 Give people the opportunity for a re-referral, as needed, taking into account that overweight and obesity management is a long-term process. **[2025]**	GRADE-CERQUAL criteria were considered good. The majority of themes were rated as high confidence (16 themes). There were also seven themes rated as moderate confidence. No themes were rated lower than moderate confidence.		

	**1.13 Behavioural overweight and obesity management interventions for adults**<br>**Encouraging adherence to behavioural overweight and obesity management interventions for adults**<br>These recommendations are for providers of behavioural overweight and obesity management services.<br>1.13.1 Discuss with the person any concerns or barriers that may affect their attendance and participation in the intervention (such as personal circumstances, cultural barriers, physical health needs, or neurodevelopmental or psychological factors), including those that affect their ability to make changes and their progress towards meeting their goals. Repeat these discussions during the course of the intervention if needed and acknowledge:<br>• any progress the person has made<br>• any positive or negative experiences with the intervention<br>• any cultural and social context or assumptions about health and diet, and the impact of deviating from these to improve their health. **[2025]**<br>1.13.2 If the person is facing difficulties that affect their attendance and participation in the intervention:<br>• discuss whether the programme is suitable for them at this time<br>• if it has not been possible to resolve their difficulties with the intervention agree what should happen next (for example, referral to another service, leave the intervention at an agreed time, or think about a re-referral at a later date). **[2025]**<br>1.13.3 Discuss with the person the importance of support from any other members of their household and others such as friends, family, and the other participants in group interventions. With their permission, talk to relevant household members about the intervention and how they can help. **[2025]**<br>1.13.4 Regularly review the person's health and progress they have made towards meeting their goals (including weight loss) and send feedback to the person's referring GP or healthcare professional (for adults who self-refer, ask permission before sending feedback to their GP). **[2025]**<br><br>**1.15 Physical activity approaches**<br>See also the recommendations on physical activity in the section on behavioural overweight and obesity management interventions.			

	**Staff qualifications**<br>1.15.1<br>Ensure staff leading supervised physical activity sessions are qualified and insured, for example, a physiotherapist or a practitioner member of the CIMSPA (Chartered Institute for the Management of Sport and Physical Activity). Ensure that people running children's sessions have a paediatric CPR qualification. **[2014, amended 2025]**<br>**Physical activity approaches for adults**<br>1.15.2 Encourage adults to increase their physical activity even if they do not lose weight as a result, because of the other health benefits it can bring. Encourage them to meet the recommendations in the UK Chief Medical Officers' physical activity guidelines for weekly activity. See NHS advice on treating obesity **[2006]**<br>1.15.3 Advise that to prevent obesity, most people may need to do 45 to 60 minutes of moderate-intensity physical activity a day, particularly if they do not reduce their energy intake. Advise people who have lived with obesity and have lost weight that they may need to do 60 to 90 minutes of activity a day to avoid regaining weight. (See NHS advice on treating obesity.) **[2006]**<br>1.15.4 Encourage adults to build up to the recommended activity levels for weight maintenance, using a managed approach with agreed goals. Recommend:<br>• activities as part of everyday life, such as brisk walking, gardening or cycling (see NICE's guideline on walking and cycling)<br>• supervised exercise programmes<br>• other activities, such as swimming, aiming to walk a certain number of steps each day, or stair climbing.<br><br>Take into account the person's current physical fitness and ability for all activities. Encourage people to also reduce the amount of time they spend inactive, such as leisure-based screen time. **[2006]**<br>**1.16 Dietary approaches**<br>Dietary approaches for all ages<br>1.16.1 Use a flexible and individualised approach to tailor dietary interventions to achieve nutritional balance while reducing energy intake, taking into account:<br>• food preferences (including cultural preferences)			

	• personal circumstances (such as home environment and family finances)<br>• any comorbidities (such as eating disorders or disordered eating, type 1 diabetes, inflammatory bowel disease or non-alcoholic fatty liver disease)<br>• any restrictions in the range of foods they eat (for example because of neurodiversity, sensory problems, or coeliac disease)<br>• that in many cases weight regain may happen. [2025]<br>1.16.2 Encourage people to improve their dietary intake even if this does not result in them losing weight, because there can be other health benefits (for example, improved lipid profile and reduced risk of type 2 diabetes and cardiovascular disease). [2025]<br>1.16.3 Ensure that dietary approaches for adults to support overweight and obesity management keep the person's total energy intake below their energy expenditure (also called an energy deficit or calorie deficit). This could be done by lowering specific macronutrient content (for example, low-fat or low-carbohydrate diets) or using other methods to limit overall energy intake. [2025]<br>1.16.4 Ensure that dietary approaches for children and young people keep their total energy intake at or below the recommended daily calorie intake for their age and sex, depending on their level of overweight or obesity and any weight-related comorbidities. Focus on healthy eating rather than on overly restrictive diets. See the Scientific Advisory Committee on Nutrition guidance on feeding young children aged 1 to 5 years. [2025]<br>1.16.5 Ensure that any dietary approaches that maintain an energy deficit are offered with support (for example by an appropriately trained healthcare professional such as a Registered dietitian or UKVRN registered nutritionist administered by the Association for Nutrition) and follow up to help people maintain any weight loss in the long term. [2025]<br>1.16.6 Encourage people to eat a nutritionally balanced diet in the long term, consistent with other healthy eating advice. See the NHS Eatwell guide. [2025]<br>1.16.7 Advise people not to use restrictive diets that are nutritionally unbalanced, because they are ineffective in the long term and can be harmful. [2025]<br><br>**Low-energy and very-low-energy diets for adults**<br>1.16.8 Consider low-energy diets (800 to 1,200 kilocalories per day, also known as low-calorie diets) only as part of a multicomponent overweight and obesity			

	management strategy with long-term support within a specialist overweight and obesity management service (or other services for the management of long-term conditions such as type 2 diabetes) for people:<br>• living with obesity (with or without diabetes) **or**<br>• living with overweight and with type 2 diabetes.<br><br>See the recommendations on classifying overweight, obesity and central adiposity in adults. **[2025]**<br>1.16.9 Consider very-low-energy diets (under 800 kilocalories per day, also known as very-low-calorie diets) only as part of a multicomponent strategy within a specialist overweight and obesity management service, for people who:<br>• are living with obesity **and**<br>• have a clinically assessed need to lose weight rapidly (for example, to make surgery safer and more feasible). **[2025]**<br>1.16.10 Do not use low-energy diets or very-low-energy diets as a long-term strategy to manage obesity. **[2025]**<br>1.16.11 Ensure that low-energy and very-low-energy diets:<br>• are nutritionally complete<br>• last no more than 12 weeks<br>• include ongoing clinical support and supervision, with access to support from an appropriately trained Registered dietitian or UKVRN registered nutritionist administered by the Association for Nutrition, and advice on reintroducing a wider range of foods (for example, moving on to a nutritionally balanced diet for long-term and sustainable weight loss maintenance). **[2025]**<br>1.16.12 Before starting someone on a low-energy or very-low-energy diet as part of a multicomponent overweight and obesity management strategy:<br>• Explain that this is a restrictive diet with a specific health goal (such as improvement in diabetes) and risks (such as weight cycling, weight regain and potential adverse events, and for very-low-energy diets also the risk of constipation, fatigue and hair loss).<br>• Explain that this is not a long-term overweight and obesity management strategy in its own right as it must be followed by lifelong dietary energy intake control and appropriate physical activity levels.<br>• Discuss:			

- that weight regain is likely to happen, and if it does it is not because they or their healthcare professional have 'failed'
    - reintroducing a wider range of foods after a low-energy or very-low-energy diet
    - the options for long-term weight loss maintenance support or therapies (including nutritional advice, physical activity, medicines or surgery) if weight regain happens.
- Offer assessment and counselling if they may have eating disorders or other mental health issues, to ensure the diet is appropriate for them.
- Review any medicines they are taking and discuss any changes that may need to be made. **[2025]**

1.17 Medicines for overweight and obesity
Medicines for adults
This section contains a table of medicines for weight management, which includes NICE technology appraisal guidance.
1.17.1 After dietary, exercise and behavioural approaches have been started and evaluated in adults living with overweight or obesity, see the weight management medicines options listed in table 1. **[2006, amended 2025]**
1.17.2 All medicines for weight management should be used alongside a reduced-calorie diet and increased physical activity. **[2025]**
1.17.3 Make the decision to start medicines after discussing them with the person, and discussing the potential impact on their motivation. Arrange information, support and counselling on additional diet, physical activity and behavioural strategies when medicines are prescribed, and give information on patient support programmes. **[2006, amended 2014]**
Table 1 Medicines options for weight management in adults

	**Tirzepatide**	**Semaglutide**	**Liraglutide**	**Orlistat**
For more detail see	NICE's technology appraisal guidance on tirzepatide for managing overweight and	NICE's technology appraisal guidance on semaglutide for managing overweight and obesity (TA875, March 2023)	NICE's technology appraisal guidance on liraglutide for managing overweight and obesity (TA664, December 2020)	There is no NICE technology appraisal guidance on orlistat

	obesity (TA1026, December 2024)			
For adults with	An initial BMI of at least 35 kg/m² and at least 1 weight-related comorbidity.	At least 1 weight-related comorbidity **and**:<br>• an initial BMI of 35.0 kg/m² or more,<br>**or**<br>• an initial BMI of 30.0 kg/m² to 34.9 kg/m² and who meet the criteria for referral to specialist overweight and obesity management services.	An initial BMI of 35 kg/m² or more **and** non-diabetic hyperglycaemia **and** a high risk of cardiovascular disease.	A BMI of 30 kg/m² or more **or** a BMI of 28 kg/m² or more and associated risk factors. (orlistat summary of product characteristics [SPC])
Setting	Prescribed in primary care or a specialist overweight and obesity management service.	Prescribed in a specialist overweight and obesity management service.	Prescribed in secondary care by a specialist overweight and obesity management service.	Prescribed in all settings and available in a lower dose from a pharmacy.
Route and frequency	Weekly subcutaneous injection.	Weekly subcutaneous injection.	Daily subcutaneous injection.	Oral capsule, up to 3 times a day.
When to stop	If less than 5% of the	Consider stopping if less than 5% of	Stop after 12 weeks on the 3.0 mg/day	Stop after 12 weeks if

<table>
<tr>
<td></td>
<td>
<table>
<tr>
<td>treatment</td>
<td>initial weight has been lost after 6 months on the highest tolerated dose, decide whether to continue treatment, taking into account the benefits and risks of treatment for the person.</td>
<td>the initial weight has been lost after 6 months of treatment.</td>
<td>dose if at least 5% of the initial body weight has not been lost.</td>
<td>at least 5% of the initial body weight has not been lost.</td>
</tr>
</table>
Semaglutide and liraglutide are recommended for use within specialist weight management services, which are usually accessed for up to 2 years.
Non-diabetic hyperglycaemia is defined as a haemoglobin A1c level of 42 mmol/mol to 47 mmol/mol (6.0% to 6.4%) or a fasting plasma glucose level of 5.5 mmol/litre to 6.9 mmol/litre.
Continued prescribing and withdrawal for adults
1.17.4 If there is concern about micronutrient intake adequacy, consider a supplement providing the reference nutrient intake for all vitamins and minerals, particularly for older people (who may be at risk of malnutrition) and young people (who need vitamins and minerals for growth and development). [2006]
1.17.5 Offer support to help maintain weight loss to people who are stopping weight management medicines. [2006]
Monitoring weight management medicines in adults
1.17.6 Monitor the effect of medicines and reinforce behavioural advice and adherence through regular review. [2006, amended 2014]
1.17.7 When agreeing goals with someone with type 2 diabetes, take into account that their weight loss may be slower than that of people without the condition. Review their goals regularly. [2006]
</td>
<td></td>
<td></td>
<td></td>
</tr>
</table>

	**1.18 Surgical interventions**<br>**When to refer adults for assessment for bariatric surgery**<br>1.18.1 Offer adults a referral for a comprehensive assessment by specialist overweight and obesity management services providing multidisciplinary management of obesity, to see whether bariatric surgery is suitable for them if they:<br>• have a BMI of 40 kg/m^2 or more, or between 35 kg/m^2 and 39.9 kg/m^2 with a significant health condition that could be improved if they lost weight (see box 2 for examples) **and**<br>• agree to the necessary long-term follow up after surgery (for example, lifelong annual reviews). **[2023]**<br>**Box 2 Examples of common health conditions that can improve after bariatric surgery**<br>Some conditions that can improve after bariatric surgery include:<br>• cardiovascular disease<br>• hypertension<br>• idiopathic intracranial hypertension<br>• non-alcoholic fatty liver disease with or without steatohepatitis<br>• obstructive sleep apnoea<br>• type 2 diabetes.<br>These examples are based on the evidence identified for this guideline and the list is not exhaustive.<br>**When to offer expedited assessment**<br>1.18.3 Offer an expedited assessment for bariatric surgery to people:<br>• with a BMI of 35 kg/m^2 or more who have recent-onset (diagnosed within the past 10 years) type 2 diabetes **and**<br>• as long as they are also receiving, or will receive, assessment in a specialist overweight and obesity management service. **[2014]**<br>1.18.4 Consider an expedited assessment for bariatric surgery for people:<br>• with a BMI of 30 to 34.9 kg/m^2 who have recent-onset (diagnosed within the past 10 years) type 2 diabetes **and**<br>• who are also receiving, or will receive, assessment in a specialist overweight and obesity management service. **[2014]**<br>**Initial assessment and discussions with the multidisciplinary team**<br>1.18.6 Ensure the multidisciplinary team within a specialist overweight and obesity management service includes or has access to health and social care			

	professionals who have expertise in conducting medical, nutritional, psychological and surgical assessments in people living with obesity and are able to assess whether surgery is suitable. **[2023]**<br>1.18.7 Carry out a comprehensive, multidisciplinary assessment for bariatric surgery based on the person's needs. As part of this, assess:<br>• the person's medical needs (for example, existing comorbidities)<br>• their nutritional status (for example, dietary intake, and eating behaviours)<br>• any psychological needs that, if addressed, would help ensure surgery is suitable and support adherence to postoperative care requirements<br>• their previous attempts to manage their weight, and any past response to an overweight and obesity management intervention (such as one provided by a specialist overweight and obesity management service)<br>• any other factors that may affect their response after surgery (for example, language barriers, learning disabilities and neurodevelopmental conditions, deprivation and other factors related to health inequalities)<br>• whether any individual arrangements need to be made before the day of the surgery (for example if they need extra dietary or psychological support, or support to manage existing or new comorbidities)<br>• fitness for anaesthesia and surgery. **[2023]**<br>1.18.8 The hospital specialist or bariatric surgeon should discuss the following with people who are thinking about having bariatric surgery:<br>• the potential benefits<br>• plans for conception and pregnancy (for women and trans and non-binary people of childbearing age)<br>• the longer-term implications and requirements of surgery<br>• the potential risks, including perioperative mortality, and complications.<br><br>Include the person's family and carers in the discussion, if appropriate. **[2006, amended 2023]**<br>1.18.9 Choose the surgical intervention jointly with the person, taking into account:<br>• the severity of obesity and any comorbidities<br>• the best available evidence on effectiveness and long-term effects<br>• the facilities and equipment available<br>• the experience of the surgeon who would perform the operation. **[2006]**			

	1.18.10 Give the person information on:<br>• appropriate dietary intake after the bariatric procedure<br>• monitoring their macronutrient and micronutrient status<br>• individualised nutritional supplementation, and sources of support and guidance for long-term weight loss and weight maintenance<br>• patient support groups. **[2006, amended 2023]**<br>**Preoperative assessment and discussions**<br>1.18.11 Carry out a comprehensive preoperative assessment of any psychological or clinical factors that may affect adherence to postoperative care requirements (such as changes to dietary intake, eating behaviours and taking nutritional supplements) before performing surgery. **[2006, amended 2014]**<br>**Medicines while waiting for surgery**<br>1.18.12 Medicines may be used to maintain or reduce weight before surgery for people who have been recommended surgery, if the waiting time is excessive. See the section on medicines for overweight and obesity. **[2006, amended 2023]**<br>**Multidisciplinary team qualifications and hospital equipment in surgical settings**<br>1.18.13 The surgeon in the multidisciplinary team should have:<br>• had relevant, supervised training<br>• specialist experience in bariatric surgery. **[2006, amended 2014]**<br>1.18.14 Ensure the multidisciplinary team carrying out bariatric surgery can provide:<br>• preoperative assessment, including a risk-benefit analysis that includes preventing complications of obesity<br>• specialist assessment for eating disorders (and if appropriate, referral or signposting to specialist eating disorder services)<br>• information on the different procedures, including potential weight loss and possible risks<br>• regular postoperative assessment, including specialist dietetic and surgical follow up (see recommendation 1.18.17 on postoperative and longer-term follow-up care)<br>• management of comorbidities			

	• specialist psychological support before and after surgery (for example, a psychological assessment before surgery and, if appropriate, referral to specialist mental health services either before or after surgery)<br>• information on plastic surgery (such as apronectomy) if appropriate. **[2006, amended 2023]**<br>1.18.15 Hospitals undertaking bariatric surgery should ensure there is access to, and staff trained to use, suitable equipment, including but not limited to weighing scales, blood pressure cuffs, theatre tables, walking frames, commodes, hoists, bed frames, pressure-relieving mattresses and seating suitable for people having bariatric surgery. **[2006, amended 2023]**<br>1.18.16 Only surgeons with extensive experience should undertake revisional surgery (if the first operation has not been effective) in specialist centres because of the higher rate of complications and increased mortality of revision surgery compared with primary surgery. **[2006]**<br>**Postoperative and longer-term follow-up care**<br>1.18.17 Offer people who have had bariatric surgery a follow-up care package for a minimum of 2 years within the bariatric service. Include:<br>• monitoring nutritional intake, (including protein and vitamins) and mineral deficiencies<br>• monitoring for comorbidities<br>• medications review<br>• individualised dietary and nutritional assessment, advice and support<br>• advice and support on physical activity<br>• psychological support tailored to the person<br>• information about professionally led or peer-support groups. **[2014]**<br>1.18.18 After discharge from follow up by the bariatric surgery service, ensure people are offered at least annual monitoring of nutritional status and appropriate supplementation after bariatric surgery, as part of a shared-care model with primary care. **[2014]**<br><br>**Digital technologies for delivering multidisciplinary weight-management services to adults**<br><br>NICE has published early value assessment guidance on technologies for delivering multidisciplinary weight-management services to adults that can be used in the NHS, while more evidence is generated. These technologies			

	provide multidisciplinary programmes to increase physical activity levels and improve eating behaviour and diet. Some of these technologies can also be used to prescribe and monitor weight management medicine. See NICE's early value assessment on digital technologies for delivering multidisciplinary weight-management services.			
